# Supplementary material for: Morally injurious events and post-traumatic embitterment disorder in UK health and social care professionals during COVID-19: a cross-sectional web survey
Source: BMJ Open. 2022 May 6;12(5):e054062. doi: 10.1136/bmjopen-2021-054062 (PMC9082726; doi:10.1136/bmjopen-2021-054062)
Supplement: Supplementary data [file bmjopen-2021-054062supp001.pdf]

Supplementary tables

Supplementary table 1.
Socio-demographic characteristics of participants, and their association with PTED and the MIES subscales (95% CI with 2,000 bootstrapped samples)

	N	%	PTED	*p*	Effect size	Transgressions-others	*p*	Effect size	Transgressions-self	*p*	Effect size	Betrayal	*p*	Effect size
**Gender**[a]														
Male	98	24.5%	1.17 ±1.0	0.493	0.08	3.07 ±1.53	0.484	0.08	2.21 ±1.17	0.668	0.05	2.86 ±1.43	0.450	0.09
Female	302	75.5%	1.10 ±0.87			2.95 ±1.47			2.15 ±1.24			2.74 ±1.36		
**Professional role**[b]														
Allied health	58	14.5%	0.96 ±0.92	0.215	0.01	2.71 ±1.44	0.374	0.00	1.90 ±1.05	0.021[c]	0.04	2.55 ±1.41	0.589	0.00
Primary care practitioner	63	15.8%	1.04 ±0.91			3.07 ±1.45			2.32 ±1.27			2.87 ±1.42		
Managerial	41	10.3%	0.96 ±0.87			2.66 ±1.58			1.84 ±1.25			2.60 ±1.63		
Health info/science	37	9.3%	1.38 ±1			3.18 ±1.53			2.40 ±1.27			2.89 ±1.43		
Psychological	27	6.8%	1.11 ±0.75			2.83 ±1.32			1.79 ±.98			2.56 ±1.08		
Corporate	65	16.3%	1.07 ±0.78			3.08 ±1.48			2.13 ±1.09			2.81 ±1.38		
Clinical support	105	26.3%	1.23 ±0.95			3.14 ±1.50			2.43 ±1.29			2.92 ±1.30		
**Mental health**[a]														
Current mental health diagnoses	97	24.3%	1.52 ±0.96	<0.001[d]	0.64	3.20 ±1.57	0.100	0.20	2.31 ±1.30	0.177	0.15	3.10 ±1.40	0.013[e]	0.31
No current mental health diagnoses	303	75.8%	0.98 ±0.84			2.91 ±1.45			2.12 ±1.20			2.68 ±1.36		
**Ethnicity**[a]														
White	252	63%	1.09 ±0.90	0.628	0.07	2.93 ±1.54	0.390	0.13	2.08 ±1.20	0.124	0.23	2.77 ±1.38	0.951	0.01

Ethnic minorities	53	13.3%	1.15 ±0.93	3.13 ±1.38	2.36 ±1.16	2.76 ±1.40

Mean ±SD are reported. a Independent samples t-test reporting Cohen's *d* effect size, b One-way ANOVA reporting ω^2 effect size, c Welch's $F(6, 138.418) = 2.579$, p=0.021, d Mean difference -0.54 BCA 95% CI [-0.74, -0.34] t(398) = -5.33, p<0.001, e Mean difference -0.40 BCA 95% CI [-0.72, -0.07] t(293) = -2.50, p=0.013[1]

[1] Non-parametric Mann Whitney-U and Kruskal Wallis-H tests were also conducted. There were no differences in findings.

Supplementary table 2.
Severity and frequency of endorsement of items on the MIES and PTED scale in UK health and social care workers

MIES Item-level endorsement	Raw frequency (N)	% (95% CI)
I saw things that were morally wrong	179	44.8% (39.8 to 49.8%)
I am troubled by having witnessed others immoral acts	167	41.8% (36.9 to 46.8%)
I acted in ways that violated by own moral code or values	76	19% (15.3 to 23.2%)
I am troubled by having acted in ways that violated by own moral code or values	94	23.5% (19.4 to 28%)
I violated by own morals by failing to do something that I felt I should have done	83	20.8% (16.9 to 25.1%)
I am troubled because I violated by own morals by failing to do something that I felt I should have done	78	19.5% (15.7 to 23.7%)
I feel betrayed by superiors who I once trusted.	161	40.3% (35.4 to 45.2%)
I feel betrayed by fellow colleagues who I once trusted.	124	31% (26.5 to 35.8%)
I feel betrayed by others outside my work organisation who I once trusted.	158	39.5% (34.7 to 44.5%)
**PTED Item-level endorsement**	**Raw frequency (N)**	**% (95% CI)**
Any item endorsed by at “partially true” or greater level	293	73.3% (68.6 to 77.5%)
that hurt my feelings and caused considerable embitterment	142	35.5% (30.8 to 40.4%)
that led to a noticeable and persistent negative change in my mental well-being	167	41.8% (36.9 to 46.8%)
that I see as very unjust and unfair	170	42.5% (41.8 to 36.9%)
about which I have to think over and over again	167	41.8% (35.8 to 31%)
that causes me to be extremely upset when I am reminded of it	143	35.8% (31 to 40.7%)
that triggers me to harbour thoughts of revenge	44	11% (8.1 to 14.5%)
for which I blame and am angry with myself	87	21.8% (17.8 to 26.1%)
that led to the feeling that there is no sense to strive to make an effort	119	29.8% (25.3 to 34.5)
that makes me to frequently feel sullen or unhappy	151	37.8% (33 to 42.7%)
that impaired my overall physical well being	138	34.5% (29.8 to 39.4%)
that causes me to avoid certain places or persons so as to not be reminded of them	120	30% (25.5 to 34.8%)
that makes me feel helpless and disempowered	141	40.5% (35.7 to 45.5%)

that triggers feelings of satisfaction when I think that the responsible party having to experience a similar situation	76	19% (15.3 to 23.2%)
that led to a considerable decrease in my strength and drive	164	41% (36.1 to 46%)
that made that I am more easily irritated than before	176	44% (39.1 to 49%)
that makes that I must distract myself in order to experience a normal mood	160	40% (35.2 to 45%)
that made me unable to pursue occupational and/or family activities as before	176	44% (39.1 to 49%)
that caused me to draw back from friends and social activities	179	44.8% (39.8 to 49.8%)
which frequently evokes painful memories	118	29.5% (25.1 to 34.2%)

For item-level endorsement, each MIES item was coded as endorsed if the participant responded either “slightly agree” “moderately agree” or “strongly agree” and each PTED item was coded as endorsed if the participant responded either “partially true” “very much true” or “extremely true”.

Supplementary table 3.

Coefficients from the final step in hierarchical multiple regression analyses predicting PTED and transgressions-others, transgressions-self, and betrayal scores with 95% bias corrected and accelerated confidence intervals (2000 samples) (N=394).

	PTED			Transgressions-others			Transgressions-self			Betrayal		
Predictors	β	*B* 95% CI	*p*	β	*B* 95% CI	*p*	β	*B* 95% CI	*p*	β	*B* 95% CI	*p*
Age	-0.01	(-0.01 to 0.01)	0.789	0.04	(-0.01 to 0.02)	0.555	0.03	(-0.01 to 0.02)	0.587	0.04	(-0.01 to 0.02)	0.392
Current mental health diagnoses	0.12	(0.07 to 0.46)	**0.006**	-0.01	(-0.43 to 0.28)	0.835	-0.04	(-0.39 to 0.18)	0.512	0.04	(-0.25 to 0.45)	0.404
Social desirability	-0.02	(-0.03 to 0.02)	0.729	-0.08	(-0.10 to 0.01)	0.128	-0.21	(-0.14 to -0.05)	**0.002**	-0.13	(-0.11 to -0.01)	**0.012**
Years in role	-0.02	(-0.02 to 0.01)	0.747	-0.10	(-0.05 to 0.01)	0.110	-0.08	(-0.04 to 0.01)	0.185	0.01	(-0.02 to 0.02)	0.922
Allied	-0.12	(-0.60 to -0.09)	**0.036**	-0.12	(-0.93 to -0.09)	**0.020**	-0.16	(-0.87 to -0.15)	**0.002**	-0.10	(-0.71 to 0.00)	**0.036**
Primary care practitioner	-0.11	(-0.54 to -0.04)	**0.030**	-0.07	(-0.72 to 0.14)	0.201	-0.09	(-0.70 to 0.12)	0.185	-0.05	(-0.69 to 0.20)	0.434
Managerial	-0.04	(-0.37 to 0.15)	0.398	-0.11	(-1.12 to 0.04)	0.077	-0.13	(-0.91 to -0.03)	**0.028**	-0.04	(-0.64 to 0.39)	0.476
Health info/science	0.06	(-0.10 to 0.46)	0.157	0.03	(-0.40 to 0.69)	0.573	-0.01	(-0.47 to 0.43)	0.939	0.01	(-0.43 to 0.57)	0.819
Psychological	0.04	(-0.12 to 0.50)	0.307	-0.02	(-0.67 to 0.37)	0.581	-0.09	(-0.92 to 0.04)	0.061	-0.01	(-0.47 to 0.46)	0.934
Corporate	-0.02	(-0.29 to 0.21)	0.681	0.00	(-0.49 to 0.42)	0.978	-0.08	(-0.58 to 0.09)	0.142	-0.02	(-0.43 to 0.38)	0.747
Occupational stressors	0.38	(0.08 to 0.12)	**0.006**	0.32	(0.10 to 0.18)	**0.002**	0.26	(0.06 to 0.13)	**0.002**	0.29	(0.08 to 0.15)	**0.006**
Optimism	0.03	(-0.04 to 0.06)	0.681	-0.01	(-0.09 to 0.08)	0.876	0.15	(-0.01 to 0.13)	0.073	.007	(-0.04 to 0.14)	0.386
Pessimism	-0.01	(-0.05 to 0.05)	0.843	0.01	(-0.06 to 0.08)	0.888	-0.04	(-0.08 to 0.05)	0.644	.005	(-0.03 to 0.10)	0.470
Resilient coping style	0.06	(-0.01 to 0.06)	0.265	0.15	(0.03 to 0.16)	**0.014**	0.02	(-0.05 to 0.06)	0.746	.005	(-0.03 to 0.10)	0.410
Distributive justice	0.13	(0.01 to 0.04)	**0.024**	0.08	(-0.01 to 0.06)	0.163	0.08	(-0.01 to 0.05)	0.220	.007	(-0.01 to 0.06)	0.217
Procedural justice	-0.29	(-0.07 to -0.04)	**0.006**	-0.23	(-0.11 to -0.03)	**0.002**	-0.23	(-0.09 to -0.03)	**0.002**	-0.35	(-0.13 to -0.07)	**0.006**

CFC-I	0.14	(0.01 to 0.03)	**0.012**	0.03	(-0.02 to 0.03)	0.565	0.12	(0.01 to 0.04)	**0.037**	0.05	(-0.01 to 0.03)	0.355
CFC-F	0.11	(0.01 to 0.03)	**0.036**	0.08	(-0.01 to 0.04)	0.130	0.07	(-0.01 to 0.03)	0.262	0.05	(-0.01 to -0.03)	0.331
Self-esteem	-0.28	(-.07 to -.02)	**0.006**	-0.09	(-0.07 to 0.01)	0.207	-0.19	(-0.07 to -0.01)	**0.016**	-0.09	(-0.06 to 0.02)	0.241

Reference groups: Current mental health diagnoses (none), Professional role (clinical support role)
PTED = post-traumatic embitterment disorder; transgressions-others = Moral injury transgression by others; transgressions-self = Moral injury transgression by self; betrayal = Moral injury betrayal; CFC-I = Consideration of future consequences-Immediate; CFC-F = Consideration of future consequences-Future.
Significant p values are in bold.

Supplementary table 4.
Coefficients from the final step in hierarchical regression analyses using ***only significant predictors*** *of PTED, transgressions-others, transgressions-self, and betrayal scores with 95% bias corrected and accelerated confidence intervals (2000 samples) (N=394 to 396).*

PTED $_a$				Transgressions-others $_a$				Transgressions-self $_b$				Betrayal $_b$			
Predictors	β	*B* 95% CI	*p*	Predictors	β	*B* 95% CI	*p*	Predictors	β	*B* 95% CI	*p*	Predictors	β	*B* 95% CI	*p*
Current mental health diagnoses	0.12	(0.06 to 0.50)	**0.006**	Allied	-0.13	(-0.94 to -0.13)	**0.018**	Allied	-0.15	(-0.82 to -0.21)	**0.005**	Allied	-0.10	(-0.78 to 0.01)	**0.043**
Allied	-0.12	(-0.55 to -0.03)	**0.019**	Primary care practitioner	-0.07	(-0.77 to 0.19)	0.206	Primary care practitioner	-0.08	(-0.63 to 0.12)	0.190	Primary care practitioner	-0.05	(-0.63 to 0.23)	0.317
Primary care practitioner	-0.12	(-0.51 to -0.03)	**0.024**	Managerial	-0.10	(-0.99 to 0.10)	0.086	Managerial	-0.10	(-0.84 to 0.03)	0.064	Managerial	-0.05	(-0.66 to 0.29)	0.385
Managerial	-0.03	(-0.35 to 0.13)	0.466	Health info/science	0.04	(-0.30 to 0.77)	0.369	Health info/science	0.01	(-0.44 to 0.46)	0.991	Health info/science	0.01	(-0.38 to 0.44)	0.882
Health info/science	0.06	(-0.07 to 0.50)	0.188	Psychological	0.01	(-0.49 to 0.48)	0.976	Psychological	-0.08	(-0.85 to 0.07)	0.080	Psychological	-0.01	(-0.53 to 0.33)	0.724
Psychological	0.04	(-0.16 to 0.45)	0.344	Corporate	0.02	(-0.31 to 0.47)	0.690	Corporate	-0.06	(-0.52 to 0.13)	0.214	Corporate	-0.02	(-0.47 to 0.28)	0.675

Corporate	-0.12	(-0.28 to 0.19)	0.727	Occupational stressors	0.34	(0.11 to 0.19)	**0.001**	Social desirability	-0.20	(-0.13 to 0.04)	**0.001**	Social desirability	-0.14	(-0.11 to -0.02)	**0.007**
Occupational stressors	0.39	(0.08 to 0.12)	**0.002**	Resilient coping style	0.11	(0.01 to 0.12)	**0.021**	Occupational stressors	0.27	(0.06 to 0.14)	**0.001**	Occupational stressors	0.31	(0.09 to 0.16)	**0.001**
Distributive justice	0.14	(0.01 to 0.04)	**0.006**	Procedural justice	-0.21	(-0.10 to -0.04)	**0.001**	Procedural justice	-0.16	(-0.07 to -0.01)	**0.001**	Procedural justice	-0.32	(-0.12 to -0.07)	**0.001**
Procedural justice	-0.28	(-0.07 to -0.03)	**0.002**					CFC-I	0.10	(-0.01 to 0.03)	**0.046**				
CFC-I	0.13	(0.01 to 0.03)	**0.008**					Self-esteem	-0.04	(-0.03 to 0.01)	0.427				
CFC-F	0.12	(0.01 to 0.03)	**0.006**												
Self-esteem	-0.25	(-0.05 to -0.02)	**0.002**												

Reference groups: Current mental health diagnoses (none), Professional role (clinical support role)

PTED = post-traumatic embitterment disorder; transgressions-others = Moral injury transgression by others; transgressions-self = Moral injury transgression by self; betrayal = Moral injury betrayal; CFC-I = Consideration of future consequences-Immediate; CFC-F = Consideration of future consequences-Future.

Significant p values are in bold.

[a] *N*=396

[b] *N*=394

Supplementary information: Confirmatory factor analysis for all study variables

Confirmatory factor analyses (CFA) for all instruments used in the present study were carried out with AMOS 26, and all estimations are based on maximum likelihood procedures. To assess model fit, we examined incremental fit indices of the comparative fit index (CFI) and Tucker-Lewis index (TLI) and the absolute fit indices of root-mean square error of approximation (RMSEA) and standardised root mean square residual (SRMR). In line with recommendations by Perry, Nicholls, Clough, and Crust[29] no arbitrary value was used as a cut-off. Instead, CFI and TLI close to 0.90 and SRMR and RMSEA close to zero (i.e., <0.06) were interpreted as representing adequate model fit.

Moral Injury Events Scale (MIES)

There is ongoing debate regarding model fit for the MIES, with a 'transgressions' (items 1-6) and 'betrayal' (i.e., items 7-9) two factor model proposed by Nash and colleagues[18], a 'transgression-other' (i.e., items 1,2,7,8, and 9) and 'transgression-self' two factor model (i.e., items 3-6) proposed by Richardson et al., and a three factor model proposed by Bryan et al.,[19](i.e., transgressions-others [items 1-2], transgressions-self [items 3-6] and betrayal [items 7-9]. We tested all three. Fit indices for Nash's initial two factor structure was suboptimal: $\chi^2(26) = 211.257$, CFI = .917, p<0.001, TLI = 0.886, SRMR = 0.087, RMSEA (90% CI) = 0.134 (0.117, 0.151). The two-factor structure proposed by Richardson showed an improvement in model fit: $\chi^2(26) = 104.894$, p<0.001, CFI = 0.965, TLI = 0.951, SRMR = 0.041, RMSEA (90% CI) = 0.09 (0.07, 0.11). However, the three-factor solution proposed by Bryan had superior model fit and was used in subsequent analyses: $\chi^2(24) = 36.217$, p=0.052, CFI = 0.995, TLI = 0.992, SRMR = 0.017, RMSEA (90% CI) = 0.036 (0.000, 0.058).

Post-traumatic embitterment scale (PTED)

The PTED scale is employed as a unidimensional scale and fit indices indicated that model fit was permissible despite some measurement error in the absolute fit indices, $\chi^2(152)$

= 617.373, p<0.001, CFI = 0.924, TLI = 0.915, SRMR = 0.044, RMSEA (90% CI) = 0.088 (0.080, 0.095).

Exposure to occupational stressors scale

For this study we developed a measure assessing exposure to occupational stressors. A unidimensional model in which all items loaded onto one latent factor presented a satisfactory model fit: $\chi^2(5) = 35.32$, p<0.001, CFI = 0.944, TLI = 0.934, SRMR = 0.059, RMSEA (90% CI) = 0.051 (0.036, 0.066). This is noteworthy given that this was a newly developed instrument.

Revised Life Orientation Test (LOT-R)

The LOT-R can be used as a unidimensional scale by reverse-scoring pessimism items to generate an overall optimism score. However, Glaesmer et al.,[21] argued for the use of a two-factor model. We tested both and found the unidimensional model fit to be unsatisfactory: $\chi^2(9) = 98.909$, p<0.001 CFI = 0.925, TLI = 0.875, SRMR = 0.055, RMSEA (90% CI) = 0.158 (0.131, 0.187). The two-factor model presented a significant improvement and was used in subsequent analyses: $\chi^2(8) = 23.268$, p=0.003 CFI = 0.987, TLI = 0.976, SRMR = 0.027, RMSEA (90% CI) = 0.069 (0.037, 0.103).

Rosenberg Self-esteem Scale

The Rosenberg self-esteem is primarily employed as a unidimensional scale with negatively worded items reverse scored. The unidimensional scale displayed some measurement error, primarily in the absolute fit indices: $\chi^2(35) = 203.48$, p<0.001, CFI = 0.920, TLI = 0.897, SRMR = 0.049, RMSEA (90% CI) = 0.110 (0.095, 0.125). However, all factor loadings were significant.

Brief Resilience Coping Scale

The unidimensional factor structure of the brief resilient coping scale demonstrated good model fit $\chi^2(2) = 3.243$ p=.198, CFI = .993, TLI = 0.980, SRMR = 0.021, RMSEA (90% CI) = 0.039 (0.000, 0.115).

Personal belief in a just world scale (personal BJW)

According to Lucas, Zhdanova and Alexander[24] the personal BJW is comprised of two factors, distributive justice (distributive justice; items 1-4) and procedural justice (procedural justice; items 5-8). First, we tested a unidimensional model in which all items loaded onto a single latent factor and found this model was poor, $\chi^2(20) = 802.839$, CFI = 0.723, TLI = 0.612, SRMR = 0.164, RMSEA (90% CI) = 0.313 (0.295, 0.332). The two-factor model displayed some measurement error as shown by the absolute fit indices but had much better fit; $\chi^2(19) = 144.221$, CFI = 0.956, TLI = 0.935, SRMR = 0.027, RMSEA (90% CI) = 0.129 (0.109, 0.149). The two sub-scales were used in subsequent analyses.

Consideration of future consequences-14 scale (CFC-14)

Given the on-going question regarding the model fit for the CFC scale[16] we examined a one-factor and a two-factor model. Model fit for a one-factor solution was less than ideal: $\chi^2(77)= 594.219$, $p < 0.001$; CFI = 0.749; TLI = 0.704; RMSEA = 0.130 (95% CI = 0.120, 0.140); SRMR = 0.105. Conversely, fit indices for the two-factor solution were acceptable and used in subsequent analyses: χ^2 (76)=179.026, $p < 0.001$; CFI = 0.950; TLI = 0.940; RMSEA = 0.058 (95% CI = 0.047, 0.069); SRMR = 0.059.

Social Desirability scale

The unidimensional factor structure of the social desirability scale demonstrated some measurement error in the comparative fit indices: $\chi^2(65) = 130.815$, p<0.001, CFI = 0.861, TLI = 0.833, SRMR = 0.051, RMSEA (90% CI) = 0.051 (0.038, 0.163) and item 13 had a relatively poor factor loading (i.e., 0.28). Despite this, all factor loadings did significantly load onto their latent factor.
