# Supplementary material for: Morally injurious events and post-traumatic embitterment disorder in UK health and social care professionals during COVID-19: a cross-sectional web survey
Source: BMJ Open. 2022 May 6;12(5):e054062. doi: 10.1136/bmjopen-2021-054062 (PMC9082726; doi:10.1136/bmjopen-2021-054062)
Supplement: Author's manuscript [file bmjopen-2021-054062.draft_revisions.pdf]

BMJ Open

BMJ Open is committed to open peer review. As part of this commitment we make the peer review history of every article we publish publicly available.

When an article is published we post the peer reviewers' comments and the authors' responses online. We also post the versions of the paper that were used during peer review. These are the versions that the peer review comments apply to.

The versions of the paper that follow are the versions that were submitted during the peer review process. They are not the versions of record or the final published versions. They should not be cited or distributed as the published version of this manuscript.

BMJ Open is an open access journal and the full, final, typeset and author-corrected version of record of the manuscript is available on our site with no access controls, subscription charges or pay-per-view fees (http://bmjopen.bmj.com).

If you have any questions on BMJ Open's open peer review process please email info.bmjopen@bmj.com

BMJ Open

Moral injury and Post-traumatic embitterment disorder in UK health and social care professionals during COVID-19: a cross sectional analysis

Journal:	*BMJ Open*
Manuscript ID	bmjopen-2021-054062
Article Type:	Original research
Date Submitted by the Author:	03-Jun-2021
Complete List of Authors:	Brennan, Chloe; University of Liverpool, Psychology<br>McKay, Michael; Royal College of Surgeons in Ireland<br>Cole, Jon; University of Liverpool
Keywords:	COVID-19, MENTAL HEALTH, Health policy < HEALTH SERVICES ADMINISTRATION & MANAGEMENT

SCHOLARONE™
Manuscripts

I, the Submitting Author has the right to grant and does grant on behalf of all authors of the Work (as defined in the below author licence), an exclusive licence and/or a non-exclusive licence for contributions from authors who are: i) UK Crown employees; ii) where BMJ has agreed a CC-BY licence shall apply, and/or iii) in accordance with the terms applicable for US Federal Government officers or employees acting as part of their official duties; on a worldwide, perpetual, irrevocable, royalty-free basis to BMJ Publishing Group Ltd ("BMJ") its licensees and where the relevant Journal is co-owned by BMJ to the co-owners of the Journal, to publish the Work in this journal and any other BMJ products and to exploit all rights, as set out in our licence.

The Submitting Author accepts and understands that any supply made under these terms is made by BMJ to the Submitting Author unless you are acting as an employee on behalf of your employer or a postgraduate student of an affiliated institution which is paying any applicable article publishing charge ("APC") for Open Access articles. Where the Submitting Author wishes to make the Work available on an Open Access basis (and intends to pay the relevant APC), the terms of reuse of such Open Access shall be governed by a Creative Commons licence – details of these licences and which Creative Commons licence will apply to this Work are set out in our licence referred to above.

Other than as permitted in any relevant BMJ Author's Self Archiving Policies, I confirm this Work has not been accepted for publication elsewhere, is not being considered for publication elsewhere and does not duplicate material already published. I confirm all authors consent to publication of this Work and authorise the granting of this licence.

For peer review only

Moral injury and Post-traumatic embitterment disorder in UK health and social care professionals during COVID-19: a cross sectional analysis

Chloe J. Brennan[1], Michael T. McKay[2], Jon C. Cole[1]

[1] Department of Psychology, University of Liverpool, United Kingdom

[2] Department of Psychology, Royal College of Surgeons in Ireland, Dublin, Ireland

For peer review only

Keywords: Mental health, Stress disorders, Post-Traumatic, Pandemics, Personality

Word count: 3833

[1] **Correspondence to**
Chloe Brennan
Eleanor Rathbone Building
Bedford Street South
Liverpool
L69 7ZT
Telephone: +44 (0)151 794 2000
hlcbrenn@liverpool.ac.uk

Abstract:

Objective

To estimate the prevalence and predictors of moral injury and post-traumatic embitterment disorder (PTED) in UK health and social care professionals during the COVID-19 pandemic.

Design

Cross-sectional study.

Setting

September-October 2020 in the United Kingdom. Online survey hosted on Qualtrics, and recruited through Prolific.

Participants

400 health and social care workers, aged 18 or above and living and working in the UK during the pandemic.

Main outcome measures

Moral injury was assessed using the Moral Injury Events Scale and PTED was assessed using the PTED self-rating scale. Potential predictors were measured using surveys of exposure to occupational stressors, optimism, self-esteem, resilient coping style, consideration of future consequences, and personal belief in a just world.

Results

19% of participants displayed clinical levels of PTED, and 73% experienced at least one COVID-related moral injury. Exposure to occupational stressors increased risk of experiencing PTED and moral injury, whereas personal belief in a procedurally just world was a protective mechanism.

Conclusions

For peer review only

Moral injury and PTED are being experienced by UK healthcare professionals, particularly in those exposed to work-related stressors.

Strengths and limitations:

- To our knowledge, this paper provides the first empirical evidence of the prevalence of both moral injury and PTED in UK health and social care workers during the COVID-19 pandemic.
- These findings extend research on frontline healthcare workers to health and social care workers from a diverse range of job roles. Sample characteristics are broadly similar to the wider NHS workforce, increasing generalisability.
- Use of validated measures of moral injury, PTED and personality traits, and inclusion of occupational stressors and demographics allows us to indicate which groups are at increased risk of psychological distress. This can inform healthcare policy.
- The cross-sectional design prevents analysis of causal relationships, and data was collected several months after the pandemic began so comparisons to pre-pandemic levels of PTED and moral injury are not possible.
- Survey data suffers from response and social desirability bias. However, social desirability was controlled for in analyses.

For peer review only - http://bmjopen.bmj.com/site/about/guidelines.xhtml

Introduction:

By January 2021 the rapid spread of the novel coronavirus disease 2019 (COVID-19) had claimed the lives of 100,000 people in the UK.[1] Healthcare workers (HCWs) have been under increased pressure to care for patients, adding to work-related stress that prior to the pandemic was at its highest level since 2014.[2] The early psychological impact of COVID-19 on HCWs is not equivocally experienced as globally, rates of depression (8.9-50.4%), anxiety (14.5-44.6%), post-traumatic stress disorder (PTSD; 8.3-88.4%), stress (2.2-93.8%) and burnout (21.8-46.3%) in HCWs vary widely across studies.[3,4] Research has focused predominantly on HCWs, but preliminary evidence into UK health and social care workers (HSCWs) during the pandemic indicates that nearly two thirds meet threshold for clinically significant disorders (PTSD = 22%, anxiety = 47%; depression = 47%).[5]

Health research in the wake of the COVID-19 pandemic has largely focussed on symptoms of anxiety, depression, PTSD and stress. However, it is arguable that an equally pressing health-related concern is the way in which HSCWs have been treated throughout the pandemic, and the associated psychological impact. Individuals have risked their lives during shortages in staff, personal protective equipment (PPE), and resources, which can lead to feelings of injustice and increased pressure to make morally challenging decisions.[6] Doctors surveyed in December 2020 reported feeling unable to provide the right care at the right time, with around 40% experiencing feelings of anger/irritability.[7] These experiences have the potential to violate healthcare staff's ethical beliefs, placing them at an increased risk of experiencing moral injury and post-traumatic embitterment disorder (PTED).

Moral injury refers to the inner conflict experienced when perpetrating or witnessing events that have violated one's moral values, and is linked to shame, guilt and poor mental health

For peer review only - http://bmjopen.bmj.com/site/about/guidelines.xhtml

outcomes.[8] For example, moral injury is often experienced by military personnel after witnessing intense human suffering that can shake their core ethical beliefs. PTED is a reactive disorder, triggered by an event that is considered to be unjust, and is accompanied by intrusive thoughts, mood impairment, social withdrawal, hostility and feelings of hopelessness.[9] Whilst PTED is considered to be a pathological disorder, neither moral injury nor PTED feature in diagnostic manuals, however, there is a growing recognition that a subset of treatment resistant patients may be suffering with these conditions.[8,9]

The British Medical Association has suggested that moral injury may become one of the most significant injuries for healthcare staff responding to the COVID-19 pandemic, and preliminary evidence in the US[10] and China[11] appears to support this claim. Initial data from NHS check, a cohort study of UK healthcare staff during the pandemic, indicates that moral injury is linked to increased rates of mental health symptoms.[12] However, there is a lack of studies in the UK investigating the prevalence of both moral injury and PTED in healthcare staff, despite their similarities. Additionally, despite an increased risk, not all staff members will develop PTED and/or moral injury as a result of their experiences during the pandemic. The literature suggests that individuals high in optimism, self-esteem, resilient coping, and belief in a just world, are at a reduced risk of experiencing PTED and/or moral injury,[8,13] and individuals who consider the future consequences of their behaviour have better health outcomes.[14] The current study reports on available baseline findings from a longitudinal study of UK health and social care workers that began in September-October 2020. The study aims to establish the prevalence of PTED and moral injury and identify potential risk and protective factors. Further waves of this study will investigate whether factors at baseline predict future PTED and/or moral injury and establish whether prevalence rates increase across time as the pandemic continues.

For peer review only - http://bmjopen.bmj.com/site/about/guidelines.xhtml

Methods:

Design:

This is a cross-sectional analysis of baseline data gathered after the first peak of COVID-19 in the UK (24th September – 6th October 2020). Ethical approval was granted by the Ethics Committee at The University of Liverpool.

Participants and procedure:

Participants were 400 HSCWs, aged 18-67, residing in the UK, and employed during the COVID-19 pandemic (see tables 1 and 2 for self-reported demographics). Participants were recruited via Prolific, an online crowdsourcing platform and received £2.50 for completing the survey (in line with platform guidelines). A further wave of data will take place at a 12-month follow up. Participants were directed to an online survey hosted on Qualtrics where they provided informed consent, responded to psychological measures in a randomised order, were debriefed, and redirected back to Prolific. The survey took approximately 15 minutes to complete. Power calculation conducted in G*Power found that 194 participants was required to find a small to moderate effect size with power 0.80 and α=0.05. To account for attrition rates of >50% in longitudinal studies, 400 participants were recruited.

Patient and Public involvement:

No patients or the public were involved in the design or conduct of this study.

Materials:

Participants self-reported their age, gender, ethnicity, professional role, years in current role, and any existing mental health diagnoses.

Moral injury was assessed using the Moral Injury Events Scale (MIES),[15] a nine-item scale measuring exposure to military events that violate moral beliefs. The scale was adapted to

For peer review only

reflect a healthcare sample in two ways. First, participants were asked to respond with regard to their experiences as a HSCW professional since the outbreak of COVID-19. Second, three items assessing betrayal by “leaders”, “fellow service members” and “others outside of the U.S. military” in the original scale were changed to “superiors” “fellow colleagues” and “others outside of my work organisation”. Ratings were made on a 6-point Likert scale, ranging from 1 (*strongly disagree*) to 6 (*strongly agree*). A recent factor analysis found three reliable subscales of the MIES; (1) transgressions by others (Transgression-Others; $\alpha = 0.79$), (2) transgressions by self (Transgressions-Self; $\alpha = 0.94$), and (3) betrayal (Betrayal; $a = 0.89$).[16] The mean scores across items in each factor were used in analyses.

PTED was assessed using the PTED Self-Rating Scale,[9] which is a 19-item scale assessing embitterment following negative life events. Participants were asked to respond with regard to working during the COVID-19 pandemic on a 5-point scale ranging from 0 (*not true at all*) to 4 (*extremely true*). The mean score across the 19 items was used in analyses relating to PTED. In line with recommendations, [9] a mean score of $\geq$2 was considered to be indicative of clinically relevant levels of PTED.

To measure exposure to occupational stressors, a five-item scale was developed for this study. Participants indicated on a 4-point scale (1 = *not at all*, 4 = *all the time*) how often they experienced: (1) exposure to COVID-19 in their job; (2) a lack of PPE and/or clear training; (3) having to make difficult decisions regarding resource allocation; (4) an inability to provide adequate care or save lives; and (5) separation from, or fear for loved ones due to working during the current pandemic. Items were summed, with higher scores reflecting higher exposure.

The Revised Life Orientation Test (LOT-R)[17] is a ten-item scale assessing generalised optimism. Responses (0 = *strongly disagree*, 4 = *strongly agree*) to four filler items were

For peer review only - http://bmjopen.bmj.com/site/about/guidelines.xhtml

removed. As a result of confirmatory factor analysis (see Supplementary materials) we employed a two-factor model where the sum of three items reflected higher optimism scores and the sum of three items reflected higher pessimism scores.

The Rosenberg (1965) Self-Esteem scale (R-SES)[18] asked participants to indicate their agreement to ten items using a 4-point Likert scale where 1 = *strongly disagree* and 4 = *strongly agree*. Five negatively worded items were recoded so that higher total scores reflected higher self-esteem.

The Brief Resilience Coping Scale (BRCS)[19] is a short scale measuring tendency to cope with stress adaptively. Four items were assessed on a 5-point Likert scale (1 = *does not describe at all*, 4 = *describes me very well*). Total scores range from 4-20, with high scores indicating resilient coping ability.

The Belief in a Just World Scale (BJW)[20] contains eight items measuring perceptions of fairness towards oneself (personal BJW) and eight items measuring perceptions of fairness towards others (general BJW). Personal BJW better predicts psychological well-being[18] so only this scale was used. These eight items were rated on a 7-point Likert scale from 1 (*strongly disagree*) to 7 (*strongly agree*). Four items measure distributive justice (personal BJW-DJ) and four items measure procedural justice (personal BJW-PJ).

The Consideration of Future Consequences-14 scale (CFC-14)[21] uses the sum of seven items to assess consideration of future consequences (CFC-F; $\alpha = 0.82$) and the sum of seven items to assess consideration of immediate consequences (CFC-I; $\alpha = 0.82$). Responses are given on a 7-point Likert ranging from 1 (*very unlike me*) to 7 (*very much like me*) and no items are reverse scored.

For peer review only - http://bmjopen.bmj.com/site/about/guidelines.xhtml

Social desirability was measured using the 13-item Marlowe-Crowne Social Desirability Scale.[22] Participants indicated either '*True*' (0) or '*False*' (1) and five items were reverse coded so higher scores reflected more socially desirable responses. Social desirability was controlled for in hierarchical regression analyses so no participants were excluded based on high scores.

Data analysis:

Preliminary analyses screened for outliers, missing data, univariate normality, and internal consistency, then confirmatory factor analyses were computed for each scale to determine factor structure and measurement error. Prevalence estimates for PTED and moral injury were calculated and Pearson's bivariate correlations examined the relatedness of these constructs. Next, we examined the impact of sociodemographic variables on PTED and MIES scores using independent samples t tests and a one-way ANOVA. Pearson's bivariate correlations with 2,000 bootstrapped samples examined the relationship between demographic, occupational, and personality variables on PTED and MIES scores. Four hierarchical regressions were then conducted for each dependent variable (PTED, Transgressions-Others, Transgressions-Self, and Betrayal) to assess the unique effects of demographics and social desirability (step 1), occupational stressors (step 2), and personality characteristics (step 3). MIES and PTED scores were entered as continuous variables, and categorical variables were dummy coded. For each of these analyses a p value of <0.05 was deemed significant and we used Fergusson's[23] recommendations for minimum practical ($d \geq 0.41$, $r \geq 0.20$, $\omega^2 \geq 0.04$,), medium ($d \geq 1.15$, $r \geq 0.50$, $\omega^2 \geq 0.25$,), and large effect sizes ($d \geq 2.70$, $r \geq 0.80$, $\omega^2 \geq 0.64$). Cohens f^2[24] was used to interpret the effect size of overall regressions, where $f^2 \geq 0.02$,

For peer review only - http://bmjopen.bmj.com/site/about/guidelines.xhtml

$f2 \geq 0.15$, and $f^2 \geq 0.35$ represented small, medium, and large effect sizes. Analyses were carried out using IBM SPSS v26, AMOS v26, or Stata v.16.

Results:

Preliminary results:

Of the 400 responses recorded, there were minimal missing data (<1%) for professional role and so pairwise deletion was used in univariate analyses and listwise deletion in multivariate regressions. Only 76.25% of the sample sufficiently reported their ethnicity so this variable was only included in univariate analyses to preserve data. Little's (1988)[25] test of Missing Completely at Random (MCAR) was not significant, $\chi(15) = 7.737$, $p = .934$ suggesting the data was missing completely at random. Based on results of confirmatory factor analysis for each scale, we used a three-factor solution for the MIES, and a two-factor solution for the LOT-R, the personal BJW and the CFC-14. All other scales used a unidimensional structure (see Supplementary materials). Internal consistency for all scales were acceptable ($\alpha > .70$), with the exception of BRCS, $\alpha = .63$. With the exception of 'years in current role', there were no problems with univariate normality (skewness ≤2 and kurtosis ≤3) however 13 univariate outliers (≥3.29) were identified across the following variables; years in role, BRCS, personal BJW-PJ, and CFC-F. Additionally, in ANOVA analysis Transgressions-Self scores exceeded kurtosis >3 for psychological professional role. Multivariate outliers were identified in three of the regressions, and homoscedasticity was violated in all regressions as identified by visual inspection. To deal with these violations, all analyses applied bias-corrected and accelerated (BCa) bootstrapping methods with 1000 to 2000 re-samples. Regression assumptions of linearity, independence of residuals, multi-collinearity, and multi-variate normality were met by

For peer review only - http://bmjopen.bmj.com/site/about/guidelines.xhtml

assessing partial regression plots, Durbin-Watson statistics, tolerance values greater than 0.1, and Q-Q- Plots.

Prevalence of MIES, PTED, and exposure to occupational stressors

The average score on the MIES was 2.55 (SD = 1.13) with highest scores observed on the Transgressions-Others subscale, followed by Betrayal, and Transgressions-Self (see table 1). In this sample, 72.3% [95% CI 67.6% to 76.6%] experienced at least one moral injury. On the PTED scale, using the clinical cut off score of ≥2[9] 19.25% [95% CI 15.5% to 23.5%] of the sample were embittered. For detailed prevalence estimates see supplementary materials. Prevalence of exposure to occupational stressors at "occasionally" or above on the Exposure scale revealed that 69.75% of participants reported being exposed to COVID-19 in their job, 57.5% were affected by a lack of PPE or COVID-19 training, 56.75% reported making difficult resource allocation decisions, 56.25% reporting feeling unable to provide adequate care or save patients they usually would, and 64% reported experiencing separation from, or fear for, loved ones due to working during COVID-19.

Univariate association between MIES scores, PTED scores and predictor variables

Table 1 displays mean scores, Pearson's bivariate correlations (two tailed) and internal consistency estimates of continuous study variables. Table 2 displays univariate tests of differences (two tailed) between categorical variables and PTED and MIES scores.

PTED was positively associated with all sub-scales of the MIES, and the largest association was with Betrayal (see table 1). According to Fergusson[23] these associations approached moderate effect sizes (r = .36 to .49) indicating that PTED and moral injury are distinct, but related psychological conditions.

For peer review only - http://bmjopen.bmj.com/site/about/guidelines.xhtml

PTED was significantly associated with older age and existing self-reported mental health diagnoses, Transgressions-Others was significantly associated with less years in current role, Betrayal was significantly associated with existing self-reported mental health diagnoses, and Transgressions-Self was significantly associated with professional role though no roles were significantly different from each other when applying Games-Howell correction for multiple comparisons. Exposure to occupational stressors and pessimism were significant risk factors for all dependent variables, CFC-I was a significant risk factor for PTED and Transgression-Others, and BRCS was a significant risk factor for Transgressions-Others. Significant protective factors for all dependent variables included R-SES and personal BJW-PJ, whereas personal BJW-DJ and optimism were specific protective factors for PTED and Betrayal. All dependent variables were significantly influenced by social desirability (see tables 1 and 2). All effect sizes were below the RPME or were small sized according to Fergusson[21].

For peer review only

For peer review only - http://bmjopen.bmj.com/site/about/guidelines.xhtml

Table 1.

Descriptive statistics, normality estimates, Cronbach's alpha estimates and Pearson's correlation coefficients (95% CI with 2,000 bootstrapped samples)

	Control variables			PTED and MIES				Situational and personality variables				
	1	2	3	4	5	6	7	8	9	10	11	12
1. Age	—											
2. Years in current role$_a$	.51** (.44 to .58)	—										
3. Social desirability	.13* (.03 to .23)	.15** (.06 to .24)	—									
4. PTED	-.13* (-.22 to -.04)	-.09 (-.19 to .01)	-.16** (-.25 to -.05)	—								
5. Transgressions -Other	-.08 (-.22 to -.04)	-.11* (-.19 to -.02)	-.11* (-.21 to -.01)	.36** (.26 to .45)	—							
6. Transgressions -Self	-.09 (-.18 to .01)	-.08 (.05 to -.17)	-.26** (-.34 to -.17)	.41** (.31 to .50)	.57** (.49 to .64)	—						
7. Betrayal	-.01 (-.11 to .10)	.00 (-.10 to .11)	-.17** (-.28 to -.06)	.49** (.41 to .57)	.61** (.54 to .68)	.53** (.44 to .60)	—					
8. Optimism	.06 (-.04 to .16)	.15** (.06 to 22)	.39** (.30 to .47)	-.18** (-.29 to -.08)	-.07 (-.17 to .03)	-.06 (-.16 to .04)	-.12* (-.23 to -.02)	—				

For peer review only - http://bmjopen.bmj.com/site/about/guidelines.xhtml

For peer review only

9. Pessimism	-.16** (-.25 to -.06)	-.13** (-.22 to -.04)	-.27** (-.36 to -.19)	.25** (.15 to .35)	.12* (.02 to .23)	.12* (.02 to .22)	.19** (.09 to .29)	-.67 (-.73 to -.60)	—			
10. BRCS	.04 (-.06 to .14)	.05 (-.04 to .16)	.30** (.21 to .39)	-.04 (-.14 to .06)	.11* (.01 to .20)	-.05 (-.15 to .05)	-.02 (-.12 to .08)	.49** (.41 to .56)	-.33** (-.43 to -.53)	—		
11. Personal BJW-DJ	-.02 (-.11 to .08)	.05 (-.07 to .14)	.19**(.09 to .29)	-.13** (-.23 to -.03)	-.07 (-.17 to .00)	-.08 (-.18 to .03)	-.16** (-.25 to -.06)	.47** (.36 to .56)	-.44** (-.53 to -.36)	.24** (.14 to .33)	—	
12. Personal BJW-PJ	-.09 (-.19 to .01)	-.03 (-.14 to .06)	.13** (.02 to .23)	-.31** (-.40 to -.21)	-.22** (-.32 to -.11)	-.22** (-.32 to -.11)	-.36** (-.44 to -.28)	.31** (.20 to .42)	-.32** (-.41 to -.21)	.19** (.07 to .30)	.54** (.45 to .61)	—
13. Occupational stressors	-.09 (-.18 to -.01)	.00 (-.09 to .10)	.04 (-.06 to .14)	.39**(.30 to .47)	.33** (.25 to .41)	.26** (.16 to .35)	.31** (.21 to .40)	.05 (-.05 to .15)	.04 (-.06 to .15)	.18** (.08 to .28)	.06 (.05 to .15)	-.06[illegible] .16 [illegible] .05)
14. CFC-I	-.01 (-.10 to .09)	.03 (-.07 to .14)	-.11* (-.20 to -.01)	.14** (.04 to .24)	.02 (-.09 to .13)	.16** (.06 to .27)	.07 (.05 to -.03)	.01 (-.10 to .10)	.13** (.03 to .22)	-.15** (-.25 to -.05)	.03 (-.08 to .13)	.01 to .[illegible]
15. CFC-F	-.10 (-.19 to .00)	-.01 (-.10 to .07)	.10* (-.01 to .20)	.06 (-.06 to .17)	.10 (-.02 to .20)	-.01 (-.12 to .10)	.02 (-.08 to .12)	.11* (-.01 to .22)	-.06 (-.17 to .06)	.24*** (.13 to .34)	.09 (-.02 to .19)	.03 to .[illegible]
16. R-SES	.16** (.05 to .25)	.12** (.06 to .24)	.47** (.38 to .55)	-.30** (-.39 to -.20)	-.11* (-.21 to -.01)	-.19** (-.28 to -.19)	-.17** (-.27 to -.08)	.68** (.60 to .74)	-.63** (-.69 to -.55)	.47*** (.37 to .55)	.43** (.34 to .52)	.27[illegible] (.16[illegible] .37[illegible]
Mean	36.84	4.5	6.93	1.12	2.98	2.17	2.77	6.60	5.82	14.6	18.75	20.[illegible]

For peer review only - http://bmjopen.bmj.com/site/about/guidelines.xhtml

SD	10.73	7	2.80	.90	1.48	1.23	1.38	2.72	2.82	2.4	4.7	4.7[illegible]
Skew	.73	2.10	-.07	.45	.16	.80	.35	-.46	.16	-.36	-.50	-.63
Kurtosis	-.22	5.14	-.50	-.75	-1.16	-.37	-.93	-.39	-.61	-.37	.94	-.13
Cronbach's α			.71	.96	.78	.93	.80	.83	.84	.62	.89	.95

*p<.05 **p<.01
PTED = Post-traumatic embitterment disorder; Transgression-Others = Moral injury transgression by others; Transgression-Self = Moral injury transgression by s
BRCS = Brief resilience coping scale; personal BJW-PJ = Personal just world beliefs-Procedural justice; personal BJW-DJ = Personal just world beliefs-Distribut
future consequences-Immediate; CFC-F = Consideration of future consequences-Future; R-SES = Self-esteem.
[a] Median/IQR reported due to non-normality

Table 2.
Socio-demographic characteristics of participants, and their association with PTED and the MIES subscales (95% CI with 2,000 bootstrapped samp

	N	%	PTED	*p*	Effect size	Transgressions-Others	*p*	Effect size	Transgressions-Self	*p*	Effect size	Betrayal	*p*
**Gender**$_a$													
Male	98	24.5%	1.17 (1.0)	.493	.08	3.07 (1.53)	.484	.08	2.21 (1.17)	.668	.05	2.86 (1.43)	.450
Female	302	75.5%	1.10 (.87)			2.95 (1.47)			2.15 (1.24)			2.74 (1.36)	
**Professional role**$_b$													
Allied health	58	14.5%	.96 (.92)	.215	.006	2.71 (1.44)	.374	.001	1.90 (1.05)	.021$_c$	.04	2.55 (1.41)	.589
Primary care practitioner	63	15.8%	1.04 (.91)			3.07 (1.45)			2.32 (1.27)			2.87 (1.42)	
Managerial	41	10.3%	.96 (.87)			2.66 (1.58)			1.84 (1.25)			2.60 (1.63)	

For peer review only - http://bmjopen.bmj.com/site/about/guidelines.xhtml

Health info/science	37	9.3%	1.38 (1)			3.18 (1.53)			2.40 (1.27)			2.89 (1.43)	
Psychological	27	6.8%	1.11 (.75)			2.83 (1.32)			1.79 (.98)			2.56 (1.08)	
Corporate	65	16.3%	1.07 (.78)			3.08 (1.48)			2.13 (1.09)			2.81 (1.38)	
Clinical support	105	26.3%	1.23 (.95)			3.14 (1.50)			2.43 (1.29)			2.92 (1.30)	
**Mental health**$_a$													
Existing mental health diagnoses	97	24.3%	1.52 (.96)	<.001$_d$	.64	3.20 (1.57)	.100	.20	2.31 (1.30)	.177	.15	3.10 (1.40)	.013$_e$
No existing mental health diagnoses	303	75.8%	.98 (.84)			2.91 1.45)			2.12 (1.20)			2.68 (1.36)	
**Ethnicity**$_a$													
White	252	63%	1.09 (.90)	.628	.07	2.93 (1.54)	.390	.13	2.08 (1.20)	.124	.23	2.77 (1.38)	.951
BAME	53	13.25%	1.15 (.93)			3.13 (1.38)			2.36 (1.16)			2.76 (1.40)	

Mean (SD) are reported. $_a$ Independent samples t-test reporting Cohen's *d* effect size, $_b$ One-way ANOVA reporting ω^2 effect size, $_c$ Welch's *F*(6, 138
2.579, *p*=.021, $_d$ Mean difference -.54 BCA 95% CI [-.74, -.34] t(398) = -5.33, p<.001, $_e$ Mean difference -.40 BCA 95% CI [-.72, -.07] t(293) = -2.5

[2] Non-parametric Mann Whitney-U and Kruskal Wallis-H tests were also conducted. There were no differences in findings.

For peer review only - http://bmjopen.bmj.com/site/about/guidelines.xhtml

Hierarchical multiple regressions.

All variables from Univariate (unadjusted) analyses were included in hierarchical regressions, with the exception of gender, which was not significantly associated with any outcome variables and ethnicity due to substantial missing data (see tables 3 and 4 for regressions).

The full model including demographics, occupational stressors, and personality characteristics explained between 38% and 22% of the variance in PTED and MIES subscales scores (adjusted R^2 = 35% to 18%). According to Cohen[24], this represents a large effect for PTED ($f^2 \geq 0.35$) and moderate effect sizes for all MIES regressions ($f^2 \geq 0.15$). Demographics significantly explained 3-10% of the variance in PTED, Transgressions-Self, and Transgressions-Others scores ($p<.05$), exposure to occupational stressors in step two significantly explained a further 8-15% of variances in all four regressions ($p<.001$), and personality characteristics in step three significantly explained a further 6-12% of variance in all four regressions ($p<.001$), with small to moderate effect sizes (f^2's of .05 to .21).

Significant risk factors for PTED included being in a clinical support role compared to an allied health or primary care practitioner, having an existing mental health diagnoses, exposure to occupational stressors, CFC-I, CFC-F, and personal BJW-DJ. Protective factors included personal BJW-PJ and R-SES.

Significant risk factors for Transgressions-Others included being in a clinical support role compared with allied health professionals, exposure to occupational stressors, and BRCS. The only protective factor was personal BJW-PJ.

For peer review only - http://bmjopen.bmj.com/site/about/guidelines.xhtml

Significant risk factors for Transgressions-Self included being in a clinical support role compared with an allied health and managerial role, exposure to occupational stressors, and CFC-I. Protective factors included personal BJW-PJ and R-SES.

Significant risk factors for Betrayal were being in a clinical support role compared to an allied health role and exposure to occupational stressors, whereas personal BJW-PJ was a significant protective factor. Social desirability was significantly associated with lower reporting of Betrayal and Transgressions-Self.

Overall, using Fergusson's[23] effect size guide, predictors with βs of practical significance (≥.20) included exposure to occupational stressors as risk factor and personal BJW-PJ as protective factor for PTED and all MIES sub-scales, R-SES as protective factor for PTED and social desirability resulted in lower Transgressions-Self and Betrayal scores. When running each hierarchical regression with only significant variables to improve model precision and parsimony, each full model explained approximately the same amount of variance in PTED and MIES scores (R^2 = 17% to 36%, see table 4). BRCS becomes non-significant in the Transgressions-Others model, managerial role and R-SES become non-significant in the Transgressions-Self model and allied role is no longer a significant predictor of Betrayal (see table 4).

For peer review only - http://bmjopen.bmj.com/site/about/guidelines.xhtml

Table 3.

Hierarchical multiple regression analyses predicting PTED and Transgressions-Others, Transgressions-Self and Betrayal scores with 95% bias corr samples) (N=394).

	PTED				Transgressions-Others				Transgressions-Self			
Predictors	β	95% CI	*p*	$R^2/\Delta R$	β	95% CI	*p*	$R^2/\Delta R$	β	95% CI	*p*	R
Step one - Controls				.102***				.038				.0
Step two – Occupational stressors				258/.155***				.161/.123***				.174
Step three – Personality				.376/.118***				.221/.060***				.240
Age	-.01	(-.01 to 01)	.789		.04	(-.01 to .02)	.555		.03	(-.01 to .02)	.587	
Existing mental health disorder	.12	(.07 to .46)	.006		-.01	(-.43 to .28)	.835		-.04	(-.39 to .18)	.512	
Social desirability	-.02	(-.03 to .02)	.729		-.08	(-.10 to .01)	.128		-.21	(-.14 to -.05)	.002	
Years in role	-.02	(-.02 to .01)	.747		-.10	(-.05 to .01)	.110		-.08	(-.04 to .01)	.185	
Allied	-.12	(-.60 to -.09)	.036		-.12	(-.93 to -.09)	.020		-.16	(-.87 to -.15)	.002	
Primary care practitioner	-.11	(-.54 to -.04)	.030		-.07	(-.72 to .14)	.201		-.09	(-.70 to .12)	.185	
Managerial	-.04	(-.37 to .15)	.398		-.11	(-1.12 to .04)	.077		-.13	(-.91 to -.03)	.028	
Health info/science	.06	(-.10 to .46)	.157		.03	(-.40 to .69)	.573		-.01	(-.47 to .43)	.939	
Psychological	.04	(-.12 to .50)	.307		-.02	(-.67 to .37)	.581		-.09	(-.92 to .04)	.061	
Corporate	-.02	(-.29 to .21)	.681		.00	(-.49 to .42)	.978		-.08	(-.58 to .09)	.142	

For peer review only - http://bmjopen.bmj.com/site/about/guidelines.xhtml

Occupational stressors	.38	(.08 to .12)	.006	.32	(.10 to .18)	.002	.26	(.06 to .13)	.002
Optimism	.03	(-.04 to .06)	.681	-.01	(-.09 to .08)	.876	.15	(-.01 to .13)	.073
Pessimism	-.01	(-.05 to .05)	.843	.01	(-.06 to .08)	.888	-.04	(-.08 to .05)	.644
BRCS	.06	(-.01 to .06)	.265	.15	(.03 to .16)	.014	.02	(-.05 to .06)	.746
Personal BJW DJ	.13	(.01 to .04)	.024	.08	(-.01 to .06)	.163	.08	(-.01 to .05)	.220
Personal BJW PJ	-.29	(-.07 to -.04)	.006	-.23	(-.11 to -.03)	.002	-.23	(-.09 to -.03)	.002
CFC I	.14	(.01 to .03)	.012	.03	(-.02 to .03)	.565	.12	(.01 to .04)	.037
CFC F	.11	(.01 to .03)	.036	.08	(-.01 to .04)	.130	.07	(-.01 to .03)	.262
R-SES	-.28	(-.07 to -.02)	.006	-.09	(-.07 to .01)	.207	-.19	(-.07 to -.01)	.016

*p<.05 ***p<.001
Reference groups: Existing mental health diagnosis (none), Professional role (clinical support role)
PTED = Post-traumatic embitterment disorder; Transgressions-Others = Moral injury transgression by others; Transgressions-Self = Moral injury tra
BRCS = Brief resilience coping scale; personal BJW-PJ = Personal just world beliefs-Procedural justice; personal BJW-DJ = Personal just world bel
consequences-Immediate; CFC-F = Consideration of future consequences-Future; R-SES = Self-esteem.

Table 4.
Hierarchical regression analyses using only significant predictors of PTED, Transgressions-Others, Transgressions-Self and Betrayal scores with 95% bias corrected and accelerated confidence intervals (2000 samples) (N=394 to 396).

Predictors	B	95% CI	*p*	$R^2/\Delta R$
Dependent variable: PTED$_a$				
Block one - controls				.076**
Block two – occupational stressors				.228/.152**
Block three – personality				.360/.132**
Existing mental health diagnosis	.12	(.07 to .43)	.005	
Allied	-.12	(-.54 to -.09)	.007	
Primary care practitioner	-.12	(-.50 to -.07)	.008	
Occupational stressors	.28	(.08 to .12)	.001	

For peer review only

Personal BJW-DJ	.13	(.01 to .05)	.011	
Personal BJW-PJ	-.27	(-.07 to -.03)	.001	
CFC-I	.14	(.01 to .03)	.004	
CFC-F	.12	(.01 to .03)	.006	
RSES	-.26	(-.05 to -.03)	.001	
Dependent variable: Transgressions-Others$_a$				
Step one – Controls				.006
Step two – Occupational stressors				.125/.118**
Step three – Personality				.171/.046**
Allied	-.11	(-.85 to -.04)	.024	
Occupational stressors	.32	(.10 to .17)	.001	
BRCS	.09	(-.01 to .12)	.062	
Personal BJW-PJ	-.21	(-.10 to -.04)	.001	
Dependent variable: Transgressions-Self$_b$				
Step one – Controls				.076**
Step two – Occupational stressors				.157/.081**
Step three – Personality				.200/.043**
Allied	-.11	(-.65 to -.10)	.007	
Managerial	-.07	(-.63 to .14)	.163	
Social desirability	-.20	(-.13 to -.04)	.001	
Occupational stressors	.27	(.06 to .13)	.001	
Personal BJW-PJ	-.16	(-.07 to -.02)	.001	
CFC-I	.11	(.01 to .03)	.021	
RSES	-.05	(-.03 to .01)	.371	
Dependent variable: Betrayal$_b$				
Step one – Controls				.034*
Step two – Occupational stressors				.135/.101**
Step three – Personality				.236/.101**
Allied	-.08	(-.65 to .10)	.098	
Social desirability	-.14	(-.11 to -.03)	.003	
Occupational stressors	.30	(.09 to .16)	.001	
Personal BJW-PJ	-.32	(-.12 to -.07)	.001	

*p<.05 ***p<.001

For peer review only - http://bmjopen.bmj.com/site/about/guidelines.xhtml

Reference groups: Existing mental health diagnosis (none), Professional role (clinical support role)
PTED = Post-traumatic embitterment disorder; Transgressions-Others = Moral injury transgression by others; Transgressions-Self = Moral injury transgression by self; Betrayal = Moral injury betrayal; BRCS = Brief resilience coping scale; personal BJW-PJ = Personal just world beliefs-Procedural justice; personal BJW-DJ = Personal just world beliefs-Distributive justice; CFC-I = Consideration of future consequences-Immediate; CFC-F = Consideration of future consequences-Future; R-SES = Self-esteem.

[a] *N*=396

[b] *N*=394

For peer review only

For peer review only - http://bmjopen.bmj.com/site/about/guidelines.xhtml

Discussion:

This is the first study to show that both PTED and moral injury were prevalent in UK HSCWs during September-October 2020 of the COVID-19 pandemic. Correlations show that PTED and moral injury are distinct but related conditions and are significantly associated with demographic, occupational, and personality variables. Hierarchical regressions show occupational stressors were a key risk factor, and personal belief in a procedurally just world, a key protective factor for both conditions. To a lesser extent, being in a clinical support role was a risk factor for both PTED, and moral injury. Beyond this, risk and protective factors have more selective effects. For example, in the final regression models, having an existing self-reported mental health diagnosis, CFC-I, CFC-F, and personal BJW-DJ were risk factors and R-SES a protective factor for PTED, CFC-I was a risk factor for Transgressions-Self and social desirability was associated with significantly lower reporting of Transgressions-Self and Betrayal, though effect sizes indicate these findings are less practically meaningful.

Our study has a number of strengths and limitations. This study is novel, providing the first preliminary evidence of both moral injury and PTED in UK HSCWs during the COVID-19 pandemic. The results are also generalisable. The survey extends research on HCWs by being open to all UK HSCWs on Prolific, and sample characteristics were broadly similar to the wider NHS workforce in terms of gender (female 76%, NHS = 77%) and ethnicity given that we had missing data (White = 63%, BAME = 13.25%, NHS White = 79.2% vs BAME = 20.7%). The sample included diverse job roles, and 25% of the sample self-reported existing mental health diagnoses which matches the general population. Most notable amongst the limitations is the correlational data, collected several months after the pandemic began, the fact that no comparisons to pre-pandemic levels of PTED and moral injury were possible, and that self-report

For peer review only - http://bmjopen.bmj.com/site/about/guidelines.xhtml

surveys suffer from response and social desirability bias. However, our study did control for social desirability bias.

Despite these limitations, our study provides empirical support to claims that moral injury is a likely consequence of acute exposure to the pandemic.[6] Prevalence in this sample is higher than that reported in US healthcare workers during COVID-19,[10] and is similar to those reported by active-duty US marines.[15] Prevalence of PTED in this sample is eight times higher than a general population sample,[9] but is lower than reported by UK NHS staff attending an occupational health centre pre-pandemic.[29] This indicates that healthcare staff experiencing PTED may be at a higher risk of requiring occupational health services. Further, despite stark similarities between the causes and manifestations of PTED and moral injury, this is the first empirical attempt to examine their similarities. Both conditions share the same key risk and protective factors, although regressions show occupational and personality variables only partially predict PTED and moral injury. This provides limited support to theoretical models highlighting the importance of personality variables.[8,13, 14]

These findings allow for a number of recommendations. First, some individuals may recover from moral injuries and feelings of embitterment on their own, but others will need psychological support and clinicians should be aware that these individuals are relatively treatment resistant and have different treatment needs.[8,9] More importantly though, there should be an emphasis not on the individual to 'recover' but on healthcare leaders to tackle systemic issues that are causing moral injuries and embitterment in the first place. In this study, staff across all levels of health and social care have reported feeling betrayed, morally violated and that they had been treated unjustly and unfairly during the pandemic. A proportion of these responses have been directly linked to the working environment (e.g., lack of PPE and

For peer review only - http://bmjopen.bmj.com/site/about/guidelines.xhtml

resources). Managers must use fair processes towards their staff, otherwise they risk a demoralised workforce, high turnover rates, and a culture that no one wants to work in. At a time when the UK needs a healthy workforce to respond to the pandemic and its aftermath, staff retention is a priority.

To strengthen prevalence estimates reported here, large scale cohort studies using randomly selected, representative samples are needed. Follow up data collection with these samples can establish whether prevalence estimates increase across time as the pandemic continues. Future studies should identify the cognitive, affective and behavioural manifestations of PTED and moral injury in healthcare professionals to inform treatment and treatment efficacy then needs to be established.

For peer review only

Contributor and guarantor information:

CB designed the project and data collection tools, collected the data, cleaned, analysed and interpreted the data and drafted and revised the paper. She is guarantor. MM analysed and interpreted the data and revised the paper. JC designed the project and data collection tools, interpreted the data, and revised the paper. All authors gave final approval for the publication of this manuscript. The corresponding author attests that all listed authors meet authorship criteria and that no others meeting the criteria have been omitted.

Copyright:

For peer review only - http://bmjopen.bmj.com/site/about/guidelines.xhtml

The Corresponding Author has the right to grant on behalf of all authors and does grant on behalf of all authors, an exclusive licence (or non-exclusive for government employees) on a worldwide basis to the BMJ Publishing Group Ltd to permit this article (if accepted) to be published in BMJ editions and any other BMJPGL products and sublicences such use and exploit all subsidiary rights, as set out in our licence.

Competing interest statement:

All authors have completed the Unified Competing Interest form (available on request from the corresponding author) and declare: the lead author had funding support from the Economic and Social Research Council and University of Liverpool COVID-19 strategic funding for the submitted work; no financial relationships with any organisations that might have an interest in the submitted work in the previous three years; no other relationships or activities that could appear to have influenced the submitted work.

Funding:

CB was supported by the Economic and Social Research Council UK [ES/P000665/1] and this work received COVID-19 strategic funding from the University of Liverpool.

Role of sponsor:

The study sponsors did not play a role in the study design, management, data collection, analyses, in the interpretation of the data or in the writing of the manuscript or the decision to submit for publication.

Transparency declaration:

The lead author affirms that this manuscript is an honest, accurate, and transparent account of the study being reported; that no important aspects of the study have been omitted; and that any discrepancies from the study as planned have been explained.

For peer review only - http://bmjopen.bmj.com/site/about/guidelines.xhtml

Ethical approval:

Ethical approval for the study was granted by the University of Liverpool (reference: 7862) and all participants provided informed consent.

Data availability statement:

Data from this study will be made available upon reasonable request by contacting the corresponding author at hlcbrenn@liverpool.ac.uk. Data will be made available after an embargoed period of 12 months to permit planned analyses of the longitudinal dataset and will be available for 10 years in line with ethical considerations.

Dissemination declaration:

Results from this study will be disseminated to participants that request this feedback and will be disseminated to relevant audiences through University of Liverpool communications.

For peer review only

References:

1. John Hopkins University (JHU). Mortality Analyses–Coronavirus Resource Centre. JHU, 2020 (https://coronavirus.jhu.edu/data/mortality).
2. NHS England. NHS staff survey. https://www.nhsstaffsurveyresults.com (Accessed 5 Feb 2020).

For peer review only - http://bmjopen.bmj.com/site/about/guidelines.xhtml

3. De Kock JH, Latham HA, Leslie SJ, Grindle M, Munoz S-A, Ellis L, et al. A rapid review of the impact of COVID-19 on the mental health of healthcare workers: implications for supporting psychological well-being. *BMC Public Health* 2021;21:104.

4. Major A, Hlubocky FJ. Mental health of health care workers during the COVID-19 pandemic and evidence-based frameworks for mitigation: A rapid review. *medRxiv* 2021.01.03.21249166 [Preprint]. January 04, 2021 [cited 2021 Feb 5] https://doi.org/10.1101/2021.01.03.21249166.

5. Greene T, Harju-Seppänen J, Adeniji M, Steel C, Grey N, Brewin CR et al. Predictors and rates of PTSD, depression and anxiety in UK frontline health and social care workers during COVID-19. European Journal of Psychotraumatology 2021;12. DOI: 10.1080/20008198.2021.1882781

6. Greenberg N, Docherty M, Gnanapragasam S, Wessely S. Managing mental health challenges faced by healthcare workers during covid-19 pandemic. *BMJ* 2020;368:m1211.

7. British Medical Association COVID-19 survey December 2020. https://www.bma.org.uk/media/3637/bma-covid-survey-results-all-doctors-dec-2020.pdf (Accessed 5 April 2021).

8. Litz BT, Stein N, Delaney E, Lebowitz L, Nash WP, Silva C, et al. Moral injury and moral repair in war veterans: A preliminary model and intervention strategy. *Clinical Psychology Review* 2009;29:695-706.

9. Linden M, Baumann K, Lieberei B, Rotter M. The post-traumatic embitterment disorder Self-Rating Scale (PTED Scale). Clinical Psychology & Psychotherapy 2009;16:139-47.

For peer review only - http://bmjopen.bmj.com/site/about/guidelines.xhtml

10. Hines SE, Chin KH, Levine AR, Wickwire EM. Initiation of a survey of healthcare worker distress and moral injury at the onset of the COVID-19 surge. *Am J Ind Med* 2020;63:830-3.

11. Zhizhong W, Koenig HG, Yan T, Jing W, Mu S, Hongyu L, et al. Psychometric properties of the moral injury symptom scale among Chinese health professionals during the COVID-19 pandemic. *BMC Psychiatry* 2020;20:556.

12. Lamb D, Gnanapragasam S, Greenberg N, Bhundia R, Carr E, Hotopf M et al. The psychosocial impact of COVID-19 pandemic on 4,378 UK healthcare workers and ancillary staff: initial baseline data from a cohort study collected during the first wave of the pandemic. *medRxiv* 2021.01.03.21249166 [Preprint]. January 22, 2021 [cited 2021 March 23]

13. Dodek A, Barnow S. Personality as risk and resilience in embitterment reactions. In: Linden M, Maercker A, editors. Embitterment: Societal, psychological, and clinical perspectives. Vienna: Springer Vienna; 2011. p. 83-100.

14. McKay MT, Perry JL, Percy A, Cole JC. Evidence for the reliability and validity, and some support for the practical utility of the two-factor Consideration of Future Consequences Scale-14. *Personality and Individual Differences* 2016;98:133-136. https://doi.org/10.1016/j.paid.2016.03.097

15. Nash WP, Marino Carper TL, Mills MA, Au T, Goldsmith A, Litz BT. Psychometric Evaluation of the Moral Injury Events Scale. Military Medicine 2013;178:646-52.

16. Bryan CJ, Bryan AO, Anestis MD, Anestis JC, Green BA, Etienne N, et al. Measuring moral injury: Psychometric properties of the moral injury events scale in two military samples. Assessment 2016;23:557-70.

For peer review only - http://bmjopen.bmj.com/site/about/guidelines.xhtml

17. Scheier MF, Carver CS, Bridges MW. Distinguishing optimism from neuroticism (and trait anxiety, self-mastery, and self-esteem): a reevaluation of the Life Orientation Test. J Pers Soc Psychol 1994;67:1063-78.

18. Rosenberg, M. (1965). Society and the adolescent self-image. Princeton, NJ: Princeton University Press

19. Sinclair VG, Wallston KA. The development and psychometric evaluation of the Brief Resilient Coping Scale. Assessment 2004;11:94-101.

20. Lucas T, Zhdanova L, Alexander S. Procedural and Distributive Justice Beliefs for Self and Others Assessment of a Four-Factor Individual Differences Model. Journal of Individual Differences 2011;32:14-25.

21. Strathman A, Gleicher F, Boninger DS, Edwards CS. The consideration of future consequences: weighing immediate and distant outcomes of behavior. J Pers Soc Psychol 1994;66:742.

22. Crowne DP, Marlowe D. A new scale of social desirability independent of psychopathology. Journal of consulting psychology 1960;24:349.

23. Ferguson CJ. An effect size primer: a guide for clinicians and researchers. Professional Psychology: Research and Practice 2016;5:532-538.

24. Cohen J. (1988). Statistical Power Analysis for the Behavioral Sciences (2nd ed.). Routledge. https://doi.org/10.4324/9780203771587

25. Little RJA. A test of missing completely at random for multivariate data with missing values. Journal of the American Statistical Association 1988;83:1198–1202

26. Perry J, Nicholls A, Clough P, Crust L. 2015. Assessing model fit: Caveats and recommendations for confirmatory factor analysis and exploratory structural equation

For peer review only - http://bmjopen.bmj.com/site/about/guidelines.xhtml

modelling. Measurement in Physical Education and Exercise Science 2015;19:12–21. https://doi.org/10.1080/1091367X.2014.952370

27. Richardson CB, Chesnut RP, Morgan, NR et al. Examining the Factor Structure of the Moral Injury Events Scale in a Veteran Sample. Military Medicine 2019;185:e75-e83. https://doi.org/10.1093/milmed/usz129

28. Glaesmer H, Rief W, Martin A, et al. Psychometric properties and population-based norms of the Life Orientation Test Revised (LOT-R). Br. J. Health. Psychol. 2012;17;432-445. doi: 10.1111/j.2044-8287.2011.02046.x

29. Sensky T, Salimu R, Ballard J, Pereira D. Associations of chronic embitterment among NHS staff. Occupational Medicine 2015;65;431-436. https://doi.org/10.1093/occmed/kqv089Practice, and Policy 2018;10:7-13. http://dx.doi.org/10.1037/tra0000291

For peer review only - http://bmjopen.bmj.com/site/about/guidelines.xhtml

Supplementary information: Confirmatory factor analysis for all study variables

Confirmatory factor analyses (CFA) for all instruments used in the present study were carried out with AMOS 26, and all estimations are based on maximum likelihood procedures. To assess model fit, we examined incremental fit indices of the comparative fit index (CFI) and Tucker-Lewis index (TLI) and the absolute fit indices of root-mean square error of approximation (RMSEA) and standardised root mean square residual (SRMR). In line with recommendations by Perry, Nicholls, Clough, and Crust[26] no arbitrary value was used as a cut-off. Instead, CFI and TLI close to .90 and SRMR and RMSEA close to zero (i.e., <.06) were interpreted as representing adequate model fit.

Moral Injury Events Scale (MIES)

There is ongoing debate regarding model fit for the MIES, with a 'transgressions' (items 1-6) and 'betrayal' (i.e., items 7-9) two factor model proposed by Nash and colleagues[15], a 'transgression-other' (i.e., items 1,2,7,8, and 9) and 'transgression-self' two factor model (i.e., items 3-6) proposed by Richardson et al.,[27] and a three factor model proposed by Bryan et al.,[16](i.e., transgressions-others [items 1-2], transgressions-self [items 3-6] and betrayal [items 7-9]. We tested all three. Fit indices for Nash's initial two factor structure was suboptimal: $\chi^2(26) = 211.257$, CFI = .917, p<.001, TLI = .886, SRMR = .087, RMSEA (90% CI) = .134 (.117, .151). The two-factor structure proposed by Richardson showed an improvement in model fit: $\chi^2(26) = 104.894$, p<.001, CFI = .965, TLI = .951, SRMR = .041, RMSEA (90% CI) = .09 (.07, .11). However, the three-factor solution proposed by Bryan had superior model fit and was used in subsequent analyses: $\chi^2(24) = 36.217$, p=.052, CFI = .995, TLI = .992, SRMR = .017, RMSEA (90% CI) = .036 (.000, .058).

Post-traumatic embitterment scale (PTED)

The PTED scale is employed as a unidimensional scale in studies and fit indices indicated that model fit was permissible despite some measurement error in the absolute fit

For peer review only

indices, $\chi^2(152) = 617.373$, p<.001, CFI = .924, TLI = .915, SRMR = .044, RMSEA (90% CI) = .088 (.080, .095).

Exposure to occupational stressors scale

For this study we developed a measure assessing exposure to occupational stressors. A unidimensional model in which all items loaded onto one latent factor presented a satisfactory model fit: $\chi^2(5) = 35.32$, p<.001, CFI = .944, TLI = .934, SRMR = .059, RMSEA (90% CI) = .051 (.036, .066). This is noteworthy given that this was a newly developed instrument.

Revised Life Orientation Test (LOT-R)

The LOT-R can be used as a unidimensional scale by reverse-scoring pessimism items to generate an overall optimism score. However, Glaesmer et al.[28] argued for the use of a two-factor model. We tested both and found the unidimensional model fit to be unsatisfactory: $\chi^2(9) = 98.909$, p<.001 CFI = .925, TLI = .875, SRMR = .055, RMSEA (90% CI) = .158 (.131, .187). The two-factor model presented a significant improvement and was used in subsequent analyses: $\chi^2(8) = 23.268$, p=.003 CFI = .987, TLI = .976, SRMR = .027, RMSEA (90% CI) = .069 (.037, .103).

Rosenberg Self-esteem Scale (R-SES)

The R-SES is primarily employed as a unidimensional scale with negatively worded items reverse scored. The unidimensional scale displayed some measurement error, primarily in the absolute fit indices: $\chi^2(35) = 203.48$, p<.001, CFI = .920, TLI = .897, SRMR = .049, RMSEA (90% CI) = .110 (.095, .125). However, all factor loadings were significant.

Brief Resilience Coping Scale (BRCS)

The unidimensional factor structure of the BRCS demonstrated good model fit $\chi^2(2) = 3.243$ p=.198, CFI = .993, TLI = .980, SRMR = .021, RMSEA (90% CI) = .039 (.000, .115).

Personal belief in a just world scale (personal BJW)

For peer review only

According to Lucas, Zhdanova and Alexander[20] the personal BJW is comprised of two factors, distributive justice (personal BJW-DJ; items 1-4) and procedural justice (personal. BJW-PJ; items 5-8). First, we tested a unidimensional model in which all items loaded onto a single latent factor and found this model was poor, $\chi^2(20) = 802.839$, CFI = .723, TLI = .612, SRMR = .164, RMSEA (90% CI) = .313 (.295, .332). The two-factor model displayed some measurement error as shown by the absolute fit indices but had much better fit; $\chi^2(19) = 144.221$, CFI = .956, TLI = .935, SRMR = .027, RMSEA (90% CI) = .129 (.109, .149). The two sub-scales were used in subsequent analyses.

Consideration of future consequences-14 scale (CFC-14)

Given the on-going question regarding the model fit for the CFC scale[14] we examined a one-factor and a two-factor model. Model fit for a one-factor solution was less than ideal: $\chi^2(77)= 594.219$, $p < .001$; CFI = .749; TLI = .704; RMSEA = .130 (95% CI = .120, .140); SRMR = .105. Conversely, fit indices for the two-factor solution were acceptable and used in subsequent analyses: χ^2 (76)=179.026, $p < .001$; CFI = .950; TLI = .940; RMSEA = .058 (95% CI = .047, .069); SRMR = .059.

Social Desirability scale

The unidimensional factor structure of the social desirability scale demonstrated some measurement error in the comparative fit indices: $\chi^2(65) = 130.815$, p<.001, CFI = .861, TLI = .833, SRMR = .051, RMSEA (90% CI) = .051 (.038, .163) and item 13 had a relatively poor factor loading (i.e., .28). Despite this, all factor loadings did significantly load onto their latent factor.

For peer review only

Supplementary information: Prevalence of item level endorsement on the MIES and PTED scale

Supplementary table 1.
Severity and frequency of endorsement of items on the moral injury events scale in U.K. health and social care workers

Item-level endorsement	**Raw frequency (N)**	**% (95% CI)**
Any item endorsed at ‘slightly agree’ or greater level	289	72.3% (67.6 to 76.6%)
Transgressions by Others:		
Either subscale item	213	53.3% (48.2 to 58.2%)
I saw things that were morally wrong	179	44.75% (39.8 to 49.8%)
I am troubled by having witnessed others immoral acts	167	41.75% (36.9 to 46.8%)
Transgressions by Self:		
Any subscale item	131	32.8% (28.2 to 37.6%
I acted in ways that violated by own moral code or values	76	19% (15.3 to 23.2%)
I am troubled by having acted in ways that violated by own moral code or values	94	23.5% (19.4 to 28%)
I violated by own morals by failing to do something that I felt I should have done	83	20.75% (16.9 to 25.1%)
I am troubled because I violated by own morals by failing to do something that I felt I should have done	78	19.5% (15.7 to 23.7%)
Betrayal		
Any subscale item	227	56.8% (51.7 to 61.7%)
I feel betrayed by superiors who I once trusted.	161	40.25% (35.4 to 45.2%)
I feel betrayed by fellow colleagues who I once trusted.	124	31% (26.5 to 35.8%)
I feel betrayed by others outside my work organisation who I once trusted.	158	39.5% (34.7 to 44.5%)

All MIES scales had a possible range of 1–6. For item-level endorsement, each MIES item was coded as endorsed if the participant responded either “slightly agree” “moderately agree” or “strongly agree”.

For peer review only

Supplementary table 2.
Severity and frequency of endorsement of items on the PTED scale in U.K. health and social care workers

**Cut off score**	**Raw frequency (N)**	**% (95% CI)**
1.6	121	30.3% (25.8 to 35%)
2	77	19.25% (15.5 to 23.5%)
2.5	30	7.5% (5.1 to 10.5%)
**Item-level endorsement**		
Any item endorsed by at "partially true" or greater level		
that hurt my feelings and caused considerable embitterment	142	35.5% (30.8 to 40.4%)
that led to a noticeable and persistent negative change in my mental well-being	167	41.75% (36.9 to 46.8%)
that I see as very unjust and unfair	170	42.5% (41.8 to 36.9%)
about which I have to think over and over again	167	41.75% (35.8 to 31%)
that causes me to be extremely upset when I am reminded of it	143	35.75% (31 to 40.7%)
that triggers me to harbour thoughts of revenge	44	11% (8.1 to 14.5%)
for which I blame and am angry with myself	87	21.75% (17.8 to 26.1%)
that led to the feeling that there is no sense to strive to make an effort	119	29.75% (25.3 to 34.5)
that makes me to frequently feel sullen or unhappy	151	37.75% (33 to 42.7%)
that impaired my overall physical well being	138	34.5% (29.8 to 39.4%)
that causes me to avoid certain places or persons so as to not be reminded of them	120	30% (25.5 to 34.8%)
that makes me feel helpless and disempowered	141	40.5% (35.7 to 45.5%)
that triggers feelings of satisfaction when I think that the responsible party having to experience a similar situation	76	19% (15.3 to 23.2%)
that led to a considerable decrease in my strength and drive	164	41% (36.1 to 46%)
that made that I am more easily irritated than before	176	44% (39.1 to 49%)
that makes that I must distract myself in order to experience a normal mood	160	40% (35.2 to 45%)
that made me unable to pursue occupational and/or family activities as before	176	44% (39.1 to 49%)
that caused me to draw back from friends and social activities	179	44.75% (39.8 to 49.8%)

For peer review only

which frequently evokes painful memories	118	29.5% (25.1 to 34.2%)

All PTED items had a possible range of 1–5. For item-level endorsement, each PTED item was coded as endorsed if the participant responded either "partially true" "very much true" or "extremely true".

For peer review only

STROBE Statement—Checklist of items that should be included in reports of ***cross-sectional studies***

	Item No	Recommendation	Page No
**Title and abstract**	1	(*a*) Indicate the study's design with a commonly used term in the title or the abstract	1
		(*b*) Provide in the abstract an informative and balanced summary of what was done and what was found	2
**Introduction**			
Background/rationale	2	Explain the scientific background and rationale for the investigation being reported	4, 5
Objectives	3	State specific objectives, including any prespecified hypotheses	5
**Methods**			
Study design	4	Present key elements of study design early in the paper	6
Setting	5	Describe the setting, locations, and relevant dates, including periods of recruitment, exposure, follow-up, and data collection	6
Participants	6	(*a*) Give the eligibility criteria, and the sources and methods of selection of participants	6
Variables	7	Clearly define all outcomes, exposures, predictors, potential confounders, and effect modifiers. Give diagnostic criteria, if applicable	6,7,8,9,10
Data sources/ measurement	8*	For each variable of interest, give sources of data and details of methods of assessment (measurement). Describe comparability of assessment methods if there is more than one group	6,7,8,9,10,15
Bias	9	Describe any efforts to address potential sources of bias	9
Study size	10	Explain how the study size was arrived at	6
Quantitative variables	11	Explain how quantitative variables were handled in the analyses. If applicable, describe which groupings were chosen and why	9,10
Statistical methods	12	(*a*) Describe all statistical methods, including those used to control for confounding	9,10
		(*b*) Describe any methods used to examine subgroups and interactions	9,10,12
		(*c*) Explain how missing data were addressed	10
		(*d*) If applicable, describe analytical methods taking account of sampling strategy	N/A
		(*e*) Describe any sensitivity analyses	N/A
**Results**			
Participants	13*	(a) Report numbers of individuals at each stage of study—eg numbers potentially eligible, examined for eligibility, confirmed eligible, included in the study, completing follow-up, and analysed	N/A
		(b) Give reasons for non-participation at each stage	N/A
		(c) Consider use of a flow diagram	N/A
Descriptive data	14*	(a) Give characteristics of study participants (eg demographic, clinical, social) and information on exposures and potential confounders	15,16
		(b) Indicate number of participants with missing data for each variable of interest	10
Outcome data	15*	Report numbers of outcome events or summary measures	

For peer review only - http://bmjopen.bmj.com/site/about/guidelines.xhtml

Main results	16	(*a*) Give unadjusted estimates and, if applicable, confounder-adjusted estimates and their precision (eg, 95% confidence interval). Make clear which confounders were adjusted for and why they were included	11-21
		(*b*) Report category boundaries when continuous variables were categorized	7
		(*c*) If relevant, consider translating estimates of relative risk into absolute risk for a meaningful time period	N/A
Other analyses	17	Report other analyses done—eg analyses of subgroups and interactions, and sensitivity analyses	N/A
**Discussion**			
Key results	18	Summarise key results with reference to study objectives	23
Limitations	19	Discuss limitations of the study, taking into account sources of potential bias or imprecision. Discuss both direction and magnitude of any potential bias	23,24
Interpretation	20	Give a cautious overall interpretation of results considering objectives, limitations, multiplicity of analyses, results from similar studies, and other relevant evidence	23,24
Generalisability	21	Discuss the generalisability (external validity) of the study results	23
**Other information**			
Funding	22	Give the source of funding and the role of the funders for the present study and, if applicable, for the original study on which the present article is based	26

*Give information separately for exposed and unexposed groups.

Note: An Explanation and Elaboration article discusses each checklist item and gives methodological background and published examples of transparent reporting. The STROBE checklist is best used in conjunction with this article (freely available on the Web sites of PLoS Medicine at http://www.plosmedicine.org/, Annals of Internal Medicine at http://www.annals.org/, and Epidemiology at http://www.epidem.com/). Information on the STROBE Initiative is available at www.strobe-statement.org.

For peer review only

Morally injurious events and post-traumatic embitterment disorder in UK health and social care professionals during COVID-19: a cross sectional web survey

Journal:	*BMJ Open*
Manuscript ID	bmjopen-2021-054062.R1
Article Type:	Original research
Date Submitted by the Author:	17-Mar-2022
Complete List of Authors:	Brennan, Chloe; University of Liverpool, Department of Psychology<br>McKay, Michael; Royal College of Surgeons in Ireland<br>Cole, Jon; University of Liverpool, Department of Psychology
**Primary Subject Heading**:	Mental health
Secondary Subject Heading:	Health policy, Health services research
Keywords:	COVID-19, MENTAL HEALTH, PSYCHIATRY, Health policy < HEALTH SERVICES ADMINISTRATION & MANAGEMENT

I, the Submitting Author has the right to grant and does grant on behalf of all authors of the Work (as defined in the below author licence), an exclusive licence and/or a non-exclusive licence for contributions from authors who are: i) UK Crown employees; ii) where BMJ has agreed a CC-BY licence shall apply, and/or iii) in accordance with the terms applicable for US Federal Government officers or employees acting as part of their official duties; on a worldwide, perpetual, irrevocable, royalty-free basis to BMJ Publishing Group Ltd ("BMJ") its licensees and where the relevant Journal is co-owned by BMJ to the co-owners of the Journal, to publish the Work in this journal and any other BMJ products and to exploit all rights, as set out in our licence.

The Submitting Author accepts and understands that any supply made under these terms is made by BMJ to the Submitting Author unless you are acting as an employee on behalf of your employer or a postgraduate student of an affiliated institution which is paying any applicable article publishing charge ("APC") for Open Access articles. Where the Submitting Author wishes to make the Work available on an Open Access basis (and intends to pay the relevant APC), the terms of reuse of such Open Access shall be governed by a Creative Commons licence – details of these licences and which Creative Commons licence will apply to this Work are set out in our licence referred to above.

Other than as permitted in any relevant BMJ Author's Self Archiving Policies, I confirm this Work has not been accepted for publication elsewhere, is not being considered for publication elsewhere and does not duplicate material already published. I confirm all authors consent to publication of this Work and authorise the granting of this licence.

For peer review only

Morally injurious events and post-traumatic embitterment disorder in UK health and social care professionals during COVID-19: a cross sectional web survey

Chloe J. Brennan[1], Michael T. McKay[2], Jon C. Cole[1]

[1] Department of Psychology, University of Liverpool, United Kingdom

[2] Department of Psychology, Royal College of Surgeons in Ireland, Dublin, Ireland

Keywords: Mental health, Stress disorders, Post-Traumatic, Pandemics, Personality

Word count: 4208

[1] **Correspondence to**
Chloe Brennan
Eleanor Rathbone Building
Bedford Street South
Liverpool
L69 7ZT
Telephone: +44 (0)151 794 2000
hlcbrenn@liverpool.ac.uk

For peer review only - http://bmjopen.bmj.com/site/about/guidelines.xhtml

Abstract:

Objective

To estimate the prevalence and predictors of morally injurious events and post-traumatic embitterment disorder (PTED) in United Kingdom (UK) health and social care professionals during the COVID-19 pandemic.

Design

Cross-sectional study.

Setting

September-October 2020 in the United Kingdom. Online survey hosted on Qualtrics, and recruited through Prolific.

Participants

400 health and social care workers, aged 18 or above and living and working in the UK during the pandemic.

Main outcome measures

Morally injurious events were assessed using the Moral Injury Events Scale and PTED was assessed using the PTED self-rating scale. Potential predictors were measured using surveys of exposure to occupational stressors, optimism, self-esteem, resilient coping style, consideration of future consequences, and personal belief in a just world.

Results

19% of participants displayed clinical levels of PTED, and 73% experienced at least one COVID-related morally injurious event. Exposure to occupational stressors increased risk of experiencing PTED and morally injurious events, whereas personal belief in a procedurally just world, which is the belief that they experienced fair processes, was a protective mechanism.

For peer review only

For peer review only - http://bmjopen.bmj.com/site/about/guidelines.xhtml

Conclusions

Morally injurious events and PTED are being experienced by UK healthcare professionals, particularly in those exposed to work-related stressors.

Strengths and limitations:

- This represents the first prevalence estimates of both morally injurious events and PTED in UK health and social care workers during COVID-19.
- Findings are generalisable to health and social care workers across diverse job roles rather than only 'frontline' healthcare staff.
- To inform healthcare policy, validated scales identified demographic, occupational, and personality traits associated with an increased risk of morally injurious events and PTED.
- The cross-sectional design prevents analysis of causal relationships, and comparisons to pre-pandemic levels of PTED and morally injurious events are not possible.
- Despite controlling for social desirability in analyses, survey data suffers from response and social desirability bias.

For peer review only

For peer review only - http://bmjopen.bmj.com/site/about/guidelines.xhtml

Introduction:

By January 2021 the rapid spread of the novel coronavirus disease 2019 (COVID-19) had claimed the lives of 100,000 people in the UK.[1] In the year prior to the pandemic, UK healthcare workers (HCWs) reported work-related stress at its highest level since 2014.[2] The early psychological impact of COVID-19 on HCWs is not equivocally experienced as globally, rates of depression (8.9-50.4%), anxiety (14.5-44.6%), post-traumatic stress disorder (PTSD; 8.3-88.4%), stress (2.2-93.8%) and burnout (21.8-46.3%) in HCWs vary widely across studies.[3,4] Research has focused predominantly on HCWs, but preliminary evidence into UK health and social care workers (HSCWs) during the pandemic indicates that nearly two thirds met thresholds for clinically significant disorders (PTSD = 22%, anxiety = 47%; depression = 47%).[5]

Health research in the wake of the COVID-19 pandemic has largely focussed on symptoms of anxiety, depression, PTSD, and stress. However, it is arguable that an equally pressing health-related concern is the way in which HSCWs feel they have been treated throughout the pandemic, and the associated psychological impact. Individuals have risked their lives during shortages in staff, limited availability of personal protective equipment (PPE) and other resources, which can lead to feelings of injustice and increased pressure to make morally challenging decisions.[6] Doctors surveyed in December 2020 reported feeling unable to provide the right care at the right time, with around 40% experiencing feelings of anger/irritability.[7] These experiences have the potential to violate healthcare staff's ethical beliefs, placing them at an increased risk of experiencing moral injury and post-traumatic embitterment disorder (PTED).

Witnessing or perpetrating events that violate one's moral values are called '*morally injurious events'* (MIEs), and moral injury encapsulates symptoms such as guilt, shame, and poor

For peer review only - http://bmjopen.bmj.com/site/about/guidelines.xhtml

mental health outcomes associated with these events.[8] Traditionally, moral injury has been studied in military personnel after witnessing the horrors of war. However, there is growing recognition that moral injury can occur in civilian healthcare professionals, and some argue that morally injured healthcare staff are being inaccurately diagnosed with burnout.[9] PTED is a reactive disorder, triggered by an unjust event, and is accompanied by intrusive thoughts, mood impairment, social withdrawal, hostility, and feelings of helplessness.[10,11] Theoretically, PTED and moral injury are very similar. Their aetiologies are the same as both develop following an event(s) that has breached an individual's core beliefs. After the event(s), symptoms span the emotional (i.e., moral emotions of anger and shame), cognitive (i.e., negative beliefs about oneself, others, and the world) and social (i.e., relational) domains.

The biggest distinction between PTED and moral injury is their approach to psychiatric diagnoses. Most PTED researchers agree that embitterment should be a separate psychiatric disorder in diagnostic manuals if it causes significant impairment to daily functioning for over 6 months.[10] In contrast, some moral injury researchers are hesitant to pathologize adaptive moral reactions to ethical issues and so moral injury is often considered a risk factor for other severe mental health issues.[6] Similarly, a useful conceptualisation of the PTED-moral injury relationship may be that exposure to MIEs can lead to PTED. Analysing this relationship can advance our understanding of moral trauma and provide a useful diagnosis for treatment resistant patients exhibiting these symptoms.

The British Medical Association has suggested that moral injury may become one of the most significant injuries for healthcare staff responding to the COVID-19 pandemic, and preliminary evidence in the United States (US)[12] and China[13] appears to support this claim. Initial data from National Health Service (NHS) check, a cohort study of UK healthcare staff

For peer review only - http://bmjopen.bmj.com/site/about/guidelines.xhtml

during the pandemic, indicates that MIEs are linked to increased rates of mental health symptoms.[14] However, there is a lack of studies in the UK investigating the prevalence of both moral injury and PTED in healthcare staff, despite their similarities. Additionally, despite an increased risk, not all staff members will develop PTED and/or moral injury as a result of their experiences during the pandemic. The literature suggests that individuals high in optimism, self-esteem, resilient coping, and belief in a just world, are at a reduced risk of experiencing PTED and/or moral injury,[8,15] and individuals who consider the future consequences of their behaviour have better health outcomes.[16] The current study reports on available baseline findings from a longitudinal study of UK health and social care workers that began in September-October 2020. The study aims to establish the prevalence of PTED and morally injurious events and identify potential risk and protective factors. Further waves of this study will investigate whether factors at baseline predict future PTED and/or morally injurious events, establish whether prevalence rates increase across time as the pandemic continues and investigate whether morally injurious events at baseline predict subsequent PTED.

Methods:

Design:

This is a cross-sectional analysis of baseline data gathered after the first peak of COVID-19 in the UK (24th September – 6th October 2020). Ethical approval was granted by the Ethics Committee at The University of Liverpool.

Participants and procedure:

Participants were 400 HSCWs, aged 18-67, residing in the UK, and employed during the COVID-19 pandemic. Job roles were categorised in line with careers on the NHS website[17]

For peer review only
For peer review only - http://bmjopen.bmj.com/site/about/guidelines.xhtml

(see supplementary Table 1 for self-reported demographics). Participants were recruited via Prolific, an online crowdsourcing platform and received £2.50 for completing the survey (in line with platform guidelines). A further wave of data will take place at a 12-month follow up. Participants were directed to an online survey hosted on Qualtrics where they provided informed consent, responded to psychological measures in a randomised order, were debriefed, and redirected back to Prolific. Due to participants potentially experiencing considerable levels of PTED and/or MIEs, appropriate support links and researchers' contact details were provided on the information and debrief sheets. The survey took approximately 15 minutes to complete. A power calculation conducted in G*Power found that 195 participants were required to find a small to moderate effect size of $f^2 = 0.12$ with power 0.80 and α=0.05. To account for attrition rates of >50% in longitudinal studies, 400 participants were recruited. Prior to study completion, 47 out of 447 participants dropped out, which is a response rate of 89.5%. To minimise non-response bias, study information was clearly communicated, and the survey was short.

Patient and Public involvement:

No patients or the public were involved in the design or conduct of this study.

Materials:

Participants self-reported their age, gender, ethnicity, professional role, years in current role, and any current mental health diagnoses.

Morally injurious events were assessed using the Moral Injury Events Scale (MIES),[18] a nine-item scale measuring exposure to military MIEs that violate moral beliefs. A higher score indicated more exposure to morally injurious events. The scale was adapted to reflect a health and social care sample in two ways. First, participants were asked to respond regarding their experiences as a HSCW professional since the outbreak of COVID-19. Second, three items

For peer review only - http://bmjopen.bmj.com/site/about/guidelines.xhtml

assessing betrayal by “leaders”, “fellow service members” and “others outside of the U.S. military” in the original scale were changed to “superiors” “fellow colleagues” and “others outside of my work organisation”. Ratings were made on a 6-point Likert scale, ranging from 1 (*strongly disagree*) to 6 (*strongly agree*). A recent factor analysis found three reliable subscales of the MIES; (1) transgressions by others (transgression-others; α = 0.79), (2) transgressions by self (transgressions-self; α = 0.94), and (3) betrayal (*a* = 0.89).[19] The mean scores across items in each subscale were used in analyses. For prevalence estimates, we calculated the percentage of participants who “slightly agreed”, “agreed”, or “strongly agreed” with each item of the MIES.

PTED was assessed using the PTED Self-Rating Scale,[11] which is a 19-item scale assessing embitterment following negative life events. Participants were asked to respond regarding working during the COVID-19 pandemic on a 5-point scale ranging from 0 (*not true at all*) to 4 (*extremely true*). The mean score across the 19 items was used in analyses relating to PTED. In line with recommendations, [11] a mean total score of ≥2 was considered to be indicative of clinically relevant levels of PTED.

To measure exposure to occupational stressors, a five-item scale was developed for this study. Participants indicated on a 4-point scale (1 = *not at all*, 4 = *all the time*) how often they experienced: (1) exposure to COVID-19 in their job; (2) a lack of PPE and/or clear training; (3) having to make difficult decisions regarding resource allocation; (4) an inability to provide adequate care or save lives; and (5) separation from, or fear for, loved ones due to working during the current pandemic. Items were summed, with higher scores reflecting higher exposure.

The Revised Life Orientation Test (LOT-R)[20] is a ten-item scale assessing generalised optimism. Responses (0 = *strongly disagree*, 4 = *strongly agree*) to four filler items were removed. As a result of confirmatory factor analysis (see Supplementary materials) we employed

For peer review only

For peer review only - http://bmjopen.bmj.com/site/about/guidelines.xhtml

a two-factor model suggested by Glaesmer et al.,[21] where the sum of three items reflected higher optimism scores and the sum of three items reflected higher pessimism scores.

The Rosenberg (1965) Self-Esteem Scale[22] asked participants to indicate their agreement to ten items using a 4-point Likert scale where 1 = *strongly disagree* and 4 = *strongly agree*. Five negatively worded items were recoded so that higher total scores reflected higher self-esteem.

The Brief Resilience Coping Scale[23] is a short scale measuring tendency to cope with stress adaptively. Four items were assessed on a 5-point Likert scale (1 = *does not describe at all*, 4 = *describes me very well*). Total scores range from 4-20, with high scores indicating resilient coping ability.

The Belief in a Just World Scale (BJW)[24] contains eight items measuring perceptions of fairness towards oneself (personal BJW) and eight items measuring perceptions of fairness towards others (general BJW). Personal BJW better predicts psychological well-being so only this scale was used. These eight items were rated on a 7-point Likert scale from 1 (*strongly disagree*) to 7 (*strongly agree*). Four items measure distributive justice and four items measure procedural justice.

The Consideration of Future Consequences-14 Scale (CFC-14)[25] uses the sum of seven items to assess consideration of future consequences (CFC-F; $\alpha = 0.82$) and the sum of seven items to assess consideration of immediate consequences (CFC-I; $\alpha = 0.82$). Responses are given on a 7-point Likert ranging from 1 (*very unlike me*) to 7 (*very much like me*) and no items are reverse scored.

Social desirability was measured using the 13-item Marlowe-Crowne Social Desirability Scale.[26] Participants indicated either ‘*True*’ (0) or ‘*False*’ (1) and five items were reverse

For peer review only - http://bmjopen.bmj.com/site/about/guidelines.xhtml

coded so higher scores reflected more socially desirable responses. Social desirability was controlled for in hierarchical regression analyses so no participants were excluded based on high scores.

Data analysis:

Of the 400 responses recorded, there was <1% missing data for social desirability and professional role, and 23.8% missing data for ethnicity, which was Missing Completely at Random (MCAR), $\chi(15) = 7.737$, p= 0.934[27]. Pairwise deletion was used in univariate analyses, and listwise deletion in multivariate analyses. Next, based on confirmatory factor analyses (CFA), we employed a three-factor solution for the MIES, and a two-factor solution for the LOT-R, the personal BJW and the CFC-14. All other scales used a unidimensional structure (see Supplementary materials). Prevalence estimates for PTED and MIES were calculated followed by Pearson's bivariate correlations to examine the relatedness of these constructs, and their relationship to predictor variables. Independent samples t-tests and a one-way ANOVA examined the impact of sociodemographic variables on PTED and MIES scores. Four hierarchical linear regressions were then conducted for each dependent variable (PTED, transgressions-others, transgressions-self, and betrayal) to assess the unique effects of demographics and social desirability (step 1), occupational stressors (step 2), and personality characteristics (step 3). Bias-corrected and accelerated (BCa) bootstrapping methods were applied to all analyses to deal with violations to normality, outliers and homoscedacity of variance, and all other assumptions were met. For all analyses $p < 0.05$ was deemed significant and we used Ferguson's[28] recommendations for minimum practical ($d \geq 0.41$, $r \geq 0.20$, $\omega^2 \geq 0.04$,), medium ($d \geq 1.15$, $r \geq 0.50$, $\omega^2 \geq 0.25$,), and large effect sizes ($d \geq 2.70$, $r \geq 0.80$, $\omega^2 \geq 0.64$). Cohen's f^2[29] was used to interpret the effect size of overall regressions, where $f^2 \geq 0.02$, $f^2 \geq 0.15$, and $f^2 \geq 0.35$ represented

For peer review only - http://bmjopen.bmj.com/site/about/guidelines.xhtml

small, medium, and large effect sizes. Recommendations by Perry et al.,[30] were used to interpret CFA where incremental fit indices close to 0.90 and absolute fit indices close to zero (i.e., <0.06) represented adequate model fit. Analyses were carried out using IBM SPSS v26, AMOS v26, or R v4.1.0.

Results:

Prevalence of MIES, PTED, and exposure to occupational stressors

In this sample, 72.3% [95% CI 67.6% to 76.6%] experienced at least one morally injurious event. On the PTED scale, using the clinical cut off score of ≥2[11] 19.3% [95% CI 15.5% to 23.5%] of the sample were embittered (see Table 1). For item level prevalence estimates see supplementary Table 2. The average score on the MIES was 2.55 (SD = 1.13) with highest scores observed on the transgressions-others subscale, followed by betrayal, and transgressions-self (see Table 2). Prevalence of exposure to occupational stressors at "occasionally" or above on the Exposure scale revealed that 69.8% of participants reported being exposed to COVID-19 in their job, 57.5% were affected by a lack of PPE or COVID-19 training, 56.8% reported making difficult resource allocation decisions, 56.3% reporting feeling unable to provide adequate care or save patients they usually would, and 64% reported experiencing separation from, or fear for, loved ones due to working during the pandemic.

Univariate association between MIES scores, PTED scores, and predictor variables

Table 2 displays mean scores, and internal consistency estimates, and Figure 1 displays a Pearson's correlation heatmap of continuous study variables. Supplementary Table 1 displays univariate tests of differences between categorical demographic variables and PTED and MIES scores.

For peer review only - http://bmjopen.bmj.com/site/about/guidelines.xhtml

PTED was positively associated with all sub-scales of the MIES, and the largest association was with betrayal (*p's*<0.001, see Figure 1). These associations approached moderate effect sizes ($r = 0.36$ to 0.49) indicating that PTED and MIEs are distinct, but related psychological constructs.

PTED was associated with older age ($p = 0.011$) and current self-reported mental health diagnoses ($p<0.001$), transgressions-others was associated with fewer years in current role ($p = 0.033$), betrayal was associated with current self-reported mental health diagnoses ($p = 0.013$), and transgressions-self was associated with professional role ($p = 0.021$) though no roles were significantly different from each other when applying Games-Howell correction for multiple comparisons. Risk factors for all dependent variables included exposure to occupational stressors (*p's*<0.001) and pessimism ($p<0.001$ with PTED and betrayal, $p = 0.014$ with transgressions-others and $p = 0.015$ with transgressions-self). CFC-I was a risk factor for PTED ($p = 0.005$) and transgressions-self ($p = 0.001$), and resilient coping style was a risk factor for transgressions-others ($p = 0.035$). Protective factors for all dependent variables included self-esteem ($p<0.001$ for associations with PTED, transgressions-self, and betrayal, and $p = 0.027$ with transgressions-others) and procedural justice (*p's*<0.001). Distributive justice and optimism were protective factors of PTED (distributive justice $p = 0.009$, optimism $p<.001$) and of betrayal (distributive justice $p = 0.002$, optimism $p = 0.015$). All dependent variables were influenced by social desirability (*p's* from <0.001 to 0.032, see Figure 1 and Supplementary table 1). All effect sizes were below the RPME or were small sized.

For peer review only - http://bmjopen.bmj.com/site/about/guidelines.xhtml

Table 1.

PTED prevalence, and frequency of endorsement of items on the MIES in UK health and social care workers

**PTED scale**		
**Cut off score**	**Raw frequency (N)**	**% (95% CI)**
1.6	121	30.3% (25.8 to 35%)
2	77	19.3% (15.5 to 23.5%)
2.5	30	7.5% (5.1 to 10.5%)
**MIES scale**		
Any scale item	289	72.3% (67.6 to 76.6%)
Transgressions by others subscale:	213	53.3% (48.2 to 58.2%)
Transgressions by self subscale	131	32.8% (28.2 to 37.6%
Betrayal subscale	227	56.8% (51.7 to 61.7%)

PTED prevalence was calculated by percentage scoring above a mean total score of 1.6, 2 and 2.5. Each MIES item was coded as endorsed if the participant responded either "slightly agree" "moderately agree" or "strongly agree". Subscale prevalence was calculated by endorsement of any item within that subscale.

Table 2.

Descriptive statistics, normality estimates and Cronbach's alpha estimates

	**Mean(±SD)**	**Skew**	**Kurtosis**	**Cronbach's α**
Age	36.84(±10.73)	0.73	-0.22	
Years$_a$	4.50(±7.0)	2.10	5.14	
Social Desirability	6.93(±2.80)	-0.07	-0.50	0.71
PTED	1.12(±0.90)	0.45	-0.75	0.96
Transgressions-others	2.98(±1.48)	0.16	-1.16	0.78
Transgressions-self	2.17(±1.22)	0.80	-0.37	0.93

For peer review only - http://bmjopen.bmj.com/site/about/guidelines.xhtml

Betrayal	2.77(±1.38)	0.35	-0.93	0.80
Optimism	6.60(±2.72)	-0.46	-0.39	0.83
Pessimism	5.82(±2.82)	0.16	-0.61	0.84
Resilient coping style	14.60(±2.40)	-0.50	0.94	0.62
Distributive justice	18.75(±4.70)	-0.63	-0.13	0.89
Procedural justice	20.33(±4.77)	-0.98	0.84	0.95
Occupational stressors	9.91(±3.44)	0.71	-0.06	0.76
CFC-I	23.47(±7.68)	0.19	-0.61	0.85
CFC-F	34.58(±6.85)	-0.50	0.41	0.84
Self-esteem	28.18(±5.80)	-0.25	-0.21	0.91

[a] Median/IQR reported due to non-normality.

PTED = post-traumatic embitterment disorder; transgressions-others = Moral injury transgression by others; transgressions-self = Moral injury transgression by self; betrayal = Moral injury betrayal; CFC-I = Consideration of future consequences-Immediate; CFC-F = Consideration of future consequences-Future.

For peer review only

Hierarchical multiple regressions.

All variables from Univariate (unadjusted) analyses were included in hierarchical regressions, except for gender and ethnicity, which were not significantly associated with any outcome variables (see Table 3 for regression model summary and supplementary Tables 3 and 4 for regression coefficients).

The full model including demographics, occupational stressors, and personality characteristics explained between 38% and 22% of the variance in PTED and MIES subscales scores (adjusted R^2 = 35% to 18%). This represents a large effect for PTED ($f^2 \geq 0.35$) and moderate effect sizes for all MIES regressions ($f^2 \geq 0.15$). Demographics significantly explained 10% of the variance in PTED, transgressions-self, and transgressions-others scores ($p<0.05$), exposure to occupational stressors in step two significantly explained a further 8-16% of variances in all four regressions ($p<0.001$), and personality characteristics in step three significantly explained a further 6-12% of variance in all four regressions ($p<0.001$), with small to moderate effect sizes (f^2's of 0.05 to 0.21).

Statistically significant risk factors for PTED included being in a clinical support role compared to an allied health (p = 0.036) or primary care practitioner (p = 0.030), having current mental health diagnoses (p = 0.006), exposure to occupational stressors (p = 0.006), CFC-I (p = 0.012), CFC-F (p = 0.036), and distributive justice (p = 0.024). Statistically significant protective factors included belief in procedural justice and self-esteem (*p's* = 0.006).

Statistically significant risk factors for transgressions-others included being in a clinical support role compared with allied health professionals (p = 0.020), exposure to occupational stressors (p = 0.002), and resilient coping style (p = 0.014). The only statistically significant protective factor was belief in procedural justice (p = 0.002).

For peer review only - http://bmjopen.bmj.com/site/about/guidelines.xhtml

Statistically significant risk factors for transgressions-self included being in a clinical support role compared with an allied health (p = 0.002) and managerial role (p = 0.028), exposure to occupational stressors (p = 0.002), and CFC-I (p = 0.037). Statistically significant protective factors included belief in procedural justice (p = 0.002) and self-esteem (p = 0.016).

Statistically significant risk factors for betrayal were being in a clinical support role compared to an allied health role (p = 0.036) and exposure to occupational stressors (p = 0.006), whereas belief in procedural justice was a statistically significant protective factor (p = 0.006). Social desirability was significantly associated with lower reporting of betrayal (p = 0.012) and transgressions-self (p = 0.002).

Overall, predictors with βs of practical significance (≥0.20) included exposure to occupational stressors as a risk factor and belief in procedural justice as a protective factor for PTED and all MIES sub-scales, self-esteem as a protective factor for PTED, and social desirability resulted in lower transgressions-self scores (see supplementary Table 3). When running each hierarchical regression with only significant variables to improve model precision and parsimony, each full model explained approximately the same amount of variance in PTED and MIES scores (R^2 = 19% to 37%, see Table 3). Managerial role (p = 0.064) and self-esteem (p = 0.427) become non-significant in the transgressions-self model (see supplementary Table 4).

For peer review only - http://bmjopen.bmj.com/site/about/guidelines.xhtml

Table 3.

Model summary of hierarchical regression analyses using all possible predictors and only significant predictors of PTED, transgressions-others, transgressions-self, and betrayal scores with 95% bias corrected and accelerated confidence intervals (2000 samples) (N=394 to 396).

Model including **all possible predictors**				
	PTED $_a$	Transgressions-others $_a$	Transgressions-self $_a$	Betrayal $_a$
Step one – Controls	0.10***	0.04	0.10***	0.05*
Step two – Occupational stressors	0.26/0.16***	0.16/0.12***	0.17/0.08***	0.15/0.10***
Step three – Personality	0.38/0.12***	0.22/0.06***	0.24/0.07***	0.26/0.10***

Model including **only significant predictors** from the previous model				
	PTED $_a$	Transgressions-others $_a$	Transgressions-self $_b$	Betrayal $_b$
	$R^2/\Delta R$	$R^2/\Delta R$	$R^2/\Delta R$	$R^2/\Delta R$
Step one – Controls	0.08***	0.02	0.10***	0.04*
Step two – Occupational stressors	0.24/0.16***	0.13/0.14***	0.17/0.08***	0.14/0.10***
Step three – Personality	0.37/0.13***	0.19/0.04***	0.21/0.04***	0.24/0.10***

*p<0.05, **p<0.01, ***p<0.001

PTED = post-traumatic embitterment disorder; transgressions-others = Moral injury transgression by others; transgressions-self = Moral injury transgression by self; betrayal = Moral injury betrayal.

$_a$ *N*=396

$_b$ *N*=394

For peer review only - http://bmjopen.bmj.com/site/about/guidelines.xhtml

Discussion:

This is the first study to show that both PTED and morally injurious events were prevalent in UK HSCWs during September-October 2020 of the COVID-19 pandemic. Correlations show that PTED and morally injurious events are distinct but related constructs and are significantly associated with demographic, occupational, and personality variables. Hierarchical regressions show that occupational stressors were a key risk factor, and personal belief in a procedurally just world, a key protective factor for both conditions. To a lesser extent, being in a clinical support role was a risk factor for both PTED, and exposure to morally injurious events. Beyond this, risk and protective factors have more selective effects. For example, in the final regression models, risk factors for PTED included having current self-reported mental health diagnoses, higher consideration of the immediate and future consequences of actions, and higher levels of personal belief in distributive justice. Self-esteem was a resiliency factor. Higher consideration of the immediate consequences of actions was a risk factor for transgressions-self, and higher levels of resilient coping style was a risk factor for transgressions-others. Social desirability was associated with significantly lower reporting of transgressions-self and betrayal, though effect sizes indicate these selective effects were less practically meaningful.

Our study has several strengths and limitations. This study is novel, providing the first preliminary evidence of both morally injurious events and PTED in UK HSCWs during the COVID-19 pandemic. The results are also generalisable. The survey extends research on HCWs by being open to all UK HSCWs on Prolific, and sample characteristics were broadly similar to the wider NHS workforce in terms of gender (female 76%, NHS = 77%) and ethnicity given that we had missing data (White = 63%, Ethnic minorities = 13.3%, NHS White = 79.2% vs Ethnic

For peer review only - http://bmjopen.bmj.com/site/about/guidelines.xhtml

minorities = 20.7%). The sample included diverse job roles, and 25% of the sample self-reported current mental health diagnoses which matches the general population. The most notable limitation is that cross-sectional baseline data were analysed, and only future analyses from our longitudinal study can establish causal relationships. Data were also collected several months after the pandemic began, no comparisons to pre-pandemic levels of PTED and MIEs were possible, and despite controlling for social desirability, surveys suffer from response and social desirability bias.

Despite these limitations, our study supports early findings that healthcare staff believe themselves to have been exposed to MIEs during the pandemic.[14] Prevalence in this sample is higher than that reported in US HCWs during COVID-19,[12] and is similar to those reported by active-duty US marines.[18] Prevalence of PTED in this sample is eight times higher than a general population sample,[11] but is lower than reported by UK NHS staff attending an occupational health centre pre-pandemic.[31] This indicates that healthcare staff experiencing PTED may be at a higher risk of requiring occupational health services. Further, despite similarities in aetiology and symptomology, studies have rarely examined the overlap between PTED and moral injury. Moderate correlations provide early support to our argument that MIEs can trigger PTED in some individuals and future longitudinal analyses will investigate this model.

Importantly, MIEs and PTED shared the same key risk and protective factors. Experiencing MIEs and PTED was more likely in clinical support workers and those experiencing occupational stressors. Exposure to occupational stressors likely increases the frequency of MIEs that can cause embitterment. Personal belief in procedural justice, which is the belief that you experience fair processes protected against exposure to MIEs and PTED.

For peer review only - http://bmjopen.bmj.com/site/about/guidelines.xhtml

Whilst this belief is generally seen as a disposition that can protect against the negative impact of injustices, it is equally possible that belief in a just world can be modified by exposure to injustices, leading to PTED.

Other vulnerability and resiliency factors vary. Having current mental health diagnoses increased the risk of PTED, and mental health conditions are often comorbid. Considering immediate and future consequences of actions increased risk of PTED, and this may be explained by feelings of helplessness when thinking about the unjust life event, as noted in diagnostic criterion.[10, 11] Belief in distributive justice, which is the belief that you experience fair outcomes increased risk of PTED in contrast to findings that procedural justice was a resiliency factor. Surprisingly, resilient coping style increased exposure to transgressions by others, despite findings that resilient coping tendencies are linked with better psychological well-being.[23] It is possible that people feel more comfortable committing moral transgressions around individuals that they consider to be resilient. Self-esteem increased resilience to PTED, and reliably buffers against poor mental health in the literature.[22] Combined, these findings indicate several vulnerability and resiliency factors that either increase or decrease the risk of exposure to MIEs, and the mental health impact in the form of PTED. Having said that, regressions show these variables only partially predict PTED and morally injurious experiences (19 – 37%) and so support for theoretical models highlighting their importance is limited.[8, 15, 16]

These findings allow for several recommendations. First, some individuals may recover from moral injuries and feelings of embitterment on their own, but others will need psychological support and clinicians should be aware that these individuals are relatively treatment resistant and have different treatment needs.[8,10,11] Self-care strategies should focus

For peer review only - http://bmjopen.bmj.com/site/about/guidelines.xhtml

on increasing self-esteem and reducing tendencies to think about the consequences of one's actions, in line with this study's findings. More importantly though, there should be an emphasis not on the individual to 'recover' but on healthcare leaders to tackle systemic issues that are causing moral injuries and embitterment in the first place. In this study, staff across all levels of health and social care have reported feeling betrayed, morally violated, and that they had been treated unjustly and unfairly during the pandemic. A proportion of these responses have been directly linked to the working environment (e.g., lack of PPE and resources). Healthcare authorities should strive to maintain fair 'processes' even if reducing all workplace stressors is unachievable. At a time when the UK and other countries need a healthy workforce to respond to the pandemic and its aftermath, staff retention is a priority.

To strengthen prevalence estimates reported here, large scale cohort studies using randomly selected, representative samples are needed. Follow up data collection with these samples can establish whether prevalence estimates increase across time as the pandemic continues. Future studies should identify the cognitive, affective, and behavioural manifestations of PTED and moral injury in healthcare professionals to inform treatment and treatment efficacy then needs to be established. Although beyond the scope of this paper, future studies should investigate the overlap between PTED and moral injury symptoms using newly developed symptom scales, and their overlap with other similar constructs like burnout.[9]

In conclusion, we are among the first to provide prevalence estimates of exposure to MIEs and PTED in health and social care workers as a direct result of working during the COVID-19 pandemic. We provide evidence that those who are clinical support staff, exposed to occupational stressors, and have a lack of belief that they experienced fair processes are amongst the most at risk. These findings indicate that measures to reduce workplace stressors and

For peer review only - http://bmjopen.bmj.com/site/about/guidelines.xhtml

ensuring staff feel they receive fair procedures at work should be a priority of healthcare organisations.

Figure 1.
Heatmap showing Pearson's correlation coefficients of study variables.

For peer review only

For peer review only - http://bmjopen.bmj.com/site/about/guidelines.xhtml

Contributor and guarantor information:

CB designed the project and data collection tools, collected the data, cleaned, analysed and interpreted the data and drafted and revised the paper. She is guarantor. MM analysed and interpreted the data and revised the paper. JC designed the project and data collection tools, interpreted the data, and revised the paper. All authors gave final approval for the publication of this manuscript. The corresponding author attests that all listed authors meet authorship criteria and that no others meeting the criteria have been omitted.

Copyright:

The Corresponding Author has the right to grant on behalf of all authors and does grant on behalf of all authors, an exclusive licence (or non-exclusive for government employees) on a worldwide basis to the BMJ Publishing Group Ltd to permit this article (if accepted) to be published in BMJ editions and any other BMJPGL products and sublicences such use and exploit all subsidiary rights, as set out in our licence.

Competing interest statement:

All authors have completed the Unified Competing Interest form (available on request from the corresponding author) and declare: the lead author had funding support from the Economic and Social Research Council and University of Liverpool COVID-19 strategic funding for the submitted work; no financial relationships with any organisations that might have an interest in the submitted work in the previous three years; no other relationships or activities that could appear to have influenced the submitted work.

Funding:

For peer review only - http://bmjopen.bmj.com/site/about/guidelines.xhtml

CB was supported by the Economic and Social Research Council UK [ES/P000665/1] and this work received COVID-19 strategic funding from the University of Liverpool.

Role of sponsor:

The study sponsors did not play a role in the study design, management, data collection, analyses, in the interpretation of the data or in the writing of the manuscript or the decision to submit for publication.

Transparency declaration:

The lead author affirms that this manuscript is an honest, accurate, and transparent account of the study being reported; that no important aspects of the study have been omitted; and that any discrepancies from the study as planned have been explained.

Ethical approval:

Ethical approval for the study was granted by the University of Liverpool (reference: 7862) and all participants provided informed consent.

Data availability statement:

Data from this study will be made available upon reasonable request by contacting the corresponding author at hlcbrenn@liverpool.ac.uk. Data will be made available after an embargoed period of 12 months to permit planned analyses of the longitudinal dataset and will be available for 10 years in line with ethical considerations.

Dissemination declaration:

Results from this study will be disseminated to participants that request this feedback and will be disseminated to relevant audiences through University of Liverpool communications.

For peer review only

References:

1. John Hopkins University (JHU). Mortality Analyses–Coronavirus Resource Centre. JHU, 2020 (https://coronavirus.jhu.edu/data/mortality).

2. NHS England. NHS staff survey. https://www.nhsstaffsurveyresults.com (Accessed 5 Feb 2020).

3. De Kock JH, Latham HA, Leslie SJ, Grindle M, Munoz S-A, Ellis L, et al. A rapid review of the impact of COVID-19 on the mental health of healthcare workers: implications for supporting psychological well-being. *BMC Public Health* 2021;21:104. DOI: https://doi.org/10.1186/s12889-020-10070-3

4. Major A, Hlubocky FJ. Mental health of health care workers during the COVID-19 pandemic and evidence-based frameworks for mitigation: A rapid review. *medRxiv* 2021.01.03.21249166 [Preprint]. January 04, 2021 [cited 2021 Feb 5] DOI: https://doi.org/10.1101/2021.01.03.21249166.

5. Greene T, Harju-Seppänen J, Adeniji M, Steel C, Grey N, Brewin CR et al. Predictors and rates of PTSD, depression and anxiety in UK frontline health and social care workers during COVID-19. European Journal of Psychotraumatology 2021;12. DOI: 10.1080/20008198.2021.1882781

6. Greenberg N, Docherty M, Gnanapragasam S, Wessely S. Managing mental health challenges faced by healthcare workers during covid-19 pandemic. *BMJ* 2020;368:m1211. doi:10.1136/bmj.m1211

For peer review only - http://bmjopen.bmj.com/site/about/guidelines.xhtml

7. British Medical Association COVID-19 survey December 2020. https://www.bma.org.uk/media/3637/bma-covid-survey-results-all-doctors-dec-2020.pdf (Accessed 5 April 2021).

8. Litz BT, Stein N, Delaney E, Lebowitz L, Nash WP, Silva C, et al. Moral injury and moral repair in war veterans: A preliminary model and intervention strategy. *Clinical Psychology Review* 2009;29:695-706. DOI: https://doi.org/10.1016/j.cpr.2009.07.003

9. Dean W, Talbot S, Dean A. Reframing Clinician Distress: Moral Injury Not Burnout. Fed Pract. 2019;36(9):400-2.

10. Linden M, Baumann K, Rotter M, Schippan B. Diagnostic criteria and the standardized diagnostic interview for posttraumatic embitterment disorder (PTED). International Journal of Psychiatry in Clinical Practice. 2008;12(2):93-6. DOI: https://doi.org/10.1080/13651500701580478

11. Linden M, Baumann K, Lieberei B, Rotter M. The post-traumatic embitterment disorder Self-Rating Scale (PTED Scale). Clinical Psychology & Psychotherapy 2009;16:139-47.

12. Hines SE, Chin KH, Levine AR, Wickwire EM. Initiation of a survey of healthcare worker distress and moral injury at the onset of the COVID-19 surge. *Am J Ind Med* 2020;63:830-3. DOI: https://doi.org/10.1002/cpp.610

13. Zhizhong W, Koenig HG, Yan T, Jing W, Mu S, Hongyu L, et al. Psychometric properties of the moral injury symptom scale among Chinese health professionals during the COVID-19 pandemic. *BMC Psychiatry* 2020;20:556. DOI: https://doi.org/10.1186/s12888-020-02954-w

14. Lamb D, Gnanapragasam S, Greenberg N, Bhundia R, Carr E, Hotopf M et al. The psychosocial impact of COVID-19 pandemic on 4,378 UK healthcare workers and ancillary

For peer review only - http://bmjopen.bmj.com/site/about/guidelines.xhtml

staff: initial baseline data from a cohort study collected during the first wave of the pandemic. *medRxiv* 2021.01.03.21249166 [Preprint]. January 22, 2021 [cited 2021 March 23]

15. Dodek A, Barnow S. Personality as risk and resilience in embitterment reactions. In: Linden M, Maercker A, editors. Embitterment: Societal, psychological, and clinical perspectives. Vienna: Springer Vienna; 2011. p. 83-100.

16. McKay MT, Perry JL, Percy A, Cole JC. Evidence for the reliability and validity, and some support for the practical utility of the two-factor Consideration of Future Consequences Scale-14. *Personality and Individual Differences* 2016;98:133-136. https://doi.org/10.1016/j.paid.2016.03.097

17. National Health Service (NHS). Explore roles. Available from: https://www.healthcareers.nhs.uk/explore-roles. (Accessed 7 March 2022)

18. Nash WP, Marino Carper TL, Mills MA, Au T, Goldsmith A, Litz BT. Psychometric Evaluation of the Moral Injury Events Scale. Military Medicine 2013;178:646-52. DOI: https://doi.org/10.7205/MILMED-D-13-00017

19. Bryan CJ, Bryan AO, Anestis MD, Anestis JC, Green BA, Etienne N, et al. Measuring moral injury: Psychometric properties of the moral injury events scale in two military samples. Assessment 2016;23:557-70. DOI: https://doi.org/10.1177/1073191115590855

20. Scheier MF, Carver CS, Bridges MW. Distinguishing optimism from neuroticism (and trait anxiety, self-mastery, and self-esteem): a reevaluation of the Life Orientation Test. J Pers Soc Psychol 1994;67:1063-78. DOI: https://doi.org/10.1037/0022-3514.67.6.1063

For peer review only - http://bmjopen.bmj.com/site/about/guidelines.xhtml

21. Glaesmer H, Rief W, Martin A, et al. Psychometric properties and population-based norms of the Life Orientation Test Revised (LOT-R). Br. J. Health. Psychol. 2012;17;432-445. DOI: https://doi.org/10.1111/j.2044-8287.2011.02046.x

22. Rosenberg, M. (1965). Society and the adolescent self-image. Princeton, NJ: Princeton University Press

23. Sinclair VG, Wallston KA. The development and psychometric evaluation of the Brief Resilient Coping Scale. Assessment 2004;11:94-101. DOI: https://doi.org/10.1177/1073191103258144

24. Lucas T, Zhdanova L, Alexander S. Procedural and Distributive Justice Beliefs for Self and Others Assessment of a Four-Factor Individual Differences Model. Journal of Individual Differences 2011;32:14-25. DOI: https://doi.org/10.1027/1614-0001/a000032

25. Strathman A, Gleicher F, Boninger DS, Edwards CS. The consideration of future consequences: weighing immediate and distant outcomes of behavior. J Pers Soc Psychol 1994;66:742. DOI: https://doi.org/10.1037/0022-3514.66.4.742

26. Crowne DP, Marlowe D. A new scale of social desirability independent of psychopathology. Journal of consulting psychology 1960;24:349. DOI: https://doi.org/10.1037/h0047358

27. Little RJA. A test of missing completely at random for multivariate data with missing values. Journal of the American Statistical Association 1988;83:1198–1202

28. Ferguson CJ. An effect size primer: a guide for clinicians and researchers. Professional Psychology: Research and Practice 2016;5:532-538. DOI: https://doi.org/10.1037/14805-020

29. Cohen J. (1988). Statistical Power Analysis for the Behavioral Sciences (2nd ed.). Routledge. DOI: https://doi.org/10.4324/9780203771587

For peer review only - http://bmjopen.bmj.com/site/about/guidelines.xhtml

30. Perry J, Nicholls A, Clough P, Crust L. 2015. Assessing model fit: Caveats and recommendations for confirmatory factor analysis and exploratory structural equation modelling. Measurement in Physical Education and Exercise Science 2015;19:12–21. https://doi.org/10.1080/1091367X.2014.952370

31. Sensky T, Salimu R, Ballard J, Pereira D. Associations of chronic embitterment among NHS staff. Occupational Medicine 2015;65;431-436. DOI: https://doi.org/10.1093/occmed/kqv089

For peer review only

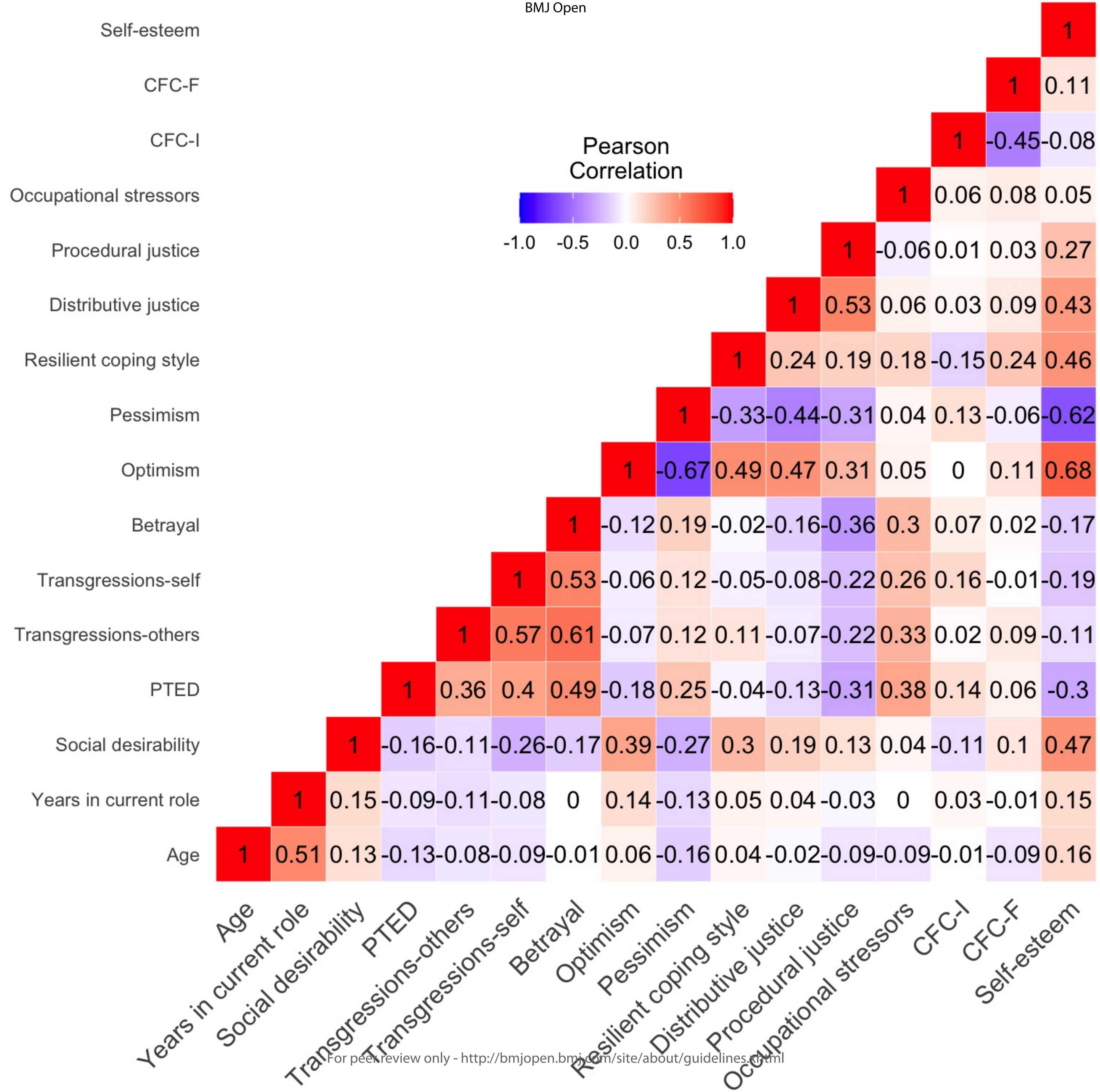
Pearson Correlation
-1.0 -0.5 0.0 0.5 1.0
Self-esteem
CFC-F
CFC-I
Occupational stressors
Procedural justice
Distributive justice
Resilient coping style
Pessimism
Optimism
Betrayal
Transgressions-self
Transgressions-others
PTED
Social desirability
Years in current role
Age

Supplementary tables

Supplementary table 1.
Socio-demographic characteristics of participants, and their association with PTED and the MIES subscales (95% CI with 2,000 bootstrapped samples)

	N	%	PTED	*p*	Effect size	Transgressions-others	*p*	Effect size	Transgressions-self	*p*	Effect size	Betrayal	*p*	Effect size
**Gender**[a]														
Male	98	24.5%	1.17 (1.0)	0.493	0.08	3.07 (1.53)	0.484	0.08	2.21 (1.17)	0.668	0.05	2.86 (1.43)	0.450	0.09
Female	302	75.5%	1.10 (0.87)			2.95 (1.47)			2.15 (1.24)			2.74 (1.36)		
**Professional role**[b]														
Allied health	58	14.5%	0.96 (0.92)	0.215	0.01	2.71 (1.44)	0.374	0.00	1.90 (1.05)	0.021[c]	0.04	2.55 (1.41)	0.589	0.00
Primary care practitioner	63	15.8%	1.04 (0.91)			3.07 (1.45)			2.32 (1.27)			2.87 (1.42)		
Managerial	41	10.3%	0.96 (0.87)			2.66 (1.58)			1.84 (1.25)			2.60 (1.63)		
Health info/science	37	9.3%	1.38 (1)			3.18 (1.53)			2.40 (1.27)			2.89 (1.43)		
Psychological	27	6.8%	1.11 (0.75)			2.83 (1.32)			1.79 (.98)			2.56 (1.08)		
Corporate	65	16.3%	1.07 (0.78)			3.08 (1.48)			2.13 (1.09)			2.81 (1.38)		
Clinical support	105	26.3%	1.23 (0.95)			3.14 (1.50)			2.43 (1.29)			2.92 (1.30)		
**Mental health**[a]														
Current mental health diagnoses	97	24.3%	1.52 (0.96)	<0.001[d]	0.64	3.20 (1.57)	0.100	0.20	2.31 (1.30)	0.177	0.15	3.10 (1.40)	0.013[e]	0.31
No current mental health diagnoses	303	75.8%	0.98 (0.84)			2.91 (1.45)			2.12 (1.20)			2.68 (1.36)		
**Ethnicity**[a]														
White	252	63%	1.09 (0.90)	0.628	0.07	2.93 (1.54)	0.390	0.13	2.08 (1.20)	0.124	0.23	2.77 (1.38)	0.951	0.01

For peer review only

Ethnic minorities	53	13.3%	1.15 (0.93)	3.13 (1.38)	2.36 (1.16)	2.76 (1.40)

Mean (SD) are reported. $_a$ Independent samples t-test reporting Cohen's *d* effect size, $_b$ One-way ANOVA reporting ω^2 effect size, $_c$ Welch's $F(6, 138.418) = 2.579$, $p=0.021$, $_d$ Mean difference -0.54 BCA 95% CI [-0.74, -0.34] $t(398) = -5.33$, $p<0.001$, $_e$ Mean difference -0.40 BCA 95% CI [-0.72, -0.07] $t(293) = -2.50$, $p=0.013$[1]

[1] Non-parametric Mann Whitney-U and Kruskal Wallis-H tests were also conducted. There were no differences in findings.

For peer review only

Supplementary table 2.

Severity and frequency of endorsement of items on the MIES and PTED scale in UK health and social care workers

MIES Item-level endorsement	Raw frequency (N)	% (95% CI)
I saw things that were morally wrong	179	44.8% (39.8 to 49.8%)
I am troubled by having witnessed others immoral acts	167	41.8% (36.9 to 46.8%)
I acted in ways that violated by own moral code or values	76	19% (15.3 to 23.2%)
I am troubled by having acted in ways that violated by own moral code or values	94	23.5% (19.4 to 28%)
I violated by own morals by failing to do something that I felt I should have done	83	20.8% (16.9 to 25.1%)
I am troubled because I violated by own morals by failing to do something that I felt I should have done	78	19.5% (15.7 to 23.7%)
I feel betrayed by superiors who I once trusted.	161	40.3% (35.4 to 45.2%)
I feel betrayed by fellow colleagues who I once trusted.	124	31% (26.5 to 35.8%)
I feel betrayed by others outside my work organisation who I once trusted.	158	39.5% (34.7 to 44.5%)
**PTED Item-level endorsement**	**Raw frequency (N)**	**% (95% CI)**
Any item endorsed by at “partially true” or greater level	293	73.3% (68.6 to 77.5%)
that hurt my feelings and caused considerable embitterment	142	35.5% (30.8 to 40.4%)
that led to a noticeable and persistent negative change in my mental well-being	167	41.8% (36.9 to 46.8%)
that I see as very unjust and unfair	170	42.5% (41.8 to 36.9%)
about which I have to think over and over again	167	41.8% (35.8 to 31%)
that causes me to be extremely upset when I am reminded of it	143	35.8% (31 to 40.7%)
that triggers me to harbour thoughts of revenge	44	11% (8.1 to 14.5%)
for which I blame and am angry with myself	87	21.8% (17.8 to 26.1%)
that led to the feeling that there is no sense to strive to make an effort	119	29.8% (25.3 to 34.5)
that makes me to frequently feel sullen or unhappy	151	37.8% (33 to 42.7%)
that impaired my overall physical well being	138	34.5% (29.8 to 39.4%)
that causes me to avoid certain places or persons so as to not be reminded of them	120	30% (25.5 to 34.8%)
that makes me feel helpless and disempowered	141	40.5% (35.7 to 45.5%)

For peer review only

that triggers feelings of satisfaction when I think that the responsible party having to experience a similar situation	76	19% (15.3 to 23.2%)
that led to a considerable decrease in my strength and drive	164	41% (36.1 to 46%)
that made that I am more easily irritated than before	176	44% (39.1 to 49%)
that makes that I must distract myself in order to experience a normal mood	160	40% (35.2 to 45%)
that made me unable to pursue occupational and/or family activities as before	176	44% (39.1 to 49%)
that caused me to draw back from friends and social activities	179	44.8% (39.8 to 49.8%)
which frequently evokes painful memories	118	29.5% (25.1 to 34.2%)

For item-level endorsement, each MIES item was coded as endorsed if the participant responded either “slightly agree” “moderately agree” or “strongly agree” and each PTED item was coded as endorsed if the participant responded either “partially true” “very much true” or “extremely true”.

For peer review only

Supplementary table 3.

Coefficients from the final step in hierarchical multiple regression analyses predicting PTED and transgressions-others, transgressions-self, and betrayal scores with 95% bias corrected and accelerated confidence intervals (2000 samples) (N=394).

	PTED			Transgressions-others			Transgressions-self			Betrayal		
Predictors	β	*B* 95% CI	*p*	β	*B* 95% CI	*p*	β	*B* 95% CI	*p*	β	*B* 95% CI	*p*
Age	-0.01	(-0.01 to 0.01)	0.789	0.04	(-0.01 to 0.02)	0.555	0.03	(-0.01 to 0.02)	0.587	0.04	(-0.01 to 0.02)	0.392
Current mental health diagnoses	0.12	(0.07 to 0.46)	**0.006**	-0.01	(-0.43 to 0.28)	0.835	-0.04	(-0.39 to 0.18)	0.512	0.04	(-0.25 to 0.45)	0.404
Social desirability	-0.02	(-0.03 to 0.02)	0.729	-0.08	(-0.10 to 0.01)	0.128	-0.21	(-0.14 to -0.05)	**0.002**	-0.13	(-0.11 to -0.01)	**0.012**
Years in role	-0.02	(-0.02 to 0.01)	0.747	-0.10	(-0.05 to 0.01)	0.110	-0.08	(-0.04 to 0.01)	0.185	0.01	(-0.02 to 0.02)	0.922
Allied	-0.12	(-0.60 to -0.09)	**0.036**	-0.12	(-0.93 to -0.09)	**0.020**	-0.16	(-0.87 to -0.15)	**0.002**	-0.10	(-0.71 to 0.00)	**0.036**
Primary care practitioner	-0.11	(-0.54 to -0.04)	**0.030**	-0.07	(-0.72 to 0.14)	0.201	-0.09	(-0.70 to 0.12)	0.185	-0.05	(-0.69 to 0.20)	0.434
Managerial	-0.04	(-0.37 to 0.15)	0.398	-0.11	(-1.12 to 0.04)	0.077	-0.13	(-0.91 to -0.03)	**0.028**	-0.04	(-0.64 to 0.39)	0.476
Health info/science	0.06	(-0.10 to 0.46)	0.157	0.03	(-0.40 to 0.69)	0.573	-0.01	(-0.47 to 0.43)	0.939	0.01	(-0.43 to 0.57)	0.819
Psychological	0.04	(-0.12 to 0.50)	0.307	-0.02	(-0.67 to 0.37)	0.581	-0.09	(-0.92 to 0.04)	0.061	-0.01	(-0.47 to 0.46)	0.934
Corporate	-0.02	(-0.29 to 0.21)	0.681	0.00	(-0.49 to 0.42)	0.978	-0.08	(-0.58 to 0.09)	0.142	-0.02	(-0.43 to 0.38)	0.747
Occupational stressors	0.38	(0.08 to 0.12)	**0.006**	0.32	(0.10 to 0.18)	**0.002**	0.26	(0.06 to 0.13)	**0.002**	0.29	(0.08 to 0.15)	**0.006**
Optimism	0.03	(-0.04 to 0.06)	0.681	-0.01	(-0.09 to 0.08)	0.876	0.15	(-0.01 to 0.13)	0.073	.007	(-0.04 to 0.14)	0.386
Pessimism	-0.01	(-0.05 to 0.05)	0.843	0.01	(-0.06 to 0.08)	0.888	-0.04	(-0.08 to 0.05)	0.644	.005	(-0.03 to 0.10)	0.470
Resilient coping style	0.06	(-0.01 to 0.06)	0.265	0.15	(0.03 to 0.16)	**0.014**	0.02	(-0.05 to 0.06)	0.746	.005	(-0.03 to 0.10)	0.410
Distributive justice	0.13	(0.01 to 0.04)	**0.024**	0.08	(-0.01 to 0.06)	0.163	0.08	(-0.01 to 0.05)	0.220	.007	(-0.01 to 0.06)	0.217
Procedural justice	-0.29	(-0.07 to -0.04)	**0.006**	-0.23	(-0.11 to -0.03)	**0.002**	-0.23	(-0.09 to -0.03)	**0.002**	-0.35	(-0.13 to -0.07)	**0.006**

For peer review only

CFC-I	0.14	(0.01 to 0.03)	**0.012**	0.03	(-0.02 to 0.03)	0.565	0.12	(0.01 to 0.04)	**0.037**	0.05	(-0.01 to 0.03)	0.355
CFC-F	0.11	(0.01 to 0.03)	**0.036**	0.08	(-0.01 to 0.04)	0.130	0.07	(-0.01 to 0.03)	0.262	0.05	(-0.01 to -0.03)	0.331
Self-esteem	-0.28	(-.07 to -.02)	**0.006**	-0.09	(-0.07 to 0.01)	0.207	-0.19	(-0.07 to -0.01)	**0.016**	-0.09	(-0.06 to 0.02)	0.241

Reference groups: Current mental health diagnoses (none), Professional role (clinical support role)
PTED = post-traumatic embitterment disorder; transgressions-others = Moral injury transgression by others; transgressions-self = Moral injury transgression by self; betrayal = Moral injury betrayal; CFC-I = Consideration of future consequences-Immediate; CFC-F = Consideration of future consequences-Future.
Significant p values are in bold.

For peer review only

Supplementary table 4.
Coefficients from the final step in hierarchical regression analyses using ***only significant predictors*** *of PTED, transgressions-others, transgressions-self, and betrayal scores with 95% bias corrected and accelerated confidence intervals (2000 samples) (N=394 to 396).*

PTED [a]				Transgressions-others [a]				Transgressions-self [b]				Betrayal [b]			
Predictors	β	*B* 95% CI	*p*	Predictors	β	*B* 95% CI	*p*	Predictors	β	*B* 95% CI	*p*	Predictors	β	*B* 95% CI	*p*
Current mental health diagnoses	0.12	(0.06 to 0.50)	**0.006**	Allied	-0.13	(-0.94 to -0.13)	**0.018**	Allied	-0.15	(-0.82 to -0.21)	**0.005**	Allied	-0.10	(-0.78 to 0.01)	**0.043**
Allied	-0.12	(-0.55 to -0.03)	**0.019**	Primary care practitioner	-0.07	(-0.77 to 0.19)	0.206	Primary care practitioner	-0.08	(-0.63 to 0.12)	0.190	Primary care practitioner	-0.05	(-0.63 to 0.23)	0.317
Primary care practitioner	-0.12	(-0.51 to -0.03)	**0.024**	Managerial	-0.10	(-0.99 to 0.10)	0.086	Managerial	-0.10	(-0.84 to 0.03)	0.064	Managerial	-0.05	(-0.66 to 0.29)	0.385
Managerial	-0.03	(-0.35 to 0.13)	0.466	Health info/science	0.04	(-0.30 to 0.77)	0.369	Health info/science	0.01	(-0.44 to 0.46)	0.991	Health info/science	0.01	(-0.38 to 0.44)	0.882
Health info/science	0.06	(-0.07 to 0.50)	0.188	Psychological	0.01	(-0.49 to 0.48)	0.976	Psychological	-0.08	(-0.85 to 0.07)	0.080	Psychological	-0.01	(-0.53 to 0.33)	0.724
Psychological	0.04	(-0.16 to 0.45)	0.344	Corporate	0.02	(-0.31 to 0.47)	0.690	Corporate	-0.06	(-0.52 to 0.13)	0.214	Corporate	-0.02	(-0.47 to 0.28)	0.675

Corporate	-0.12	(-0.28 to 0.19)	0.727	Occupational stressors	0.34	(0.11 to 0.19)	**0.001**	Social desirability	-0.20	(-0.13 to 0.04)	**0.001**	Social desirability	-0.14	(-0.11 to -0.02)	**0.007**
Occupational stressors	0.39	(0.08 to 0.12)	**0.002**	Resilient coping style	0.11	(0.01 to 0.12)	**0.021**	Occupational stressors	0.27	(0.06 to 0.14)	**0.001**	Occupational stressors	0.31	(0.09 to 0.16)	**0.001**
Distributive justice	0.14	(0.01 to 0.04)	**0.006**	Procedural justice	-0.21	(-0.10 to -0.04)	**0.001**	Procedural justice	-0.16	(-0.07 to -0.01)	**0.001**	Procedural justice	-0.32	(-0.12 to -0.07)	**0.001**
Procedural justice	-0.28	(-0.07 to -0.03)	**0.002**					CFC-I	0.10	(-0.01 to 0.03)	**0.046**				
CFC-I	0.13	(0.01 to 0.03)	**0.008**					Self-esteem	-0.04	(-0.03 to 0.01)	0.427				
CFC-F	0.12	(0.01 to 0.03)	**0.006**												
Self-esteem	-0.25	(-0.05 to -0.02)	**0.002**												

Reference groups: Current mental health diagnoses (none), Professional role (clinical support role)

PTED = post-traumatic embitterment disorder; transgressions-others = Moral injury transgression by others; transgressions-self = Moral injury transgression by self; betrayal = Moral injury betrayal; CFC-I = Consideration of future consequences-Immediate; CFC-F = Consideration of future consequences-Future.

Significant p values are in bold.

[a] *N*=396

[b] *N*=394

Supplementary information: Confirmatory factor analysis for all study variables

Confirmatory factor analyses (CFA) for all instruments used in the present study were carried out with AMOS 26, and all estimations are based on maximum likelihood procedures. To assess model fit, we examined incremental fit indices of the comparative fit index (CFI) and Tucker-Lewis index (TLI) and the absolute fit indices of root-mean square error of approximation (RMSEA) and standardised root mean square residual (SRMR). In line with recommendations by Perry, Nicholls, Clough, and Crust[30] no arbitrary value was used as a cut-off. Instead, CFI and TLI close to 0.90 and SRMR and RMSEA close to zero (i.e., <0.06) were interpreted as representing adequate model fit.

Moral Injury Events Scale (MIES)

There is ongoing debate regarding model fit for the MIES, with a ‘transgressions’ (items 1-6) and ‘betrayal’ (i.e., items 7-9) two factor model proposed by Nash and colleagues[18], a ‘transgression-other’ (i.e., items 1,2,7,8, and 9) and ‘transgression-self’ two factor model (i.e., items 3-6) proposed by Richardson et al., and a three factor model proposed by Bryan et al.,[19](i.e., transgressions-others [items 1-2], transgressions-self [items 3-6] and betrayal [items 7-9]. We tested all three. Fit indices for Nash’s initial two factor structure was suboptimal: $\chi^2(26) = 211.257$, CFI = .917, p<0.001, TLI = 0.886, SRMR = 0.087, RMSEA (90% CI) = 0.134 (0.117, 0.151). The two-factor structure proposed by Richardson showed an improvement in model fit: $\chi^2(26) = 104.894$, p<0.001, CFI = 0.965, TLI = 0.951, SRMR = 0.041, RMSEA (90% CI) = 0.09 (0.07, 0.11). However, the three-factor solution proposed by Bryan had superior model fit and was used in subsequent analyses: $\chi^2(24) = 36.217$, p=0.052, CFI = 0.995, TLI = 0.992, SRMR = 0.017, RMSEA (90% CI) = 0.036 (0.000, 0.058).

Post-traumatic embitterment scale (PTED)

The PTED scale is employed as a unidimensional scale and fit indices indicated that model fit was permissible despite some measurement error in the absolute fit indices, $\chi^2(152)$

= 617.373, p<0.001, CFI = 0.924, TLI = 0.915, SRMR = 0.044, RMSEA (90% CI) = 0.088 (0.080, 0.095).

Exposure to occupational stressors scale

For this study we developed a measure assessing exposure to occupational stressors. A unidimensional model in which all items loaded onto one latent factor presented a satisfactory model fit: $\chi^2(5) = 35.32$, p<0.001, CFI = 0.944, TLI = 0.934, SRMR = 0.059, RMSEA (90% CI) = 0.051 (0.036, 0.066). This is noteworthy given that this was a newly developed instrument.

Revised Life Orientation Test (LOT-R)

The LOT-R can be used as a unidimensional scale by reverse-scoring pessimism items to generate an overall optimism score. However, Glaesmer et al.,[21] argued for the use of a two-factor model. We tested both and found the unidimensional model fit to be unsatisfactory: $\chi^2(9) = 98.909$, p<0.001 CFI = 0.925, TLI = 0.875, SRMR = 0.055, RMSEA (90% CI) = 0.158 (0.131, 0.187). The two-factor model presented a significant improvement and was used in subsequent analyses: $\chi^2(8) = 23.268$, p=0.003 CFI = 0.987, TLI = 0.976, SRMR = 0.027, RMSEA (90% CI) = 0.069 (0.037, 0.103).

Rosenberg Self-esteem Scale

The Rosenberg self-esteem is primarily employed as a unidimensional scale with negatively worded items reverse scored. The unidimensional scale displayed some measurement error, primarily in the absolute fit indices: $\chi^2(35) = 203.48$, p<0.001, CFI = 0.920, TLI = 0.897, SRMR = 0.049, RMSEA (90% CI) = 0.110 (0.095, 0.125). However, all factor loadings were significant.

Brief Resilience Coping Scale

For peer review only

The unidimensional factor structure of the brief resilient coping scale demonstrated good model fit $\chi^2(2) = 3.243$ p=.198, CFI = .993, TLI = 0.980, SRMR = 0.021, RMSEA (90% CI) = 0.039 (0.000, 0.115).

Personal belief in a just world scale (personal BJW)

According to Lucas, Zhdanova and Alexander[24] the personal BJW is comprised of two factors, distributive justice (distributive justice; items 1-4) and procedural justice (procedural justice; items 5-8). First, we tested a unidimensional model in which all items loaded onto a single latent factor and found this model was poor, $\chi^2(20) = 802.839$, CFI = 0.723, TLI = 0.612, SRMR = 0.164, RMSEA (90% CI) = 0.313 (0.295, 0.332). The two-factor model displayed some measurement error as shown by the absolute fit indices but had much better fit; $\chi^2(19) = 144.221$, CFI = 0.956, TLI = 0.935, SRMR = 0.027, RMSEA (90% CI) = 0.129 (0.109, 0.149). The two sub-scales were used in subsequent analyses.

Consideration of future consequences-14 scale (CFC-14)

Given the on-going question regarding the model fit for the CFC scale[16] we examined a one-factor and a two-factor model. Model fit for a one-factor solution was less than ideal: $\chi^2(77)= 594.219$, $p < 0.001$; CFI = 0.749; TLI = 0.704; RMSEA = 0.130 (95% CI = 0.120, 0.140); SRMR = 0.105. Conversely, fit indices for the two-factor solution were acceptable and used in subsequent analyses: χ^2 (76)=179.026, $p < 0.001$; CFI = 0.950; TLI = 0.940; RMSEA = 0.058 (95% CI = 0.047, 0.069); SRMR = 0.059.

Social Desirability scale

The unidimensional factor structure of the social desirability scale demonstrated some measurement error in the comparative fit indices: $\chi^2(65) = 130.815$, p<0.001, CFI = 0.861, TLI = 0.833, SRMR = 0.051, RMSEA (90% CI) = 0.051 (0.038, 0.163) and item 13 had a relatively poor factor loading (i.e., 0.28). Despite this, all factor loadings did significantly load onto their latent factor.

For peer review only

Checklist for Reporting Results of Internet E-Surveys (CHERRIES)

Checklist Item	***Explanation***
Describe survey design	We conducted a longitudinal voluntary survey, using a purposive convenience sample of UK health care workers who worked during the COVID-19 pandemic. This paper reports the cross-sectional analysis of baseline data.
IRB approval	Ethical approval was granted by the Ethics Committee at The University of Liverpool.
Informed consent	Study information was provided on Prolific, then again on the electronic information sheet. The information sheet detailed study information, the identity of the investigators, the time investment for the survey and that completing, that data would be held for a maximum of ten years, and that data was anonymous. Participants then provided electronic informed consent.
Data protection	No personally identifiable information was collected.
Development and testing	An anonymous electronic questionnaire was designed by the authors. Primarily validated scales from the literature were used. The web survey was tested prior to the study commencing by colleagues to estimate timing and ensure the survey was readable and usable.
Open survey versus closed survey	Data was collected using a closed survey, only individuals registered with Prolific, that met certain eligibility criteria (i.e., fluent in English, located in the UK, and work within health and social care assistance or medical/healthcare industries) were allowed to take part in the survey.
Contact mode	Initial contact with participants was made on the Internet, using the Prolific website.
Advertising the survey	The survey was advertised on Prolific, to those meeting pre-defined eligibility criteria (i.e., fluent in English, located in the UK, and work within health and social care assistance or medical/healthcare industries). The advertisement informed participants that the aim of the study was to explore factors influencing the onset of mental health difficulties following the COVID-19 pandemic, and that they would be invited to take part in a second round of data collection in 12 months. They were informed of the eligibility criteria and of the financial incentive to take part, they would receive £2.50 for taking part in survey one, and a further £2.50 for taking part in survey two in 12 months.
Web/E-mail	This was an electronic web survey using the Qualtrics platform. Captured responses were automatically recorded into a database.

For peer review only

Context	The survey was posted on Prolific. Prolific is an online crowdsourcing platform which holds a database of potential participants to take part in studies. Potential participants have answered demographic questions about themselves, and these can be used to select for certain individuals. Individuals meeting these criteria are then sent the study advertisement by Prolific.
Mandatory/voluntary	The survey was completely voluntary. Participants did not need to take part and could withdraw from the survey at any time by exiting the browser.
Incentives	Participants received £2.50 for completing the survey in line with platform guidelines.
Time/Date	Data was collected from 24th September – 6th October 2020.
Randomization of items or questionnaires	To prevent biases, the order of the questionnaires was randomized and the items within the questionnaires were randomized.
Adaptive questioning	Adaptive questioning was not used.
Number of Items	The number of items per page varied from 1 to 19.
Number of screens (pages)	Excluding the information, consent and debrief sheets, the survey consisted of 11 pages.
Completeness check	Completeness was checked after survey completion.
Review step	There was no review stage where participants could review their answers.
Unique site visitor	All participants could only complete the survey once, through Prolific. Prolific uses several tracking mechanisms including IP address detection to ensure that no participant takes part in a study more than once.
View rate (Ratio of unique survey visitors/unique site visitors)	Not applicable.
Participation rate (Ratio of unique visitors who agreed to participate/unique	All participants that viewed the survey page agreed to participate. This is a 100% recruitment rate.

For peer review only

first survey page visitors)	
Completion rate (Ratio of users who finished the survey/users who agreed to participate)	Out of 447 people that agreed to participate, 47 dropped out and did not fully complete the survey. This is a completion rate of 89.49%.
Cookies used	Not used. Prolific prevents multiple entries from the same individual using IP addresses, The Internet Service Provider (ISP), Device and browser information and by not allowing VPN or proxy usage.
IP check	Prolific, which is the website we used for recruitment uses IP addresses to identify potential duplicate entries from the same user. However, we do not have access to information on how exactly they use this information. In addition, Qualtrics which was used to host the survey automatically recorded the participants Prolific ID code, so we know that no individual took part in the survey twice.
Log file analysis	Not applicable.
Registration	Users needed to have a registered account with Prolific and meet the eligibility criteria we set to receive a notification that they were eligible to take part in the study. Each user has a unique ID code, which was automatically recorded by Qualtrics if they entered the survey. If they completed the survey until the end, they were automatically redirected back to Prolific, where the system automatically logs a completion code so that we could provide the participant with their payment. The Prolific system does not allow multiple entries using the methods discussed above, and we were also able to check using their ID codes that no individual took part more than once.
Handling of incomplete questionnaires	Only complete questionnaires were analysed since individuals that did not fully complete were not provided with the financial reimbursement. It would be unethical to use unpaid responses in analyses.

For peer review only

Questionnaires submitted with an atypical timestamp	Researchers provided Prolific with an estimation of the time it took to complete the survey. We estimated 20 minutes. Prolific then automatically estimates a maximum time allocation based on this information. It allowed participants a maximum time of 67 minutes to take part in our survey, after that they will be timed out because they have become inactive. This allows others to take part in the survey since there is a maximum place allowance.
Statistical correction	No statistical correction was applied.

For peer review only

BMJ Open

Morally injurious events and post-traumatic embitterment disorder in UK health and social care professionals during COVID-19: a cross sectional web survey

Journal:	*BMJ Open*
Manuscript ID	bmjopen-2021-054062.R2
Article Type:	Original research
Date Submitted by the Author:	14-Apr-2022
Complete List of Authors:	Brennan, Chloe; University of Liverpool, Department of Psychology<br>McKay, Michael; Royal College of Surgeons in Ireland<br>Cole, Jon; University of Liverpool, Department of Psychology
**Primary Subject Heading**:	Mental health
Secondary Subject Heading:	Health policy, Health services research
Keywords:	COVID-19, MENTAL HEALTH, PSYCHIATRY, Health policy < HEALTH SERVICES ADMINISTRATION & MANAGEMENT

SCHOLARONE™
Manuscripts

BMJ

I, the Submitting Author has the right to grant and does grant on behalf of all authors of the Work (as defined in the below author licence), an exclusive licence and/or a non-exclusive licence for contributions from authors who are: i) UK Crown employees; ii) where BMJ has agreed a CC-BY licence shall apply, and/or iii) in accordance with the terms applicable for US Federal Government officers or employees acting as part of their official duties; on a worldwide, perpetual, irrevocable, royalty-free basis to BMJ Publishing Group Ltd ("BMJ") its licensees and where the relevant Journal is co-owned by BMJ to the co-owners of the Journal, to publish the Work in this journal and any other BMJ products and to exploit all rights, as set out in our licence.

The Submitting Author accepts and understands that any supply made under these terms is made by BMJ to the Submitting Author unless you are acting as an employee on behalf of your employer or a postgraduate student of an affiliated institution which is paying any applicable article publishing charge ("APC") for Open Access articles. Where the Submitting Author wishes to make the Work available on an Open Access basis (and intends to pay the relevant APC), the terms of reuse of such Open Access shall be governed by a Creative Commons licence – details of these licences and which Creative Commons licence will apply to this Work are set out in our licence referred to above.

Other than as permitted in any relevant BMJ Author's Self Archiving Policies, I confirm this Work has not been accepted for publication elsewhere, is not being considered for publication elsewhere and does not duplicate material already published. I confirm all authors consent to publication of this Work and authorise the granting of this licence.

For peer review only

Morally injurious events and post-traumatic embitterment disorder in UK health and social care professionals during COVID-19: a cross sectional web survey

Chloe J. Brennan[1], Michael T. McKay[2], Jon C. Cole[1]

[1] Department of Psychology, University of Liverpool, United Kingdom

[2] Department of Psychology, Royal College of Surgeons in Ireland, Dublin, Ireland

For peer review only

Keywords: Mental health, Stress disorders, Post-Traumatic, Pandemics, Personality

Word count: 4268

[1] **Correspondence to**
Chloe Brennan
Eleanor Rathbone Building
Bedford Street South
Liverpool
L69 7ZT
Telephone: +44 (0)151 794 2000
hlcbrenn@liverpool.ac.uk

For peer review only - http://bmjopen.bmj.com/site/about/guidelines.xhtml

Abstract:

Objective

To estimate the prevalence and predictors of morally injurious events and post-traumatic embitterment disorder (PTED) in United Kingdom (UK) health and social care professionals during the COVID-19 pandemic.

Design

Cross-sectional study.

Setting

September-October 2020 in the United Kingdom. Online survey hosted on Qualtrics, and recruited through Prolific.

Participants

400 health and social care workers, aged 18 or above and living and working in the UK during the pandemic.

Main outcome measures

Morally injurious events were assessed using the Moral Injury Events Scale and PTED was assessed using the PTED self-rating scale. Potential predictors were measured using surveys of exposure to occupational stressors, optimism, self-esteem, resilient coping style, consideration of future consequences, and personal belief in a just world.

Results

19% of participants displayed clinical levels of PTED, and 73% experienced at least one COVID-related morally injurious event. Exposure to occupational stressors increased risk of experiencing PTED and morally injurious events, whereas personal belief in a procedurally just world, which is the belief that they experienced fair processes, was a protective mechanism.

For peer review only

Conclusions

Morally injurious events and PTED are being experienced by UK health and social care professionals, particularly in those exposed to work-related stressors.

Strengths and limitations:

- This represents the first prevalence estimates of both morally injurious events and PTED in UK health and social care workers during COVID-19.
- Findings are generalisable to health and social care workers across diverse job roles rather than only 'frontline' healthcare staff.
- To inform healthcare policy, validated scales identified demographic, occupational, and personality traits associated with an increased risk of morally injurious events and PTED.
- The cross-sectional design prevents analysis of causal relationships, and comparisons to pre-pandemic levels of PTED and morally injurious events are not possible.
- Despite controlling for social desirability in analyses, survey data suffers from response and social desirability bias.

For peer review only

Introduction:

By January 2021 the rapid spread of the novel coronavirus disease 2019 (COVID-19) had claimed the lives of 100,000 people in the UK.[1] In the year prior to the pandemic, UK healthcare workers (HCWs) reported work-related stress at its highest level since 2014.[2] The early psychological impact of COVID-19 on HCWs is not equivocally experienced as globally, rates of depression (8.9-50.4%), anxiety (14.5-44.6%), post-traumatic stress disorder (PTSD; 8.3-88.4%), stress (2.2-93.8%) and burnout (21.8-46.3%) in HCWs vary widely across studies.[3,4] Research has focused predominantly on HCWs, but preliminary evidence into UK health and social care workers (HSCWs) during the pandemic indicates that nearly two thirds met thresholds for clinically significant disorders (PTSD = 22%, anxiety = 47%; depression = 47%).[5]

Health research in the wake of the COVID-19 pandemic has largely focussed on symptoms of anxiety, depression, PTSD, and stress. However, it is arguable that an equally pressing health-related concern is the way in which HSCWs feel they have been treated throughout the pandemic, and the associated psychological impact. Individuals have risked their lives during shortages in staff, limited availability of personal protective equipment (PPE) and other resources, which can lead to feelings of injustice and increased pressure to make morally challenging decisions.[6] Doctors surveyed in December 2020 reported feeling unable to provide the right care at the right time, with around 40% experiencing feelings of anger/irritability.[7] These experiences have the potential to violate healthcare staff's ethical beliefs, placing them at an increased risk of experiencing moral injury and post-traumatic embitterment disorder (PTED).

Witnessing or perpetrating events that violate one's moral values are called '*morally injurious events*' (MIEs), and moral injury encapsulates symptoms such as guilt, shame, and poor

For peer review only - http://bmjopen.bmj.com/site/about/guidelines.xhtml

mental health outcomes associated with these events.[8] Traditionally, moral injury has been studied in military personnel after witnessing the horrors of war. However, there is growing recognition that moral injury can occur in civilian healthcare professionals, and some argue that morally injured healthcare staff are being inaccurately diagnosed with burnout.[9] PTED is a reactive disorder, triggered by an unjust event, and is accompanied by intrusive thoughts, mood impairment, social withdrawal, hostility, and feelings of helplessness.[10,11] Theoretically, PTED and moral injury are very similar. Their aetiologies are the same as both develop following an event(s) that has breached an individual's core beliefs. After the event(s), symptoms span the emotional (i.e., moral emotions of anger and shame), cognitive (i.e., negative beliefs about oneself, others, and the world) and social (i.e., relational) domains.

The biggest distinction between PTED and moral injury is their approach to psychiatric diagnoses. Most PTED researchers agree that embitterment should be a separate psychiatric disorder in diagnostic manuals if it causes significant impairment to daily functioning for over 6 months.[10] In contrast, some moral injury researchers are hesitant to pathologize adaptive moral reactions to ethical issues and so moral injury is often considered a risk factor for other severe mental health issues.[6] Similarly, a useful conceptualisation of the PTED-moral injury relationship may be that exposure to MIEs can lead to PTED. Analysing this relationship can advance our understanding of moral trauma and provide a useful diagnosis for treatment resistant patients exhibiting these symptoms.

The British Medical Association has suggested that moral injury may become one of the most significant injuries for healthcare staff responding to the COVID-19 pandemic, and preliminary evidence in the United States (US)[12] and China[13] appears to support this claim. Initial data from National Health Service (NHS) check, a cohort study of UK healthcare staff

For peer review only - http://bmjopen.bmj.com/site/about/guidelines.xhtml

during the pandemic, indicates that MIEs are linked to increased rates of mental health symptoms.[14] However, there is a lack of studies in the UK investigating the prevalence of both moral injury and PTED in healthcare staff, despite their similarities. Additionally, despite an increased risk, not all staff members will develop PTED and/or moral injury as a result of their experiences during the pandemic. The literature suggests that individuals high in optimism, self-esteem, resilient coping, and belief in a just world, are at a reduced risk of experiencing PTED and/or moral injury,[8,15] and individuals who consider the future consequences of their behaviour have better health outcomes.[16] The current study reports on available baseline findings from a longitudinal study of UK health and social care workers that began in September-October 2020. The study aims to establish the prevalence of PTED and morally injurious events and identify potential risk and protective factors. Further waves of this study will investigate whether factors at baseline predict future PTED and/or morally injurious events, establish whether prevalence rates increase across time as the pandemic continues and investigate whether morally injurious events at baseline predict subsequent PTED.

Methods:

Design:

This is a cross-sectional analysis of baseline data gathered after the first peak of COVID-19 in the UK (24th September – 6th October 2020). Ethical approval was granted by the Ethics Committee at The University of Liverpool.

Participants and procedure:

Participants were 400 HSCWs, aged 18-67, residing in the UK, and employed during the COVID-19 pandemic. Job roles were categorised in line with careers on the NHS website[17]

For peer review only

(see supplementary Table 1 for self-reported demographics). Participants were recruited via Prolific, an online crowdsourcing platform and received £2.50 for completing the survey (in line with platform guidelines). A further wave of data will take place at a 12-month follow up. Participants that volunteered were directed to an online survey hosted on Qualtrics where they provided informed consent, responded to psychological measures in a randomised order, were debriefed, and redirected back to Prolific. Prolific ensures no duplicate responders using IP address tracking. Due to participants potentially experiencing considerable levels of PTED and/or MIEs, appropriate support links and researchers' contact details were provided on the information and debrief sheets. The survey took approximately 15 minutes to complete. A power calculation conducted in G*Power found that 195 participants were required to find a small to moderate effect size of $f^2 = 0.12$ with power 0.80 and $\alpha=0.05$. To account for attrition rates of >50% in longitudinal studies, 400 participants were recruited. Completeness was checked after survey completion, and incomplete responses were not analysed. This included participants that Prolific automatically eliminated because they took too long to complete the survey. Prior to study completion, 47 out of 447 participants dropped out, which is a response rate of 89.5%. To minimise non-response bias, study information was clearly communicated, and the survey was short.

Patient and Public involvement:

No patients or the public were involved in the design or conduct of this study.

Materials:

Ninety-eight survey items were distributed across 11 pages, and items per page ranged from 1 to 19.

For peer review only - http://bmjopen.bmj.com/site/about/guidelines.xhtml

Participants self-reported their age, gender, ethnicity, professional role, years in current role, and any current mental health diagnoses.

Morally injurious events were assessed using the Moral Injury Events Scale (MIES),[18] a nine-item scale measuring exposure to military MIEs that violate moral beliefs. A higher score indicated more exposure to morally injurious events. The scale was adapted to reflect a health and social care sample in two ways. First, participants were asked to respond regarding their experiences as a HSCW professional since the outbreak of COVID-19. Second, three items assessing betrayal by “leaders”, “fellow service members” and “others outside of the U.S. military” in the original scale were changed to “superiors” “fellow colleagues” and “others outside of my work organisation”. Ratings were made on a 6-point Likert scale, ranging from 1 (*strongly disagree*) to 6 (*strongly agree*). A recent factor analysis found three reliable subscales of the MIES; (1) transgressions by others (transgression-others; $\alpha = 0.79$), (2) transgressions by self (transgressions-self; $\alpha = 0.94$), and (3) betrayal ($a = 0.89$).[19] The mean scores across items in each subscale were used in analyses. For prevalence estimates, we calculated the percentage of participants who “slightly agreed”, “agreed”, or “strongly agreed” with each item of the MIES.

PTED was assessed using the PTED Self-Rating Scale,[11] which is a 19-item scale assessing embitterment following negative life events. Participants were asked to respond regarding working during the COVID-19 pandemic on a 5-point scale ranging from 0 (*not true at all*) to 4 (*extremely true*). The mean score across the 19 items was used in analyses relating to PTED. In line with recommendations, [11] a mean total score of ≥ 2 was considered to be indicative of clinically relevant levels of PTED.

To measure exposure to occupational stressors, a five-item scale was developed for this study. Participants indicated on a 4-point scale (1 = *not at all*, 4 = *all the time*) how often they

For peer review only - http://bmjopen.bmj.com/site/about/guidelines.xhtml

experienced: (1) exposure to COVID-19 in their job; (2) a lack of PPE and/or clear training; (3) having to make difficult decisions regarding resource allocation; (4) an inability to provide adequate care or save lives; and (5) separation from, or fear for, loved ones due to working during the current pandemic. Items were summed, with higher scores reflecting higher exposure.

The Revised Life Orientation Test (LOT-R)[20] is a ten-item scale assessing generalised optimism. Responses (0 = *strongly disagree*, 4 = *strongly agree*) to four filler items were removed. As a result of confirmatory factor analysis (see Supplementary materials) we employed a two-factor model suggested by Glaesmer et al.,[21] where the sum of three items reflected higher optimism scores and the sum of three items reflected higher pessimism scores.

The Rosenberg (1965) Self-Esteem Scale[22] asked participants to indicate their agreement to ten items using a 4-point Likert scale where 1 = *strongly disagree* and 4 = *strongly agree*. Five negatively worded items were recoded so that higher total scores reflected higher self-esteem.

The Brief Resilience Coping Scale[23] is a short scale measuring tendency to cope with stress adaptively. Four items were assessed on a 5-point Likert scale (1 = *does not describe at all*, 4 = *describes me very well*). Total scores range from 4-20, with high scores indicating resilient coping ability.

The Belief in a Just World Scale (BJW)[24] contains eight items measuring perceptions of fairness towards oneself (personal BJW) and eight items measuring perceptions of fairness towards others (general BJW). Personal BJW better predicts psychological well-being so only this scale was used. These eight items were rated on a 7-point Likert scale from 1 (*strongly disagree*) to 7 (*strongly agree*). Four items measure distributive justice and four items measure procedural justice.

For peer review only - http://bmjopen.bmj.com/site/about/guidelines.xhtml

The Consideration of Future Consequences-14 Scale (CFC-14)[25] uses the sum of seven items to assess consideration of future consequences (CFC-F; α = 0.82) and the sum of seven items to assess consideration of immediate consequences (CFC-I; α = 0.82). Responses are given on a 7-point Likert ranging from 1 (*very unlike me*) to 7 (*very much like me*) and no items are reverse scored.

Social desirability was measured using the 13-item Marlowe-Crowne Social Desirability Scale.[26] Participants indicated either '*True*' (0) or '*False*' (1) and five items were reverse coded so higher scores reflected more socially desirable responses. Social desirability was controlled for in hierarchical regression analyses so no participants were excluded based on high scores.

Data analysis:

Based on confirmatory factor analyses (CFA), we employed a three-factor solution for the MIES, and a two-factor solution for the LOT-R, the personal BJW and the CFC-14. All other scales used a unidimensional structure (see Supplementary materials). Prevalence estimates for PTED and MIES were calculated followed by Pearson's bivariate correlations to examine the relatedness of these constructs, and their relationship to predictor variables. Independent samples t-tests and a one-way ANOVA examined the impact of sociodemographic variables on PTED and MIES scores. Four hierarchical linear regressions were then conducted for each dependent variable (PTED, transgressions-others, transgressions-self, and betrayal) to assess the unique effects of demographics and social desirability (step 1), occupational stressors (step 2), and personality characteristics (step 3). Pairwise deletion was used in univariate analyses, and listwise deletion in multivariate analyses. Bias-corrected and accelerated (BCa) bootstrapping methods were applied to all analyses to deal with violations to normality, outliers and homoscedacity of variance, and all

For peer review only - http://bmjopen.bmj.com/site/about/guidelines.xhtml

other assumptions were met. For all analyses p <0.05 was deemed significant and we used Ferguson's[27] recommendations for minimum practical ($d \geq 0.41$, $r \geq 0.20$, $\omega^2 \geq 0.04$,), medium ($d \geq 1.15$, $r \geq 0.50$, $\omega^2 \geq 0.25$,), and large effect sizes ($d \geq 2.70$, $r \geq 0.80$, $\omega^2 \geq 0.64$). Cohen's f^2[28] was used to interpret the effect size of overall regressions, where $f^2 \geq 0.02$, $f^2 \geq 0.15$, and $f^2 \geq 0.35$ represented small, medium, and large effect sizes. Recommendations by Perry et al.,[29] were used to interpret CFA where incremental fit indices close to 0.90 and absolute fit indices close to zero (i.e., <0.06) represented adequate model fit. Analyses were carried out using IBM SPSS v26, AMOS v26, or R v4.1.0.

<u>Results:</u>

Of the 400 responses recorded, there was <1% missing data for social desirability and professional role, and 23.8% missing data for ethnicity, which was Missing Completely at Random (MCAR), $\chi(15) = 7.737$, p= 0.934[30].

Prevalence of MIES, PTED, and exposure to occupational stressors

In this sample, 72.3% [95% CI 67.6% to 76.6%] experienced at least one morally injurious event. On the PTED scale, using the clinical cut off score of ≥2[11] 19.3% [95% CI 15.5% to 23.5%] of the sample were embittered (see Table 1). For item level prevalence estimates see supplementary Table 2. The average score on the MIES was 2.55 (SD = 1.13) with highest scores observed on the transgressions-others subscale, followed by betrayal, and transgressions-self (see Table 2). Prevalence of exposure to occupational stressors at "occasionally" or above on the Exposure scale revealed that 69.8% of participants reported being exposed to COVID-19 in their job, 57.5% were affected by a lack of PPE or COVID-19 training, 56.8% reported making difficult resource allocation decisions, 56.3% reporting feeling unable to

For peer review only - http://bmjopen.bmj.com/site/about/guidelines.xhtml

provide adequate care or save patients they usually would, and 64% reported experiencing separation from, or fear for, loved ones due to working during the pandemic.

Univariate association between MIES scores, PTED scores, and predictor variables

Table 2 displays mean scores, and internal consistency estimates, and Figure 1 displays a Pearson's correlation heatmap of continuous study variables. Supplementary Table 1 displays univariate tests of differences between categorical demographic variables and PTED and MIES scores.

PTED was positively associated with all sub-scales of the MIES, and the largest association was with betrayal (*p's*<0.001, see Figure 1). These associations approached moderate effect sizes (r = 0.36 to 0.49) indicating that PTED and MIEs are distinct, but related psychological constructs.

PTED was associated with older age (p = 0.011) and current self-reported mental health diagnoses (p<0.001), transgressions-others was associated with fewer years in current role (p = 0.033), betrayal was associated with current self-reported mental health diagnoses (p = 0.013), and transgressions-self was associated with professional role (p = 0.021) though no roles were significantly different from each other when applying Games-Howell correction for multiple comparisons. Risk factors for all dependent variables included exposure to occupational stressors (*p's*<0.001) and pessimism (p<0.001 with PTED and betrayal, p = 0.014 with transgressions-others and p = 0.015 with transgressions-self). CFC-I was a risk factor for PTED (p = 0.005) and transgressions-self (p = 0.001), and resilient coping style was a risk factor for transgressions-others (p = 0.035). Protective factors for all dependent variables included self-esteem (p<0.001 for associations with PTED, transgressions-self, and betrayal, and p = 0.027 with transgressions-others) and procedural justice (*p's*<0.001). Distributive justice and optimism were protective

For peer review only - http://bmjopen.bmj.com/site/about/guidelines.xhtml

factors of PTED (distributive justice $p = 0.009$, optimism $p<.001$) and of betrayal (distributive justice $p = 0.002$, optimism $p = 0.015$). All dependent variables were influenced by social desirability (*p's* from <0.001 to 0.032, see Figure 1 and Supplementary table 1). All effect sizes were below the RPME or were small sized.

For peer review only

For peer review only - http://bmjopen.bmj.com/site/about/guidelines.xhtml

Table 1.
PTED prevalence, and frequency of endorsement of items on the MIES in UK health and social care workers

PTED scale		
**Cut off score**	**Raw frequency (N)**	**% (95% CI)**
1.6	121	30.3% (25.8 to 35%)
2	77	19.3% (15.5 to 23.5%)
2.5	30	7.5% (5.1 to 10.5%)
**MIES scale**		
Any scale item	289	72.3% (67.6 to 76.6%)
Transgressions by others subscale:	213	53.3% (48.2 to 58.2%)
Transgressions by self subscale	131	32.8% (28.2 to 37.6%
Betrayal subscale	227	56.8% (51.7 to 61.7%)

PTED prevalence was calculated by percentage scoring above a mean total score of 1.6, 2 and 2.5. Each MIES item was coded as endorsed if the participant responded either "slightly agree" "moderately agree" or "strongly agree". Subscale prevalence was calculated by endorsement of any item within that subscale.

Table 2.
Descriptive statistics, normality estimates and Cronbach's alpha estimates

	**Mean ±SD**	**Skew**	**Kurtosis**	**Cronbach's α**
Age	36.84 ±10.73	0.73	-0.22	
Years$_a$	4.50 (2, 9)	2.10	5.14	
Social Desirability	6.93 ±2.80	-0.07	-0.50	0.71
PTED	1.12 ±0.90	0.45	-0.75	0.96
Transgressions-others	2.98 ±1.48	0.16	-1.16	0.78
Transgressions-self	2.17 ±1.22	0.80	-0.37	0.93

For peer review only - http://bmjopen.bmj.com/site/about/guidelines.xhtml

Betrayal	2.77 ±1.38	0.35	-0.93	0.80
Optimism	6.60 ±2.72	-0.46	-0.39	0.83
Pessimism	5.82 ±2.82	0.16	-0.61	0.84
Resilient coping style	14.60 ±2.40	-0.50	0.94	0.62
Distributive justice	18.75 ±4.70	-0.63	-0.13	0.89
Procedural justice	20.33 ±4.77	-0.98	0.84	0.95
Occupational stressors	9.91 ±3.44	0.71	-0.06	0.76
CFC-I	23.47 ±7.68	0.19	-0.61	0.85
CFC-F	34.58 ±6.85	-0.50	0.41	0.84
Self-esteem	28.18 ±5.80	-0.25	-0.21	0.91

$_a$ Median/IQR (Q1, Q3) reported due to non-normality.

PTED = post-traumatic embitterment disorder; transgressions-others = Moral injury transgression by others; transgressions-self = Moral injury transgression by self; betrayal = Moral injury betrayal; CFC-I = Consideration of future consequences-Immediate; CFC-F = Consideration of future consequences-Future.

For peer review only

For peer review only - http://bmjopen.bmj.com/site/about/guidelines.xhtml

Hierarchical multiple regressions.

All variables from Univariate (unadjusted) analyses were included in hierarchical regressions, except for gender and ethnicity, which were not significantly associated with any outcome variables (see Table 3 for regression model summary and supplementary Tables 3 and 4 for regression coefficients).

The full model including demographics, occupational stressors, and personality characteristics explained between 38% and 22% of the variance in PTED and MIES subscales scores (adjusted R^2 = 35% to 18%). This represents a large effect for PTED ($f^2 \geq 0.35$) and moderate effect sizes for all MIES regressions ($f^2 \geq 0.15$). Demographics significantly explained 10% of the variance in PTED, transgressions-self, and transgressions-others scores ($p<0.05$), exposure to occupational stressors in step two significantly explained a further 8-16% of variances in all four regressions ($p<0.001$), and personality characteristics in step three significantly explained a further 6-12% of variance in all four regressions ($p<0.001$), with small to moderate effect sizes (f^2's of 0.05 to 0.21).

Statistically significant risk factors for PTED included being in a clinical support role compared to an allied health ($p = 0.036$) or primary care practitioner ($p = 0.030$), having current mental health diagnoses ($p = 0.006$), exposure to occupational stressors ($p = 0.006$), CFC-I ($p = 0.012$), CFC-F ($p = 0.036$), and distributive justice ($p = 0.024$). Statistically significant protective factors included belief in procedural justice and self-esteem (*p's* = 0.006).

Statistically significant risk factors for transgressions-others included being in a clinical support role compared with allied health professionals ($p = 0.020$), exposure to occupational stressors ($p = 0.002$), and resilient coping style ($p = 0.014$). The only statistically significant protective factor was belief in procedural justice ($p = 0.002$).

For peer review only - http://bmjopen.bmj.com/site/about/guidelines.xhtml

Statistically significant risk factors for transgressions-self included being in a clinical support role compared with an allied health ($p = 0.002$) and managerial role ($p = 0.028$), exposure to occupational stressors ($p = 0.002$), and CFC-I ($p = 0.037$). Statistically significant protective factors included belief in procedural justice ($p = 0.002$) and self-esteem ($p = 0.016$).

Statistically significant risk factors for betrayal were being in a clinical support role compared to an allied health role ($p = 0.036$) and exposure to occupational stressors ($p = 0.006$), whereas belief in procedural justice was a statistically significant protective factor ($p = 0.006$). Social desirability was significantly associated with lower reporting of betrayal ($p = 0.012$) and transgressions-self ($p = 0.002$).

Overall, predictors with βs of practical significance (≥0.20) included exposure to occupational stressors as a risk factor and belief in procedural justice as a protective factor for PTED and all MIES sub-scales, self-esteem as a protective factor for PTED, and social desirability resulted in lower transgressions-self scores (see supplementary Table 3). When running each hierarchical regression with only significant variables to improve model precision and parsimony, each full model explained approximately the same amount of variance in PTED and MIES scores ($R^2 = 19\%$ to 37%, see Table 3). Managerial role ($p = 0.064$) and self-esteem ($p = 0.427$) become non-significant in the transgressions-self model (see supplementary Table 4).

For peer review only - http://bmjopen.bmj.com/site/about/guidelines.xhtml

Table 3.

Model summary of hierarchical regression analyses using all possible predictors and only significant predictors of PTED, transgressions-others, transgressions-self, and betrayal scores with 95% bias corrected and accelerated confidence intervals (2000 samples) (N=394 to 396).

Model including **all possible predictors**				
	PTED $_a$	Transgressions-others $_a$	Transgressions-self $_a$	Betrayal $_a$
Step one – Controls	0.10***	0.04	0.10***	0.05*
Step two – Occupational stressors	0.26/0.16***	0.16/0.12***	0.17/0.08***	0.15/0.10***
Step three – Personality	0.38/0.12***	0.22/0.06***	0.24/0.07***	0.26/0.10***

Model including **only significant predictors** from the previous model				
	PTED $_a$	Transgressions-others $_a$	Transgressions-self $_b$	Betrayal $_b$
	$R^2/\Delta R$	$R^2/\Delta R$	$R^2/\Delta R$	$R^2/\Delta R$
Step one – Controls	0.08***	0.02	0.10***	0.04*
Step two – Occupational stressors	0.24/0.16***	0.13/0.14***	0.17/0.08***	0.14/0.10***
Step three – Personality	0.37/0.13***	0.19/0.04***	0.21/0.04***	0.24/0.10***

*p<0.05, **p<0.01, ***p<0.001

PTED = post-traumatic embitterment disorder; transgressions-others = Moral injury transgression by others; transgressions-self = Moral injury transgression by self; betrayal = Moral injury betrayal.

$_a$ *N*=396

$_b$ *N*=394

For peer review only

Discussion:

This is the first study to show that both PTED and morally injurious events were prevalent in UK HSCWs during September-October 2020 of the COVID-19 pandemic. Correlations show that PTED and morally injurious events are distinct but related constructs and are significantly associated with demographic, occupational, and personality variables. Hierarchical regressions show that occupational stressors were a key risk factor, and personal belief in a procedurally just world, a key protective factor for both conditions. To a lesser extent, being in a clinical support role was a risk factor for both PTED, and exposure to morally injurious events. Beyond this, risk and protective factors have more selective effects. For example, in the final regression models, risk factors for PTED included having current self-reported mental health diagnoses, higher consideration of the immediate and future consequences of actions, and higher levels of personal belief in distributive justice. Self-esteem was a resiliency factor. Higher consideration of the immediate consequences of actions was a risk factor for transgressions-self, and higher levels of resilient coping style was a risk factor for transgressions-others. Social desirability was associated with significantly lower reporting of transgressions-self and betrayal, though effect sizes indicate these selective effects were less practically meaningful.

Our study has several strengths and limitations. This study is novel, providing the first preliminary evidence of both morally injurious events and PTED in UK HSCWs during the COVID-19 pandemic. The results are also generalisable. The survey extends research on HCWs by being open to all UK HSCWs on Prolific, and sample characteristics were broadly similar to the wider NHS workforce in terms of gender (female 76%, NHS = 77%) and ethnicity given that we had missing data (White = 63%, Ethnic minorities = 13.3%, NHS White = 79.2% vs Ethnic

For peer review only - http://bmjopen.bmj.com/site/about/guidelines.xhtml

minorities = 20.7%). The sample included diverse job roles, and 25% of the sample self-reported current mental health diagnoses which matches the general population. The most notable limitation is that cross-sectional baseline data were analysed, and only future analyses from our longitudinal study can establish causal relationships. Data were also collected several months after the pandemic began, no comparisons to pre-pandemic levels of PTED and MIEs were possible, and despite controlling for social desirability, surveys suffer from response and social desirability bias.

Despite these limitations, our study supports early findings that healthcare staff believe themselves to have been exposed to MIEs during the pandemic.[14] Prevalence in this sample is higher than that reported in US HCWs during COVID-19,[12] and is similar to those reported by active-duty US marines.[18] Prevalence of PTED in this sample is eight times higher than a general population sample,[11] but is lower than reported by UK NHS staff attending an occupational health centre pre-pandemic.[31] This indicates that healthcare staff experiencing PTED may be at a higher risk of requiring occupational health services. Further, despite similarities in aetiology and symptomology, studies have rarely examined the overlap between PTED and moral injury. Moderate correlations provide early support to our argument that MIEs can trigger PTED in some individuals and future longitudinal analyses will investigate this model.

Importantly, MIEs and PTED shared the same key risk and protective factors. Experiencing MIEs and PTED was more likely in clinical support workers and those experiencing occupational stressors. Exposure to occupational stressors likely increases the frequency of MIEs that can cause embitterment. Personal belief in procedural justice, which is the belief that you experience fair processes protected against exposure to MIEs and PTED.

For peer review only - http://bmjopen.bmj.com/site/about/guidelines.xhtml
For peer review only

Whilst this belief is generally seen as a disposition that can protect against the negative impact of injustices, it is equally possible that belief in a just world can be modified by exposure to injustices, leading to PTED.

Other vulnerability and resiliency factors vary. Having current mental health diagnoses increased the risk of PTED, and mental health conditions are often comorbid. Considering immediate and future consequences of actions increased risk of PTED, and this may be explained by feelings of helplessness when thinking about the unjust life event, as noted in diagnostic criterion.[10, 11] Belief in distributive justice, which is the belief that you experience fair outcomes increased risk of PTED in contrast to findings that procedural justice was a resiliency factor. Surprisingly, resilient coping style increased exposure to transgressions by others, despite findings that resilient coping tendencies are linked with better psychological well-being.[23] It is possible that people feel more comfortable committing moral transgressions around individuals that they consider to be resilient. Self-esteem increased resilience to PTED, and reliably buffers against poor mental health in the literature.[22] Combined, these findings indicate several vulnerability and resiliency factors that either increase or decrease the risk of exposure to MIEs, and the mental health impact in the form of PTED. Having said that, regressions show these variables only partially predict PTED and morally injurious experiences (19 – 37%) and so support for theoretical models highlighting their importance is limited.[8, 15, 16]

These findings allow for several recommendations. First, some individuals may recover from moral injuries and feelings of embitterment on their own, but others will need psychological support and clinicians should be aware that these individuals are relatively treatment resistant and have different treatment needs.[8,10,11] Self-care strategies should focus

For peer review only - http://bmjopen.bmj.com/site/about/guidelines.xhtml

on increasing self-esteem and reducing tendencies to think about the consequences of one's actions, in line with this study's findings. More importantly though, there should be an emphasis not on the individual to 'recover' but on healthcare leaders to tackle systemic issues that are causing moral injuries and embitterment in the first place. In this study, staff across all levels of health and social care have reported feeling betrayed, morally violated, and that they had been treated unjustly and unfairly during the pandemic. A proportion of these responses have been directly linked to the working environment (e.g., lack of PPE and resources). Healthcare authorities should strive to maintain fair 'processes' even if reducing all workplace stressors is unachievable. At a time when the UK and other countries need a healthy workforce to respond to the pandemic and its aftermath, staff retention is a priority.

To strengthen prevalence estimates reported here, large scale cohort studies using randomly selected, representative samples are needed. Follow up data collection with these samples can establish whether prevalence estimates increase across time as the pandemic continues. Future studies should identify the cognitive, affective, and behavioural manifestations of PTED and moral injury in healthcare professionals to inform treatment and treatment efficacy then needs to be established. Although beyond the scope of this paper, future studies should investigate the overlap between PTED and moral injury symptoms using newly developed symptom scales, and their overlap with other similar constructs like burnout.[9]

In conclusion, we are among the first to provide prevalence estimates of exposure to MIEs and PTED in health and social care workers as a direct result of working during the COVID-19 pandemic. We provide evidence that those who are clinical support staff, exposed to occupational stressors, and have a lack of belief that they experienced fair processes are amongst the most at risk. These findings indicate that measures to reduce workplace stressors and

For peer review only - http://bmjopen.bmj.com/site/about/guidelines.xhtml

ensuring staff feel they receive fair procedures at work should be a priority of healthcare organisations.

Figure 1.

Heatmap showing Pearson's correlation coefficients of study variables.

For peer review only

For peer review only - http://bmjopen.bmj.com/site/about/guidelines.xhtml

Contributor and guarantor information:

CB designed the project and data collection tools, collected the data, cleaned, analysed and interpreted the data and drafted and revised the paper. She is guarantor. MM analysed and interpreted the data and revised the paper. JC designed the project and data collection tools, interpreted the data, and revised the paper. All authors gave final approval for the publication of this manuscript. The corresponding author attests that all listed authors meet authorship criteria and that no others meeting the criteria have been omitted.

Copyright:

The Corresponding Author has the right to grant on behalf of all authors and does grant on behalf of all authors, an exclusive licence (or non-exclusive for government employees) on a worldwide basis to the BMJ Publishing Group Ltd to permit this article (if accepted) to be published in BMJ editions and any other BMJPGL products and sublicences such use and exploit all subsidiary rights, as set out in our licence.

Competing interest statement:

All authors have completed the Unified Competing Interest form (available on request from the corresponding author) and declare: the lead author had funding support from the Economic and Social Research Council and University of Liverpool COVID-19 strategic funding for the submitted work; no financial relationships with any organisations that might have an interest in the submitted work in the previous three years; no other relationships or activities that could appear to have influenced the submitted work.

Funding:

For peer review only - http://bmjopen.bmj.com/site/about/guidelines.xhtml

CB was supported by the Economic and Social Research Council UK [ES/P000665/1] and this work received COVID-19 strategic funding from the University of Liverpool.

Role of sponsor:

The study sponsors did not play a role in the study design, management, data collection, analyses, in the interpretation of the data or in the writing of the manuscript or the decision to submit for publication.

Transparency declaration:

The lead author affirms that this manuscript is an honest, accurate, and transparent account of the study being reported; that no important aspects of the study have been omitted; and that any discrepancies from the study as planned have been explained.

Ethical approval:

Ethical approval for the study was granted by the University of Liverpool (reference: 7862) and all participants provided informed consent.

Data availability statement:

Data from this study will be made available upon reasonable request by contacting the corresponding author at hlcbrenn@liverpool.ac.uk. Data will be made available after an embargoed period of 12 months to permit planned analyses of the longitudinal dataset and will be available for 10 years in line with ethical considerations.

Dissemination declaration:

Results from this study will be disseminated to participants that request this feedback and will be disseminated to relevant audiences through University of Liverpool communications.

For peer review only

References:

1. John Hopkins University (JHU). Mortality Analyses–Coronavirus Resource Centre. JHU, 2020 (https://coronavirus.jhu.edu/data/mortality).

2. NHS England. NHS staff survey. https://www.nhsstaffsurveyresults.com (Accessed 5 Feb 2020).

3. De Kock JH, Latham HA, Leslie SJ, Grindle M, Munoz S-A, Ellis L, et al. A rapid review of the impact of COVID-19 on the mental health of healthcare workers: implications for supporting psychological well-being. *BMC Public Health* 2021;21:104. DOI: https://doi.org/10.1186/s12889-020-10070-3

4. Major A, Hlubocky FJ. Mental health of health care workers during the COVID-19 pandemic and evidence-based frameworks for mitigation: A rapid review. *medRxiv* 2021.01.03.21249166 [Preprint]. January 04, 2021 [cited 2021 Feb 5] DOI: https://doi.org/10.1101/2021.01.03.21249166.

5. Greene T, Harju-Seppänen J, Adeniji M, Steel C, Grey N, Brewin CR et al. Predictors and rates of PTSD, depression and anxiety in UK frontline health and social care workers during COVID-19. European Journal of Psychotraumatology 2021;12. DOI: 10.1080/20008198.2021.1882781

6. Greenberg N, Docherty M, Gnanapragasam S, Wessely S. Managing mental health challenges faced by healthcare workers during covid-19 pandemic. *BMJ* 2020;368:m1211. doi:10.1136/bmj.m1211

For peer review only - http://bmjopen.bmj.com/site/about/guidelines.xhtml

For peer review only

7. British Medical Association COVID-19 survey December 2020. https://www.bma.org.uk/media/3637/bma-covid-survey-results-all-doctors-dec-2020.pdf (Accessed 5 April 2021).

8. Litz BT, Stein N, Delaney E, Lebowitz L, Nash WP, Silva C, et al. Moral injury and moral repair in war veterans: A preliminary model and intervention strategy. *Clinical Psychology Review* 2009;29:695-706. DOI: https://doi.org/10.1016/j.cpr.2009.07.003

9. Dean W, Talbot S, Dean A. Reframing Clinician Distress: Moral Injury Not Burnout. Fed Pract. 2019;36(9):400-2.

10. Linden M, Baumann K, Rotter M, Schippan B. Diagnostic criteria and the standardized diagnostic interview for posttraumatic embitterment disorder (PTED). International Journal of Psychiatry in Clinical Practice. 2008;12(2):93-6. DOI: https://doi.org/10.1080/13651500701580478

11. Linden M, Baumann K, Lieberei B, Rotter M. The post-traumatic embitterment disorder Self-Rating Scale (PTED Scale). Clinical Psychology & Psychotherapy 2009;16:139-47.

12. Hines SE, Chin KH, Levine AR, Wickwire EM. Initiation of a survey of healthcare worker distress and moral injury at the onset of the COVID-19 surge. *Am J Ind Med* 2020;63:830-3. DOI: https://doi.org/10.1002/cpp.610

13. Zhizhong W, Koenig HG, Yan T, Jing W, Mu S, Hongyu L, et al. Psychometric properties of the moral injury symptom scale among Chinese health professionals during the COVID-19 pandemic. *BMC Psychiatry* 2020;20:556. DOI: https://doi.org/10.1186/s12888-020-02954-w

14. Lamb D, Gnanapragasam S, Greenberg N, Bhundia R, Carr E, Hotopf M et al. The psychosocial impact of COVID-19 pandemic on 4,378 UK healthcare workers and ancillary

For peer review only - http://bmjopen.bmj.com/site/about/guidelines.xhtml

staff: initial baseline data from a cohort study collected during the first wave of the pandemic. *medRxiv* 2021.01.03.21249166 [Preprint]. January 22, 2021 [cited 2021 March 23]

15. Dodek A, Barnow S. Personality as risk and resilience in embitterment reactions. In: Linden M, Maercker A, editors. Embitterment: Societal, psychological, and clinical perspectives. Vienna: Springer Vienna; 2011. p. 83-100.

16. McKay MT, Perry JL, Percy A, Cole JC. Evidence for the reliability and validity, and some support for the practical utility of the two-factor Consideration of Future Consequences Scale-14. *Personality and Individual Differences* 2016;98:133-136. https://doi.org/10.1016/j.paid.2016.03.097

17. National Health Service (NHS). Explore roles. Available from: https://www.healthcareers.nhs.uk/explore-roles. (Accessed 7 March 2022)

18. Nash WP, Marino Carper TL, Mills MA, Au T, Goldsmith A, Litz BT. Psychometric Evaluation of the Moral Injury Events Scale. Military Medicine 2013;178:646-52. DOI: https://doi.org/10.7205/MILMED-D-13-00017

19. Bryan CJ, Bryan AO, Anestis MD, Anestis JC, Green BA, Etienne N, et al. Measuring moral injury: Psychometric properties of the moral injury events scale in two military samples. Assessment 2016;23:557-70. DOI: https://doi.org/10.1177/1073191115590855

20. Scheier MF, Carver CS, Bridges MW. Distinguishing optimism from neuroticism (and trait anxiety, self-mastery, and self-esteem): a reevaluation of the Life Orientation Test. J Pers Soc Psychol 1994;67:1063-78. DOI: https://doi.org/10.1037/0022-3514.67.6.1063

For peer review only - http://bmjopen.bmj.com/site/about/guidelines.xhtml

21. Glaesmer H, Rief W, Martin A, et al. Psychometric properties and population-based norms of the Life Orientation Test Revised (LOT-R). Br. J. Health. Psychol. 2012;17;432-445. DOI: https://doi.org/10.1111/j.2044-8287.2011.02046.x

22. Rosenberg, M. (1965). Society and the adolescent self-image. Princeton, NJ: Princeton University Press

23. Sinclair VG, Wallston KA. The development and psychometric evaluation of the Brief Resilient Coping Scale. Assessment 2004;11:94-101. DOI: https://doi.org/10.1177/1073191103258144

24. Lucas T, Zhdanova L, Alexander S. Procedural and Distributive Justice Beliefs for Self and Others Assessment of a Four-Factor Individual Differences Model. Journal of Individual Differences 2011;32:14-25. DOI: https://doi.org/10.1027/1614-0001/a000032

25. Strathman A, Gleicher F, Boninger DS, Edwards CS. The consideration of future consequences: weighing immediate and distant outcomes of behavior. J Pers Soc Psychol 1994;66:742. DOI: https://doi.org/10.1037/0022-3514.66.4.742

26. Crowne DP, Marlowe D. A new scale of social desirability independent of psychopathology. Journal of consulting psychology 1960;24:349. DOI: https://doi.org/10.1037/h0047358

27. Ferguson CJ. An effect size primer: a guide for clinicians and researchers. Professional Psychology: Research and Practice 2016;5:532-538. DOI: https://doi.org/10.1037/14805-020

28. Cohen J. (1988). Statistical Power Analysis for the Behavioral Sciences (2nd ed.). Routledge. DOI: https://doi.org/10.4324/9780203771587

29. Perry J, Nicholls A, Clough P, Crust L. 2015. Assessing model fit: Caveats and recommendations for confirmatory factor analysis and exploratory structural equation

For peer review only - http://bmjopen.bmj.com/site/about/guidelines.xhtml

modelling. Measurement in Physical Education and Exercise Science 2015;19:12–21. https://doi.org/10.1080/1091367X.2014.952370

30. Little RJA. A test of missing completely at random for multivariate data with missing values. Journal of the American Statistical Association 1988;83:1198–1202

31. Sensky T, Salimu R, Ballard J, Pereira D. Associations of chronic embitterment among NHS staff. Occupational Medicine 2015;65;431-436. DOI: https://doi.org/10.1093/occmed/kqv089

For peer review only

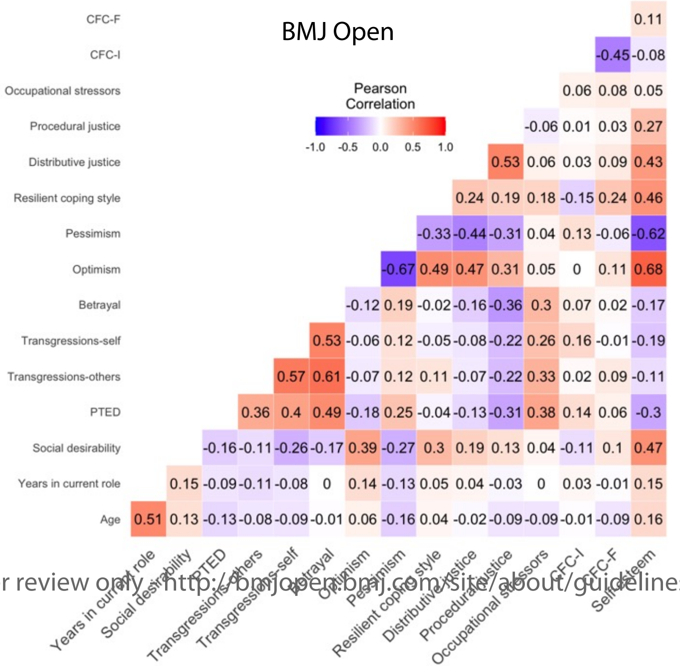

For peer review only - http://bmjopen.bmj.com/site/about/guidelines.xhtml

Supplementary tables

Supplementary table 1.

Socio-demographic characteristics of participants, and their association with PTED and the MIES subscales (95% CI with 2,000 bootstrapped samples)

	N	%	PTED	*p*	Effect size	Transgressions-others	*p*	Effect size	Transgressions-self	*p*	Effect size	Betrayal	*p*	Effect size
**Gender**[a]														
Male	98	24.5%	1.17 ±1.0	0.493	0.08	3.07 ±1.53	0.484	0.08	2.21 ±1.17	0.668	0.05	2.86 ±1.43	0.450	0.09
Female	302	75.5%	1.10 ±0.87			2.95 ±1.47			2.15 ±1.24			2.74 ±1.36		
**Professional role**[b]														
Allied health	58	14.5%	0.96 ±0.92	0.215	0.01	2.71 ±1.44	0.374	0.00	1.90 ±1.05	0.021[c]	0.04	2.55 ±1.41	0.589	0.00
Primary care practitioner	63	15.8%	1.04 ±0.91			3.07 ±1.45			2.32 ±1.27			2.87 ±1.42		
Managerial	41	10.3%	0.96 ±0.87			2.66 ±1.58			1.84 ±1.25			2.60 ±1.63		
Health info/science	37	9.3%	1.38 ±1			3.18 ±1.53			2.40 ±1.27			2.89 ±1.43		
Psychological	27	6.8%	1.11 ±0.75			2.83 ±1.32			1.79 ±.98			2.56 ±1.08		
Corporate	65	16.3%	1.07 ±0.78			3.08 ±1.48			2.13 ±1.09			2.81 ±1.38		
Clinical support	105	26.3%	1.23 ±0.95			3.14 ±1.50			2.43 ±1.29			2.92 ±1.30		
**Mental health**[a]														
Current mental health diagnoses	97	24.3%	1.52 ±0.96	<0.001[d]	0.64	3.20 ±1.57	0.100	0.20	2.31 ±1.30	0.177	0.15	3.10 ±1.40	0.013[e]	0.31
No current mental health diagnoses	303	75.8%	0.98 ±0.84			2.91 ±1.45			2.12 ±1.20			2.68 ±1.36		
**Ethnicity**[a]														
White	252	63%	1.09 ±0.90	0.628	0.07	2.93 ±1.54	0.390	0.13	2.08 ±1.20	0.124	0.23	2.77 ±1.38	0.951	0.01

For peer review only

Ethnic minorities	53	13.3%	1.15 ±0.93	3.13 ±1.38	2.36 ±1.16	2.76 ±1.40

Mean ±SD are reported. a Independent samples t-test reporting Cohen's *d* effect size, b One-way ANOVA reporting ω^2 effect size, c Welch's *F*(6, 138.418) = 2.579, *p*=0.021, d Mean difference -0.54 BCA 95% CI [-0.74, -0.34] t(398) = -5.33, p<0.001, e Mean difference -0.40 BCA 95% CI [-0.72, -0.07] t(293) = -2.50, p=0.013[1]

[1] Non-parametric Mann Whitney-U and Kruskal Wallis-H tests were also conducted. There were no differences in findings.

For peer review only

Supplementary table 2.

Severity and frequency of endorsement of items on the MIES and PTED scale in UK health and social care workers

MIES Item-level endorsement	Raw frequency (N)	% (95% CI)
I saw things that were morally wrong	179	44.8% (39.8 to 49.8%)
I am troubled by having witnessed others immoral acts	167	41.8% (36.9 to 46.8%)
I acted in ways that violated by own moral code or values	76	19% (15.3 to 23.2%)
I am troubled by having acted in ways that violated by own moral code or values	94	23.5% (19.4 to 28%)
I violated by own morals by failing to do something that I felt I should have done	83	20.8% (16.9 to 25.1%)
I am troubled because I violated by own morals by failing to do something that I felt I should have done	78	19.5% (15.7 to 23.7%)
I feel betrayed by superiors who I once trusted.	161	40.3% (35.4 to 45.2%)
I feel betrayed by fellow colleagues who I once trusted.	124	31% (26.5 to 35.8%)
I feel betrayed by others outside my work organisation who I once trusted.	158	39.5% (34.7 to 44.5%)
**PTED Item-level endorsement**	**Raw frequency (N)**	**% (95% CI)**
Any item endorsed by at "partially true" or greater level	293	73.3% (68.6 to 77.5%)
that hurt my feelings and caused considerable embitterment	142	35.5% (30.8 to 40.4%)
that led to a noticeable and persistent negative change in my mental well-being	167	41.8% (36.9 to 46.8%)
that I see as very unjust and unfair	170	42.5% (41.8 to 36.9%)
about which I have to think over and over again	167	41.8% (35.8 to 31%)
that causes me to be extremely upset when I am reminded of it	143	35.8% (31 to 40.7%)
that triggers me to harbour thoughts of revenge	44	11% (8.1 to 14.5%)
for which I blame and am angry with myself	87	21.8% (17.8 to 26.1%)
that led to the feeling that there is no sense to strive to make an effort	119	29.8% (25.3 to 34.5)
that makes me to frequently feel sullen or unhappy	151	37.8% (33 to 42.7%)
that impaired my overall physical well being	138	34.5% (29.8 to 39.4%)
that causes me to avoid certain places or persons so as to not be reminded of them	120	30% (25.5 to 34.8%)
that makes me feel helpless and disempowered	141	40.5% (35.7 to 45.5%)

For peer review only

that triggers feelings of satisfaction when I think that the responsible party having to experience a similar situation	76	19% (15.3 to 23.2%)
that led to a considerable decrease in my strength and drive	164	41% (36.1 to 46%)
that made that I am more easily irritated than before	176	44% (39.1 to 49%)
that makes that I must distract myself in order to experience a normal mood	160	40% (35.2 to 45%)
that made me unable to pursue occupational and/or family activities as before	176	44% (39.1 to 49%)
that caused me to draw back from friends and social activities	179	44.8% (39.8 to 49.8%)
which frequently evokes painful memories	118	29.5% (25.1 to 34.2%)

For item-level endorsement, each MIES item was coded as endorsed if the participant responded either “slightly agree” “moderately agree” or “strongly agree” and each PTED item was coded as endorsed if the participant responded either “partially true” “very much true” or “extremely true”.

For peer review only

Supplementary table 3.

Coefficients from the final step in hierarchical multiple regression analyses predicting PTED and transgressions-others, transgressions-self, and betrayal scores with 95% bias corrected and accelerated confidence intervals (2000 samples) (N=394).

	PTED			Transgressions-others			Transgressions-self			Betrayal		
Predictors	β	*B* 95% CI	*p*	β	*B* 95% CI	*p*	β	*B* 95% CI	*p*	β	*B* 95% CI	*p*
Age	-0.01	(-0.01 to 0.01)	0.789	0.04	(-0.01 to 0.02)	0.555	0.03	(-0.01 to 0.02)	0.587	0.04	(-0.01 to 0.02)	0.392
Current mental health diagnoses	0.12	(0.07 to 0.46)	**0.006**	-0.01	(-0.43 to 0.28)	0.835	-0.04	(-0.39 to 0.18)	0.512	0.04	(-0.25 to 0.45)	0.404
Social desirability	-0.02	(-0.03 to 0.02)	0.729	-0.08	(-0.10 to 0.01)	0.128	-0.21	(-0.14 to -0.05)	**0.002**	-0.13	(-0.11 to -0.01)	**0.012**
Years in role	-0.02	(-0.02 to 0.01)	0.747	-0.10	(-0.05 to 0.01)	0.110	-0.08	(-0.04 to 0.01)	0.185	0.01	(-0.02 to 0.02)	0.922
Allied	-0.12	(-0.60 to -0.09)	**0.036**	-0.12	(-0.93 to -0.09)	**0.020**	-0.16	(-0.87 to -0.15)	**0.002**	-0.10	(-0.71 to 0.00)	**0.036**
Primary care practitioner	-0.11	(-0.54 to -0.04)	**0.030**	-0.07	(-0.72 to 0.14)	0.201	-0.09	(-0.70 to 0.12)	0.185	-0.05	(-0.69 to 0.20)	0.434
Managerial	-0.04	(-0.37 to 0.15)	0.398	-0.11	(-1.12 to 0.04)	0.077	-0.13	(-0.91 to -0.03)	**0.028**	-0.04	(-0.64 to 0.39)	0.476
Health info/science	0.06	(-0.10 to 0.46)	0.157	0.03	(-0.40 to 0.69)	0.573	-0.01	(-0.47 to 0.43)	0.939	0.01	(-0.43 to 0.57)	0.819
Psychological	0.04	(-0.12 to 0.50)	0.307	-0.02	(-0.67 to 0.37)	0.581	-0.09	(-0.92 to 0.04)	0.061	-0.01	(-0.47 to 0.46)	0.934
Corporate	-0.02	(-0.29 to 0.21)	0.681	0.00	(-0.49 to 0.42)	0.978	-0.08	(-0.58 to 0.09)	0.142	-0.02	(-0.43 to 0.38)	0.747
Occupational stressors	0.38	(0.08 to 0.12)	**0.006**	0.32	(0.10 to 0.18)	**0.002**	0.26	(0.06 to 0.13)	**0.002**	0.29	(0.08 to 0.15)	**0.006**
Optimism	0.03	(-0.04 to 0.06)	0.681	-0.01	(-0.09 to 0.08)	0.876	0.15	(-0.01 to 0.13)	0.073	.007	(-0.04 to 0.14)	0.386
Pessimism	-0.01	(-0.05 to 0.05)	0.843	0.01	(-0.06 to 0.08)	0.888	-0.04	(-0.08 to 0.05)	0.644	.005	(-0.03 to 0.10)	0.470
Resilient coping style	0.06	(-0.01 to 0.06)	0.265	0.15	(0.03 to 0.16)	**0.014**	0.02	(-0.05 to 0.06)	0.746	.005	(-0.03 to 0.10)	0.410
Distributive justice	0.13	(0.01 to 0.04)	**0.024**	0.08	(-0.01 to 0.06)	0.163	0.08	(-0.01 to 0.05)	0.220	.007	(-0.01 to 0.06)	0.217
Procedural justice	-0.29	(-0.07 to -0.04)	**0.006**	-0.23	(-0.11 to -0.03)	**0.002**	-0.23	(-0.09 to -0.03)	**0.002**	-0.35	(-0.13 to -0.07)	**0.006**

For peer review only

CFC-I	0.14	(0.01 to 0.03)	**0.012**	0.03	(-0.02 to 0.03)	0.565	0.12	(0.01 to 0.04)	**0.037**	0.05	(-0.01 to 0.03)	0.355
CFC-F	0.11	(0.01 to 0.03)	**0.036**	0.08	(-0.01 to 0.04)	0.130	0.07	(-0.01 to 0.03)	0.262	0.05	(-0.01 to -0.03)	0.331
Self-esteem	-0.28	(-.07 to -.02)	**0.006**	-0.09	(-0.07 to 0.01)	0.207	-0.19	(-0.07 to -0.01)	**0.016**	-0.09	(-0.06 to 0.02)	0.241

Reference groups: Current mental health diagnoses (none), Professional role (clinical support role)
PTED = post-traumatic embitterment disorder; transgressions-others = Moral injury transgression by others; transgressions-self = Moral injury transgression by self; betrayal = Moral injury betrayal; CFC-I = Consideration of future consequences-Immediate; CFC-F = Consideration of future consequences-Future.
Significant p values are in bold.

For peer review only

Supplementary table 4.

Coefficients from the final step in hierarchical regression analyses using ***only significant predictors*** *of PTED, transgressions-others, transgressions-self, and betrayal scores with 95% bias corrected and accelerated confidence intervals (2000 samples) (N=394 to 396).*

PTED [a]				Transgressions-others [a]				Transgressions-self [b]				Betrayal [b]			
Predictors	β	*B* 95% CI	*p*	Predictors	β	*B* 95% CI	*p*	Predictors	β	*B* 95% CI	*p*	Predictors	β	*B* 95% CI	*p*
Current mental health diagnoses	0.12	(0.06 to 0.50)	**0.006**	Allied	-0.13	(-0.94 to -0.13)	**0.018**	Allied	-0.15	(-0.82 to -0.21)	**0.005**	Allied	-0.10	(-0.78 to 0.01)	**0.043**
Allied	-0.12	(-0.55 to -0.03)	**0.019**	Primary care practitioner	-0.07	(-0.77 to 0.19)	0.206	Primary care practitioner	-0.08	(-0.63 to 0.12)	0.190	Primary care practitioner	-0.05	(-0.63 to 0.23)	0.317
Primary care practitioner	-0.12	(-0.51 to -0.03)	**0.024**	Managerial	-0.10	(-0.99 to 0.10)	0.086	Managerial	-0.10	(-0.84 to 0.03)	0.064	Managerial	-0.05	(-0.66 to 0.29)	0.385
Managerial	-0.03	(-0.35 to 0.13)	0.466	Health info/science	0.04	(-0.30 to 0.77)	0.369	Health info/science	0.01	(-0.44 to 0.46)	0.991	Health info/science	0.01	(-0.38 to 0.44)	0.882
Health info/science	0.06	(-0.07 to 0.50)	0.188	Psychological	0.01	(-0.49 to 0.48)	0.976	Psychological	-0.08	(-0.85 to 0.07)	0.080	Psychological	-0.01	(-0.53 to 0.33)	0.724
Psychological	0.04	(-0.16 to 0.45)	0.344	Corporate	0.02	(-0.31 to 0.47)	0.690	Corporate	-0.06	(-0.52 to 0.13)	0.214	Corporate	-0.02	(-0.47 to 0.28)	0.675

Corporate	-0.12	(-0.28 to 0.19)	0.727	Occupational stressors	0.34	(0.11 to 0.19)	**0.001**	Social desirability	-0.20	(-0.13 to 0.04)	**0.001**	Social desirability	-0.14	(-0.11 to -0.02)	**0.007**
Occupational stressors	0.39	(0.08 to 0.12)	**0.002**	Resilient coping style	0.11	(0.01 to 0.12)	**0.021**	Occupational stressors	0.27	(0.06 to 0.14)	**0.001**	Occupational stressors	0.31	(0.09 to 0.16)	**0.001**
Distributive justice	0.14	(0.01 to 0.04)	**0.006**	Procedural justice	-0.21	(-0.10 to -0.04)	**0.001**	Procedural justice	-0.16	(-0.07 to -0.01)	**0.001**	Procedural justice	-0.32	(-0.12 to -0.07)	**0.001**
Procedural justice	-0.28	(-0.07 to -0.03)	**0.002**					CFC-I	0.10	(-0.01 to 0.03)	**0.046**				
CFC-I	0.13	(0.01 to 0.03)	**0.008**					Self-esteem	-0.04	(-0.03 to 0.01)	0.427				
CFC-F	0.12	(0.01 to 0.03)	**0.006**												
Self-esteem	-0.25	(-0.05 to -0.02)	**0.002**												

Reference groups: Current mental health diagnoses (none), Professional role (clinical support role)
PTED = post-traumatic embitterment disorder; transgressions-others = Moral injury transgression by others; transgressions-self = Moral injury transgression by self; betrayal = Moral injury betrayal; CFC-I = Consideration of future consequences-Immediate; CFC-F = Consideration of future consequences-Future.
Significant p values are in bold.
[a] *N*=396
[b] *N*=394

For peer review only

Supplementary information: Confirmatory factor analysis for all study variables

Confirmatory factor analyses (CFA) for all instruments used in the present study were carried out with AMOS 26, and all estimations are based on maximum likelihood procedures. To assess model fit, we examined incremental fit indices of the comparative fit index (CFI) and Tucker-Lewis index (TLI) and the absolute fit indices of root-mean square error of approximation (RMSEA) and standardised root mean square residual (SRMR). In line with recommendations by Perry, Nicholls, Clough, and Crust[29] no arbitrary value was used as a cut-off. Instead, CFI and TLI close to 0.90 and SRMR and RMSEA close to zero (i.e., <0.06) were interpreted as representing adequate model fit.

Moral Injury Events Scale (MIES)

There is ongoing debate regarding model fit for the MIES, with a ‘transgressions’ (items 1-6) and ‘betrayal’ (i.e., items 7-9) two factor model proposed by Nash and colleagues[18], a ‘transgression-other’ (i.e., items 1,2,7,8, and 9) and ‘transgression-self’ two factor model (i.e., items 3-6) proposed by Richardson et al., and a three factor model proposed by Bryan et al.,[19](i.e., transgressions-others [items 1-2], transgressions-self [items 3-6] and betrayal [items 7-9]. We tested all three. Fit indices for Nash’s initial two factor structure was suboptimal: $\chi^2(26) = 211.257$, CFI = .917, p<0.001, TLI = 0.886, SRMR = 0.087, RMSEA (90% CI) = 0.134 (0.117, 0.151). The two-factor structure proposed by Richardson showed an improvement in model fit: $\chi^2(26) = 104.894$, p<0.001, CFI = 0.965, TLI = 0.951, SRMR = 0.041, RMSEA (90% CI) = 0.09 (0.07, 0.11). However, the three-factor solution proposed by Bryan had superior model fit and was used in subsequent analyses: $\chi^2(24) = 36.217$, p=0.052, CFI = 0.995, TLI = 0.992, SRMR = 0.017, RMSEA (90% CI) = 0.036 (0.000, 0.058).

Post-traumatic embitterment scale (PTED)

The PTED scale is employed as a unidimensional scale and fit indices indicated that model fit was permissible despite some measurement error in the absolute fit indices, $\chi^2(152)$

= 617.373, p<0.001, CFI = 0.924, TLI = 0.915, SRMR = 0.044, RMSEA (90% CI) = 0.088 (0.080, 0.095).

Exposure to occupational stressors scale

For this study we developed a measure assessing exposure to occupational stressors. A unidimensional model in which all items loaded onto one latent factor presented a satisfactory model fit: $\chi^2(5) = 35.32$, p<0.001, CFI = 0.944, TLI = 0.934, SRMR = 0.059, RMSEA (90% CI) = 0.051 (0.036, 0.066). This is noteworthy given that this was a newly developed instrument.

Revised Life Orientation Test (LOT-R)

The LOT-R can be used as a unidimensional scale by reverse-scoring pessimism items to generate an overall optimism score. However, Glaesmer et al.,[21] argued for the use of a two-factor model. We tested both and found the unidimensional model fit to be unsatisfactory: $\chi^2(9) = 98.909$, p<0.001 CFI = 0.925, TLI = 0.875, SRMR = 0.055, RMSEA (90% CI) = 0.158 (0.131, 0.187). The two-factor model presented a significant improvement and was used in subsequent analyses: $\chi^2(8) = 23.268$, p=0.003 CFI = 0.987, TLI = 0.976, SRMR = 0.027, RMSEA (90% CI) = 0.069 (0.037, 0.103).

Rosenberg Self-esteem Scale

The Rosenberg self-esteem is primarily employed as a unidimensional scale with negatively worded items reverse scored. The unidimensional scale displayed some measurement error, primarily in the absolute fit indices: $\chi^2(35) = 203.48$, p<0.001, CFI = 0.920, TLI = 0.897, SRMR = 0.049, RMSEA (90% CI) = 0.110 (0.095, 0.125). However, all factor loadings were significant.

Brief Resilience Coping Scale

For peer review only

The unidimensional factor structure of the brief resilient coping scale demonstrated good model fit $\chi^2(2) = 3.243$ p=.198, CFI = .993, TLI = 0.980, SRMR = 0.021, RMSEA (90% CI) = 0.039 (0.000, 0.115).

Personal belief in a just world scale (personal BJW)

According to Lucas, Zhdanova and Alexander[24] the personal BJW is comprised of two factors, distributive justice (distributive justice; items 1-4) and procedural justice (procedural justice; items 5-8). First, we tested a unidimensional model in which all items loaded onto a single latent factor and found this model was poor, $\chi^2(20) = 802.839$, CFI = 0.723, TLI = 0.612, SRMR = 0.164, RMSEA (90% CI) = 0.313 (0.295, 0.332). The two-factor model displayed some measurement error as shown by the absolute fit indices but had much better fit; $\chi^2(19) = 144.221$, CFI = 0.956, TLI = 0.935, SRMR = 0.027, RMSEA (90% CI) = 0.129 (0.109, 0.149). The two sub-scales were used in subsequent analyses.

Consideration of future consequences-14 scale (CFC-14)

Given the on-going question regarding the model fit for the CFC scale[16] we examined a one-factor and a two-factor model. Model fit for a one-factor solution was less than ideal: $\chi^2(77)= 594.219$, $p < 0.001$; CFI = 0.749; TLI = 0.704; RMSEA = 0.130 (95% CI = 0.120, 0.140); SRMR = 0.105. Conversely, fit indices for the two-factor solution were acceptable and used in subsequent analyses: χ^2 (76)=179.026, $p < 0.001$; CFI = 0.950; TLI = 0.940; RMSEA = 0.058 (95% CI = 0.047, 0.069); SRMR = 0.059.

Social Desirability scale

The unidimensional factor structure of the social desirability scale demonstrated some measurement error in the comparative fit indices: $\chi^2(65) = 130.815$, p<0.001, CFI = 0.861, TLI = 0.833, SRMR = 0.051, RMSEA (90% CI) = 0.051 (0.038, 0.163) and item 13 had a relatively poor factor loading (i.e., 0.28). Despite this, all factor loadings did significantly load onto their latent factor.

For peer review only

Checklist for Reporting Results of Internet E-Surveys (CHERRIES)

Checklist Item	***Explanation***	***Page number***
Describe survey design	We conducted a longitudinal voluntary survey, using a purposive convenience sample of UK health care workers who worked during the COVID-19 pandemic. This paper reports the cross-sectional analysis of baseline data.	Page 6/7
IRB approval	Ethical approval was granted by the Ethics Committee at The University of Liverpool.	Page 6
Informed consent	Study information was provided on Prolific, then again on the electronic information sheet. The information sheet detailed study information, the identity of the investigators, the time investment for the survey and that completing, that data would be held for a maximum of ten years, and that data was anonymous. Participants then provided electronic informed consent.	Page 7
Data protection	No personally identifiable information was collected.	Page 7-10
Development and testing	An anonymous electronic questionnaire was designed by the authors. Primarily validated scales from the literature were used.	Page 7-10
Open survey versus closed survey	Data was collected using a closed survey, only individuals registered with Prolific, that met certain eligibility criteria (i.e., fluent in English, located in the UK, and work within health and social care assistance or medical/healthcare industries) were allowed to take part in the survey.	Page 6/7
Contact mode	Initial contact with participants was made on the Internet, using the Prolific website.	Page 6/7
Advertising the survey	The survey was advertised on Prolific, to those meeting pre-defined eligibility criteria (i.e., fluent in English, located in the UK, and work within health and social care assistance or medical/healthcare industries). The advertisement informed participants that the aim of the study was to explore factors influencing the onset of mental health difficulties following the COVID-19 pandemic, and that they would be invited to take part in a second round of data collection in 12 months. They were informed of the eligibility criteria and of the financial incentive to take part, they would receive £2.50 for taking part in survey one, and a further £2.50 for taking part in survey two in 12 months.	Page 7

For peer review only

Web/E-mail	This was an electronic web survey using the Qualtrics platform. Captured responses were automatically recorded into a database.	Page 7
Context	The survey was posted on Prolific. Prolific is an online crowdsourcing platform which holds a database of potential participants to take part in studies. Potential participants have answered demographic questions about themselves, and these can be used to select for certain individuals. Individuals meeting these criteria are then sent the study advertisement by Prolific.	Page 7
Mandatory/voluntary	The survey was completely voluntary.	Page 7
Incentives	Participants received £2.50 for completing the survey in line with platform guidelines.	Page 7
Time/Date	Data was collected from 24th September – 6th October 2020.	Page 6
Randomization of items or questionnaires	To prevent biases, the order of the questionnaires was randomized and the items within the questionnaires were randomized.	Page 7
Adaptive questioning	Adaptive questioning was not used.	Page 7
Number of Items	There were 98 survey items, and number of items per page varied from 1 to 19.	Page 7
Number of screens (pages)	Excluding the information, consent and debrief sheets, the survey consisted of 11 pages.	Page 7
Completeness check	Completeness was checked after survey completion.	Page 7
Review step	There was no review stage where participants could review their answers.	Page 6-7
Unique site visitor	All participants could only complete the survey once, through Prolific. Prolific uses several tracking mechanisms including IP address detection to ensure that no participant takes part in a study more than once.	Page 7
View rate (Ratio of unique survey visitors/unique site visitors)	Only completion rate can be calculated with Prolific.	Page 7
Participation rate (Ratio of unique visitors who agreed to participate/unique first survey page visitors)	Only completion rate can be calculated with Prolific.	Page 7
Completion rate (Ratio of users who finished the	Out of 447 people that agreed to participate, 47 dropped out and did not fully complete the survey. This is a completion rate of 89.49%.	Page 7

For peer review only

survey/users who agreed to participate)		
Cookies used	Not used. Prolific prevents multiple entries from the same individual using IP addresses, The Internet Service Provider (ISP), Device and browser information and by not allowing VPN or proxy usage.	Page 7
IP check	Prolific, which is the website we used for recruitment uses IP addresses to identify potential duplicate entries from the same user. However, we do not have access to information on how exactly they use this information. In addition, Qualtrics which was used to host the survey automatically recorded the participants Prolific ID code, so we know that no individual took part in the survey twice.	Page 7
Log file analysis	None.	Page 6-7
Registration	Users needed to have a registered account with Prolific to take part in the study.	Page 6-7
Handling of incomplete questionnaires	Only complete questionnaires were analysed since individuals that did not fully complete were not provided with the financial reimbursement. It would be unethical to use unpaid responses in analyses.	Page 7
Questionnaires submitted with an atypical timestamp	Prolific automatically eliminated participants that took too long to take part in the survey (i.e., above 67 minutes based on researcher estimation) to allow others to take part since there is a maximum place allowance.	Page 7
Statistical correction	No statistical correction was applied.	Page 10-11

For peer review only
